# Genome-Wide Identification and Expression Analysis of *SnRK2* Gene Family in Dormant Vegetative Buds of *Liriodendron chinense* in Response to Abscisic Acid, Chilling, and Photoperiod

**DOI:** 10.3390/genes13081305

**Published:** 2022-07-22

**Authors:** Quaid Hussain, Manjia Zheng, Wenwen Chang, Muhammad Furqan Ashraf, Rayyan Khan, Muhammad Asim, Muhammad Waheed Riaz, Mona S. Alwahibi, Mohamed S. Elshikh, Rui Zhang, Jiasheng Wu

**Affiliations:** 1 State Key Laboratory of Subtropical Silviculture, Zhejiang A&F University, 666 Wusu Street, Hangzhou 311300, China; quaid_hussain@yahoo.com (Q.H.); mangazheng@stu.zafu.edu.cn (M.Z.); 2021602122047@stu.zafu.edu.cn (W.C.); waheed_riaz2007@yahoo.com (M.W.R.); rui.zhang@zafu.edu.cn (R.Z.); 2 Key Laboratory of Modern Silvicultural Technology of Zhejiang Province, Hangzhou 311300, China; 3 Department of Arctic and Marine Biology, UiT-The Arctic University of Norway, 9009 Tromsø, Norway; muhammad.f.ashraf@uit.no; 4 Key Laboratory of Tobacco Biology and Processing, Ministry of Agriculture and Rural Affairs, Tobacco Research Institute, Chinese Academy of Agricultural Sciences, Qingdao 266101, China; rayyanswb@gmail.com (R.K.); asim.ktk91@aup.edu.pk (M.A.); 5 Zhejiang Provincial Key Laboratory of Resources Protection and Innovation of Traditional Chinese Medicine, Zhejiang A&F University, Hangzhou 311300, China; 6 Department of Botany and Microbiology, College of Science, King Saud University, Riyadh 11451, Saudi Arabia; wamona015@gmail.com (M.S.A.); melshikh@ksu.edu.sa (M.S.E.)

**Keywords:** abscisic acid, chilling, photoperiod, qRT-PCR, *SnRK2* kinase family

## Abstract

Protein kinases play an essential role in plants’ responses to environmental stress signals. *SnRK2* (sucrose non-fermenting 1-related protein kinase 2) is a plant-specific protein kinase that plays a crucial role in abscisic acid and abiotic stress responses in some model plant species. In apple, corn, rice, pepper, grapevine, *Arabidopsis thaliana*, potato, and tomato, a genome-wide study of the SnRK2 protein family was performed earlier. The genome-wide comprehensive investigation was first revealed to categorize the *SnRK2* genes in the *Liriodendron chinense (L. chinense)*. The five *SnRK2* genes found in the *L. chinense* genome were highlighted in this study. The structural gene variants, 3D structure, chromosomal distributions, motif analysis, phylogeny, subcellular localization, cis-regulatory elements, expression profiles in dormant buds, and photoperiod and chilling responses were all investigated in this research. The five *SnRK2* genes from *L. chinense* were grouped into groups (I–IV) based on phylogeny analysis, with three being closely related to other species. Five hormones-, six stress-, two growths and biological process-, and two metabolic-related responsive elements were discovered by studying the cis-elements in the promoters. According to the expression analyses, all five genes were up- and down-regulated in response to abscisic acid (ABA), photoperiod, chilling, and chilling, as well as photoperiod treatments. Our findings gave insight into the *SnRK2* family genes in *L. chinense* and opened up new study options.

## 1. Introduction

Two species of large deciduous trees make up the genus *Liriodendron*, which belongs to the Magnoliaceae family. *L*. *chinense* is a native of South China, and *L*. *tulipifera* comes from eastern North America. The medications produced from the two species varied in America, China, and other countries [1]. The majestic tree *L*. *tulipifera*, also known as the tulip tree or yellow poplar, is a particular species found in the eastern United States that may grow to heights of up to 30.48 m [1]. *Liriodendron* is an excellent decorative tree for landscaping because of its straight trunk, conical crown, unusual leaf shape, and tulip-shaped blooms [2,3]. *Liriodendron* has also been frequently planted as an industrial timber species because of its quick growth and adaptable wood with superior working characteristics [3]. It is highly prized for its honey production, as a food source for wildlife, and its potential medical benefits [3]. Studies have shown that extracts from *Liriodendron* leaves have potent cytotoxic effects on tumor cell lines and inhibitory activities against farnesyl protein transferase (FPTase) and tumor cell growth [1]. Bud dormancy, a complicated physiological phenomenon that promotes plant growth, survival, and development, impacts the timing of bud break [4]. Bud dormancy is a crucial characteristic that enables temperate woody perennials to withstand harsh winter conditions. When a plant’s development is halted and its metabolic activity is decreased, it enters a state of dormancy [5,6]. Because it affects the quality of bud break, flowering, and fruiting in the spring, bud dormancy is an important stage in the phenology cycle. A temporary cessation of observable growth is called dormancy [7]. In subtropical trees, including *L. chinense*, *Torreya grandis*, *Metasequoia glyptostroboides*, *Cinnamomum chekiangense*, and *Phoebe chekiangensis*, the release of bud dormancy is controlled by photoperiod. The short day had a lower bud burst percentage and delayed the timing of bud burst when compared to the long day [8]. The timing of bud burst displays a continuous phenotypic variation typical of a quantitative attribute for a variety of forest tree species [9]. According to a recent study, only a few woody species are photoperiod sensitive [8,10]. However, a combination of photoperiod, chilling (temperature and duration), and spring or forcing temperatures regulates bud development in temperate [11] and subtropical [8,12] trees. Although bud break and the mechanisms that cause dormancy release are closely related, the underlying molecular pathways are poorly known.

In signal transduction pathways, protein kinases and phosphorylation/dephosphorylation play significant roles in identifying and transmitting stress signals to various cell areas. *SnRK* (or SNF1-related protein kinase) family members are specific serine/threonine protein kinases found widely in plants and play essential roles in various processes, including growth and development, stress defense, and hormone-mediated signalling [13,14,15,16,17,18,19]. Based on sequence similarity and C-terminal domain structural characteristics, the *SnRK* family in higher plants is divided into three subfamilies (*SnRKl*, *SnRK2*, and *SnRK3*) [13,20,21]. Many plant genomes have been found to contain the *SnRK2* gene family, including in pepper [14], rice [21], cotton [22], *Nicotiana tabacum* [23], arabidopsis [24], mungbean [25], grapevine [26], maize [27], sugarcane [28], sorghum [29], and apple [30]. Despite this fact, only a tiny portion of the *SnRK2* gene has been characterized. Three *SnRK2s*, *SnRK2.2*, *2.3*, and *2.6*, are at the heart of the arabidopsis ABA signaling network, acting as primary positive regulators of ABA signaling in response to water stress as governing seed development and dormancy [26,31,32].

Abscisic acid (ABA) is a key regulator of plant growth and development, including seed germination, maturation and dormancy, cell elongation and division, root and seedling growth, embryo maturation, leaf senescence, and fruit ripening, as well as plant resistance to severe environments [32,33,34,35]. Furthermore, it is necessary for the plant’s reaction to abiotic stressors such as high temperature, cold, drought, and salinity. Increased ABA levels are connected with plant adaptation to these environmental stressors [36]. ABA is required for seed dormancy establishment, maintenance, release, and bud endodormancy. *Arabidopsis thaliana* (*A. thaliana*) mutants lacking ABA could not develop seed dormancy [37,38]. Dormancy is influenced not just by ABA levels but also by the ABA signaling pathway. The regulatory component of the ABA receptor (RCAR)/pyrabactin resistance (PYR)/pyrabactin resistance-like (PYL) family of ABA-receptor proteins operate as negative regulators of the protein phosphatase 2c (PP2C, ABI1/ABI2) family of protein phosphatase 2c (PP2C, ABI1/ABI2). The binding of ABA to its RCAR/PYR/PYL receptors and the creation of ABA-receptor *PP2C* complexes disrupt the interaction between PP2C and SNF1-related protein kinase 2 (*SnRK2*), which negatively impacts ABA signaling [38,39]. As a result, ABA-responsive genes can be produced by activating downstream transcription factors (such as ABI4 and ABI5) and *SnRK2* [38,39]. In the literature, ABA has been suggested to have a role in regulating bud endodormancy, with ABA levels increasing in the autumn and acting as a signal of decreasing day length. Inhibition of cell proliferation and shoot growth, promotion of terminal bud set, and induction of endodormancy are all possible outcomes. In poplar buds, regulators of ABA biosynthesis (NCED3, ABA1, and ABA2), as well as ABA signal transduction components (PP2C, ABI1, and AREB3, among others), were stimulated after 3–4 weeks of short days, and ABA levels in the apex peaked [5,40,41]. However, applying ABA to grapes in the spring had minimal influence on bud break [12], and the impact of freezing on endogenous ABA levels is unknown. Chilling-induced dormancy release in birch was followed by changes in endogenous ABA levels, confirming the involvement of ABA [42,43].

The environmental factors regulate bud dormancy in *Liriodendron*, particularly describing the role of ABA, chilling, and photoperiod. In this study, we employed genomic approaches and experimental verification to describe and identify ABA *SnRK2s* receptors, which may be essential signaling regulators in *L. chinense* response to environmental conditions. There was a total of five *LchiSnRK2* genes in the *L. chinense* genome. Other agriculturally resilient plants with identified *SnRK2* genes were studied for evolutionary differences, chromosomal locations, gene structures, and conserved sequence motifs. In addition, the expression profiles of *LchiSnRK2s* in the dormant bud have been investigated at various phases of development, as well as ABA treatment, photoperiod, and chilling conditions. We also looked into the function of cis-regulatory components identified inside the promoter sequences of ABA-receptors to see how they affect hormone and photoperiod and chilling responses, growth and biological processes, and metabolic responses. *L. chinense* vegetative dormant buds that had undergone qRT-PCR were used to confirm the transcript expression of *LchiSnRK2* genes in response to ABA foliar sprays, photoperiod, and chilling. The results of this study may contribute to a better understanding of the function of ABA receptors in *L. chinense* and the molecular characterization of *LchiSnRK2* genes after the development of genetic material that aids plant stress adaptation.

## 2. Materials and Methods

### 2.1. L. chinense SnRK2 Gene Identification and Characterization

The *L. chinense* protein database (https://hardwoodgenomics.org/Genome-assembly/2630420, accessed on 5 June 2022) and the NCBI database (https://www.ncbi.nlm.nih.gov/ (accessed on 5 June 2022), PRJNA418360) were used to retrieve the genome sequences for this species [44]. The *SnRK2* genes family in *L. chinense* was discovered using the arabidopsis SnRK2 protein sequences obtained from phytozome v13 (https://phytozome-next.jgi.doe.gov/, accessed on 5 June 2022). The TAIR database (http://www.arabidopsis.org, accessed on 5 June 2022) was used to collect the protein sequences of the well-defined 11 members of the *SnRK2* family in *A. thaliana*: *AT3G50500*, *AT1G78290*, *AT1G26470*, *AT5G63650*, *AT5G66880*, *AT2G23030*, *AT4G33950*, *AT4G40010*, *AT1G60940*, *AT5G08590*, and *AT1G10940*. Using *A. thaliana SnRK2s* as a reference, the protein sequences of the *L. chinense SnRK2* family members were found in a specific genome database (https://hardwoodgenomics.org/Genome-assembly/2630420, accessed on 5 June 2022). SnRK2’s protein sequence was analyzed by the Pfam database (http://pfam.xfam.org, accessed on 5 June 2022) and matched with a domain profile (PF00069) [3].

Five (*Lchi13910*, *Lchi00543*, *Lchi25623*, *Lchi12999*, and *Lchi01348*) *SnRK2* family genes were discovered and confirmed using the *L. chinense* genome database and NCBI database (https://www.ncbi.nlm.nih.gov/) (accessed on 5 June 2022) [44,45,46].

### 2.2. SnRK2 Genes’ Chromosomal Distribution in L. chinense

We used https://hardwoodgenomics.org/Genome assembly/2630420, accessed on 5 June 2022, to determine the genome position and protein sequences of all *L. chinense SnRK2* genes, and we assessed the distribution positions of *SnRK2* genes on scaffold or chromosome. *SnRK2* genes were found on the chromosomes of *L. chinense* using MapGene2Chromosome (MG2C; http://mg2c.iask.in/mg2c v2.0/) (accessed on 5 June 2022) [44,45,46].

### 2.3. Phylogenetic Tree Construction

Protein sequences of *SnRK2* genes from *Liriodendron chinense*, arabidopsis (*A. thaliana*), tomato (*Solanum lycopersicum*), pepper (*Capsicum annuum*), potato (*Solanum tuberosum*), grape (*Vitis vinifera*), corn (*Zea mays*), rice (*Oryza sativa*), and apple (*Malus domestica*) were used to conduct the phylogenetic analysis, and to display the *Liriodendron chinense SnRK2* gene family evolution relating to other species. The protein sequences were often aligned using MEGA11 (V 6.06) software (www.megasoftware.net) (accessed on 5 June 2022). The phylogenetic tree was constructed using the neighbor-joining (NJ) method with 1000 bootstrap repetitions. Fig Tree V1.4.4, accessed on 5 June 2022, was used to visualize and edit the phylogenetic tree [2,47,48]. To analyze the evolutionary constraints of each *SnRK2* gene pair, the KaKs Calculator 2.0 (https://sourceforge.net/projects/kakscalculator2/, accessed on 5 June 2022) was used to calculate the synonymous (Ks), and non-synonymous (Ka) ratios [49].

### 2.4. Structure and Significant Motif Analyses of the SnRK2 Family Members

The genome of *L. chinense* contains five members of the *SnRK2* family. Web software that also exhibited the exon/intron arrangements of the *SnRK2* genes was used to determine the structural analyses of five *SnRK2* genes (http://gsds.cbi.pku.edu.cn, accessed on 5 June 2022). In the protein sequences of the five SnRK2 proteins, the online application MEME v5.4.1, Available online: https://meme-suite.org/meme/tools/glam2scan (accessed on 5 June 2022), revealed more conserved strings or regions [3]. Sequence alphabet DNA, RNA, or protein; site distribution zero or one occurrence per sequence (zoops); motif finding mode classic mode; and 10 motifs were the settings utilized by the application. The TBtools program displayed the MEME results by downloading the supplementary mast file [50,51].

### 2.5. Analysis of the SnRK2 Family’s Promoter Sequences in L. chinense

We collected 2500 bp upstream sequences of *SnRK2* family members using the *L. chinense* genome assembly database (https://hardwoodgenomics.org/Genome assembly/2630420) (accessed on 5 June 2022). To find cis-regulatory elements (CREs), the obtained sequences were then examined by PlantCARE (http://bioinformatics.psb.ugent.be/webtools/plantcare/html/) (accessed on 5 June 2022). After calculating the frequency of each CRE motif, we used TBtools to find the most common CREs for the SnRK2 genes [51].

### 2.6. 3D Structure and Subcellular Localization

We utilized SWISS-MODEL (https://swissmodel.expasy.org/interactive, accessed on 5 June 2022) to estimate the three-dimensional (3D) structure [2]. We utilized Cell-PLoc 2.0 (http://www.csbio.sjtu.edu.cn/bioinf/Cell-PLoc-2/, accessed on 5 June 2022) to predict the subcellular localization of the *SnRK2* family of genes [46].

### 2.7. Plant Material and Environmental Conditions

**Figure 1:**
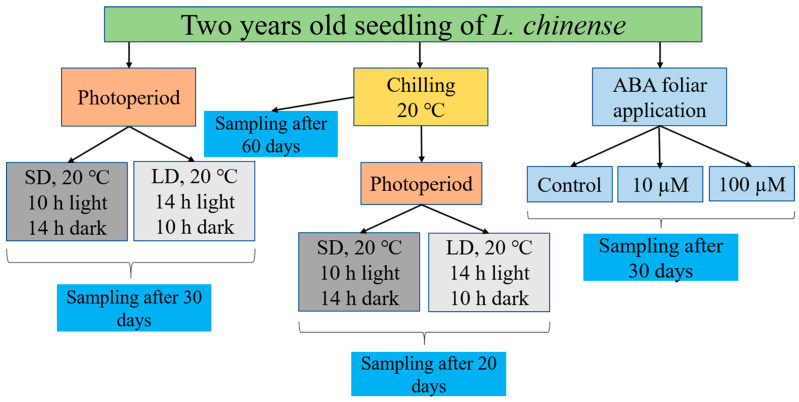
This schematic flow depicts the experimental setup. The various box designs and color schemes represent different modes of treatment and sampling.

In order to comprehend the effects of various abscisic acid concentrations (ABA-10 µM and ABA-100 µM), photoperiods (long day and short-day), chilling, and chilling as well as photoperiod during the dormant vegetative bud, tests were carried out at the Zhejiang Agricultural and Forestry University (Hangzhou, China). We transferred two-year-old *L. chinense* seedlings from the Tianmushan National Forest Station nursery in Hangzhou, China, to our university’s growing chamber to conduct our experiment. The seedlings were relocated from the nursery to the growth chamber for photoperiod and chilling treatments, where they were kept at 20 °C (day/night), 200 mol m^−2^ s^−1^ of light intensity, etc., and 50% relative humidity (Figure 1) [2,8].

### 2.8. Extraction of RNA and qRT-PCR Analysis

We used the Total RNAPlant Extraction Kit as directed by the manufacturer (Tiangen, Beijing, China). The cDNA was produced using the TaKaRa PrimeScript 1st Strand cDNA Synthesis Kit (TaKaRa, Dalian, China), following the manufacturer’s instructions. The SYBR Green Real-time PCR Master Mix (TOYOBO, Osaka, Japan) was used to perform the qRT-PCR on an ABI7500 Real-Time PCR System (Applied Biosystems, Foster City, CA, USA) [35].

The transcript profiles of *Lchi13910*, *Lchi00543*, *Lchi25623*, *Lchi12999*, and *Lchi01348* in the dormant bud subjected to various environmental conditions were validated by qRT-PCR analysis (Figure 1). The three technical replicates were used to conduct the qRT-PCR. As a reference gene, the actin gene *LtActin97* from *Liriodendron* was used, along with the sequence provided by Zong et al. [52], to calculate the target genes’ expression levels using the 2^−ΔΔCT^ technique [53]. Primer Premier 5.0 software was used in this investigation to target all employed primers using CDS sequences of *SnRK2*; more information is available in Supplementary Table S1. GraphPad Prism 9.0.0 software was used to display the outcomes of the qRT-PCR analysis [2].

### 2.9. Gene Duplication Analysis

We utilized the Riaz et al. [54] criterion to discover duplicated gene pairs: (1) the nucleotide sequence that we aligned spanned >78% of the longer aligned gene, and (2) the identity between the aligned portions must be exceeded >78%.

### 2.10. Statistical Analysis

The data were analyzed by Statistix 8.1 software (Analytical Software, Tallahassee, FL, USA) using one-way ANOVA, and the data were presented as the mean ± SD (Standard deviation) of the three replicates. The differences in the mean values of the dormant buds between photoperiod, chilling, chilling, as well as photoperiod, and different treatments (ABA-10 µM and ABA-100 µM) of ABA plants were analyzed using an LSD (least significant difference) test at *p* < 0.05. The graphs were made using GraphPad Prism 9 statistical software (https://www.graphpad.com/) (accessed on 5 June 2022) [55].

## 3. Results

### 3.1. Identification of the SnRK2 Gene Family in L. chinense

Using queries of the well-defined protein sequences of 11 *SnRK2s* from the *A. thaliana* genome, we investigated five *SnRK2* genes in the *L. chinense* genome (Table 1 and Table S2). We found a lower number than the previously described *SnRK2* genes in other species, e.g., the apple, grapevine, arabidopsis, rice, tomato, potato, corn, and pepper (Table S3). Four proteins (Lchi13910, Lchi25623, Lchi12999, and Lchi01348) were determined to have only one Protein kinase domain, according to domain analysis (PF00069), and for one protein (Lchi00543), we did not find any domain, respectively (Table S4).

**Figure 2:**
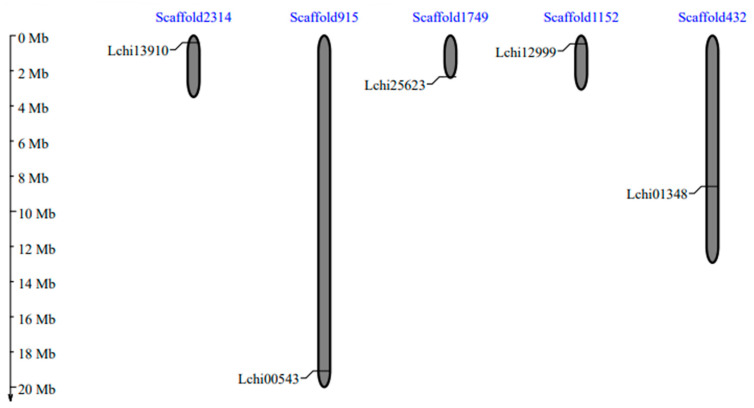
The *SnRK2* gene distribution on the chromosomes of *L. chinense* serves as a scaffold. The chromosomal/scaffold numbers are found at the top of each chromosome. The names of each *LcSnRK2* gene are displayed on the left side of each chromosome. The bars on the scaffold/chromosomes represent the *SnRK2* genes.

Table 1 contains comprehensive statistics for five *SnRK2* genes. All five *SnRK2s* genes, including *Lchi13910*, *Lchi00543*, *Lchi25623*, *Lchi12999*, and *Lchi01348*, were localized on different chromosome/scaffolds (Figure 2; Table 1), respectively. There were explicit mentions of the lengths of the genomic sequence, coding sequence, proteins, gene strands, and exons, respectively (Table 1). According to the results of subcellular localization, all proteins were expected to be found in the nucleus (Table 1).

### 3.2. SnRK2 Gene Phylogenetic Relationships

**Figure 3:**
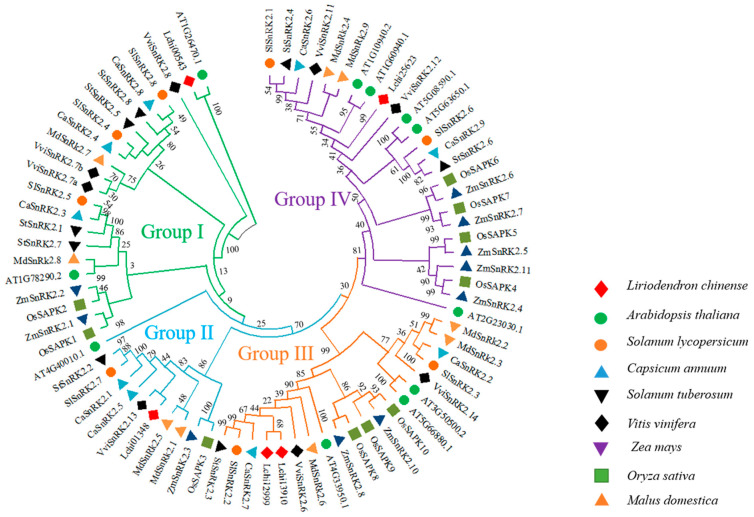
A phylogenetic analysis of SnRK2 proteins from *Liriodendron chinense* (5), *Arabidopsis thaliana* (11), *Solanum lycopersicum* (8), *Capsicum annuum* (9), *Solanum tuberosum* (8), *Vitis vinifera* (8), *Zea mays* (10), *Oryza sativa* (10), and *Malus domestica* was carried out using the maximum likelihood method (9). There are four groups of SnRK2 proteins: I, II, III, and IV, each of which is represented by a different color.

*Liriodendron chinense* predicted SnRK2 proteins were examined using multiple sequence alignment of arabidopsis, tomato, pepper, potato, grape, corn, rice, and apple were shown that a phylogenetic tree in (Table S3) showed four important groups (Group I to IV) (Figure 3). According to the findings, group I consisted of 22 SnRK2 members, Group II included 11 SnRK2 members, Group III contained 20 SnRK2 members, and Group IV comprised 25 SnRK2 members (Figure 3). It is important to note that homologs of *LchiSnRK2s* genes from different plant species were discovered in each group, with Group III having the highest number of individuals in Groups I, II, and IV (Figure 3). The *LchiSnRK2s* also share a closer evolutionary connection with the other species within each group.

### 3.3. Liriodendron chinense SnRK2 Genes Structures and Conserved Motifs Investigation

**Figure 4:**
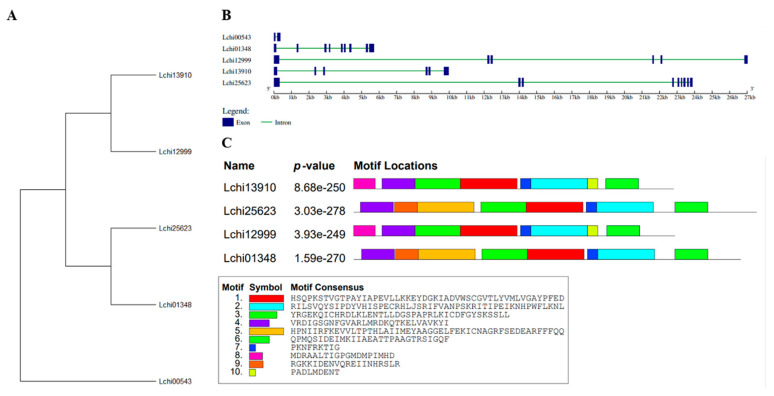
*L. chinense’s SnRK2* family gene structure and motif analysis: (**A**) Based on phylogenetic relationships and domain identification, the *SnRK2s* were classified into four groups. Gene structure for *SnRK2* (**B**). The blue horizontal line denotes exon regions, while the green horizontal line denotes intron regions. (**C**) Conserved motif compositions were found in *L. chinense SnRK2s*. Various color boxes represent numerous motifs.

**Figure 5:**
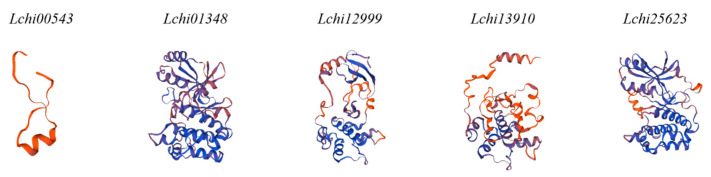
*SnRK2* family 3D structures displaying functional sites.

The gene expansion of the *L. chinense* family was investigated by examining the exon-intron patterns of the *SnRK2* genes (Tables S5 and S6). To further comprehend the structural characteristics of *SnRK2* genes, the exon–intron structures and conserved motifs (Figure 4A–C) were examined. The *SnRK2s*’ exons and introns varied from 1 to 8 and 2 to 9, respectively (Figure 4B). The *SnRK2* gene family has a variety of gene architectures, with most *SnRK2* genes having five to eight introns; however, certain *SnRK2* gene family members, such as *Lchi00543*, have fewer introns. The maximum number of exons and introns found in *Lchi25623* and *Lchi01348* was nine. These findings demonstrated that a gene structure that was highly similar to that of their evolutionary relatives was shared by a group of *SnRK2* individuals. The SWISS-MODEL program was used to estimate the three-dimensional (3D) structure, and the projected 3D structures showed that the LchiSnRK2 protein has similar conserved structures. Figure 5 provides comprehensive details on the predicted 3D structures.

Additionally, we used the MEME online servers to clarify the conserved motifs of the *SnRK2* genes. Furthermore, five *SnRK2* genes included 10 conserved motifs (Figure 4C). Due to the prediction, motifs one, two, three, four, six, and seven in most SnRK2 proteins. Motifs one, two, three, four, six, and seven were recognized in *Lchi13910*, *Lchi25623*, *Lchi12999*, and *Lchi01348*. Motifs five and nine were identified in *Lchi25623*, and *Lchi01348*, while motifs eight and 10 were recognized in *Lchi13910*, and *Lchi12999*, respectively. SnRK2 proteins exhibited three highly conserved motifs (one, two, and five) with 50 amino acids; one motif (three) with 40 amino acids, while two motifs (four and six) with 29 amino acids, motif nine with 20, motif eight with 19, and motifs seven and 10 with nine amino acids (Figure S1). These findings suggest that the gene structure and amino acid sequence of members of the same subfamily of *LchiSnRK* share a significant degree of similarity. LchiSnRK proteins’ three-dimensional (3D) structures show that three subfamily proteins have similar 3D structures at their N terminals but differ at their C terminals (Figure 5).

### 3.4. Identification of Cis-Regulatory Elements in the Promoters of Five SnRK2 Genes

**Figure 6:**
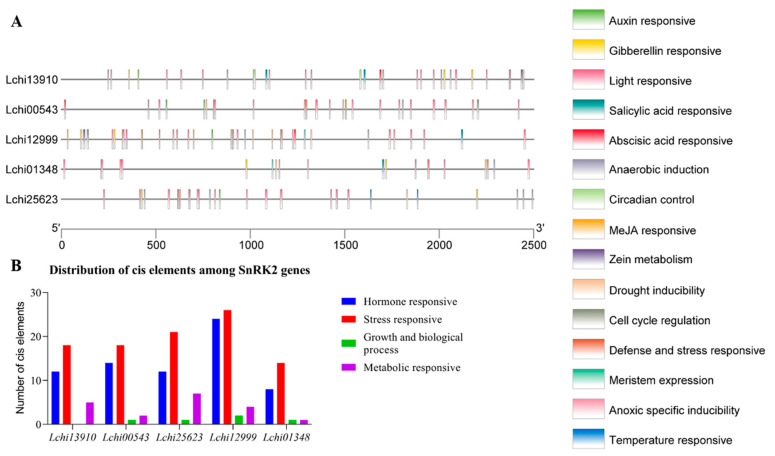
Cis-regulatory elements (CREs) found in the promoters of *SnRK2* genes. (**A**) The positional distribution of the projected CREs on the *SnRK2* promoters is shown by vertical bars. Five *SnRK2* genes’ promoter sequences (2500 bp) were examined using PlantCARE. In this legend, each cis-color element is represented: (**B**) Hormones, stress, growth and biological processes, and sensitive metabolic components are linked to the distribution of cis-elements in the promoters of *SnRK2* genes. The detected cis-elements are displayed in colored boxes.

**Figure 7:**
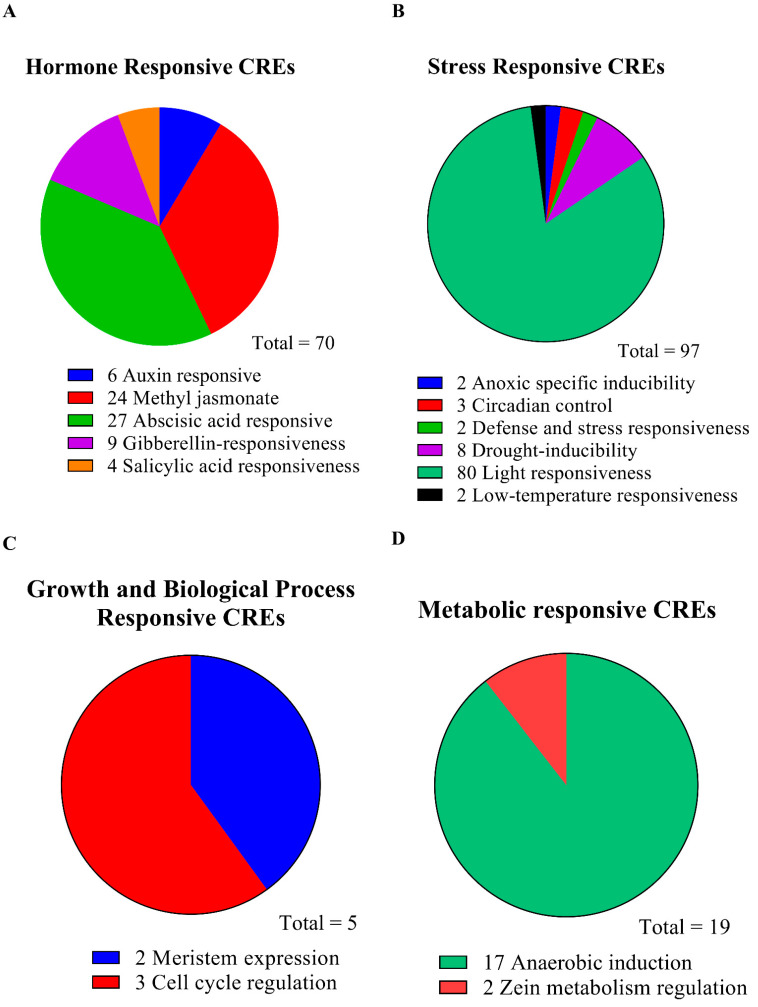
The distribution of cis-regulatory elements (CREs) in the promoters of *SnRK2* genes is based on their hypothesized roles. The four different types of CREs are (**A**) hormone-responsive CREs, (**B**) stress-responsive CREs, (**C**) growth and biological process-responsive CREs, and (**D**) metabolic-responsive CREs.

Using a 2500 bp region from each gene’s TAS (transcriptional activation site) to analyze the PlantCARE database to determine the cis-regulatory elements and functions of the genes. In the chosen region of the *SnRK2* genes’ promoters in *L. chinense*, we identified possible cis-elements (Table S7). In *SnRK2s*, several cis-regulatory components are displayed in Figure 6A,B. On the items that were found, there is extensive information in Table S8. The *SnRK2s* gene family comprises 191 distinct CREs, including elements sensitive to hormones, stress, growth and biological processes, and metabolism (Figure 6B). Identifying five hormone-related responsive elements—ABA, auxin, GA, MeJA, and SA—represents and suggests the right targets for research into how hormones work in stressful situations (Figure 6; Table S8). The majority of hormone-related responsive regions are specific to a few genes, as seen in Figure 5. Five genes include multiple copies of the responsive elements for auxin, ABA, SA, MeJA, and GA, illustrating their critical roles in phytohormone-related responses in plants (Figure 7A–D). These *SnRK2s* genes may respond to stress-related stimuli as evidenced by the prediction of six stress-related response components (anoxic specific inducibility, circadian regulation, defense and stress, drought, light, and low temperature) (Figure 7A–D; Table S8). In addition, 80 light-responsive components were discovered, suggesting that *SnRK2s* are essential for responding to light stress. All *SnRK2* genes had one defensive and stress-response element, two anoxic specific inducibility, three circadian control, eight drought response elements, and two low-temperature response elements (Figure 7; Table S5). Most of the promoters of the *SnRK2* genes contained CREs responsive to biological processes and growth, including meristem expression (two) and cell cycle regulation (three). In addition, two sensitive metabolic elements—one each for the control of zein metabolism and anaerobic induction—were found (Figure 7A–D; Table S8).

### 3.5. SnRK2 Gene Expression Analysis Using Real-Time qRT-PCR in Response to Dormant Bud in L. chinense

**Figure 8:**
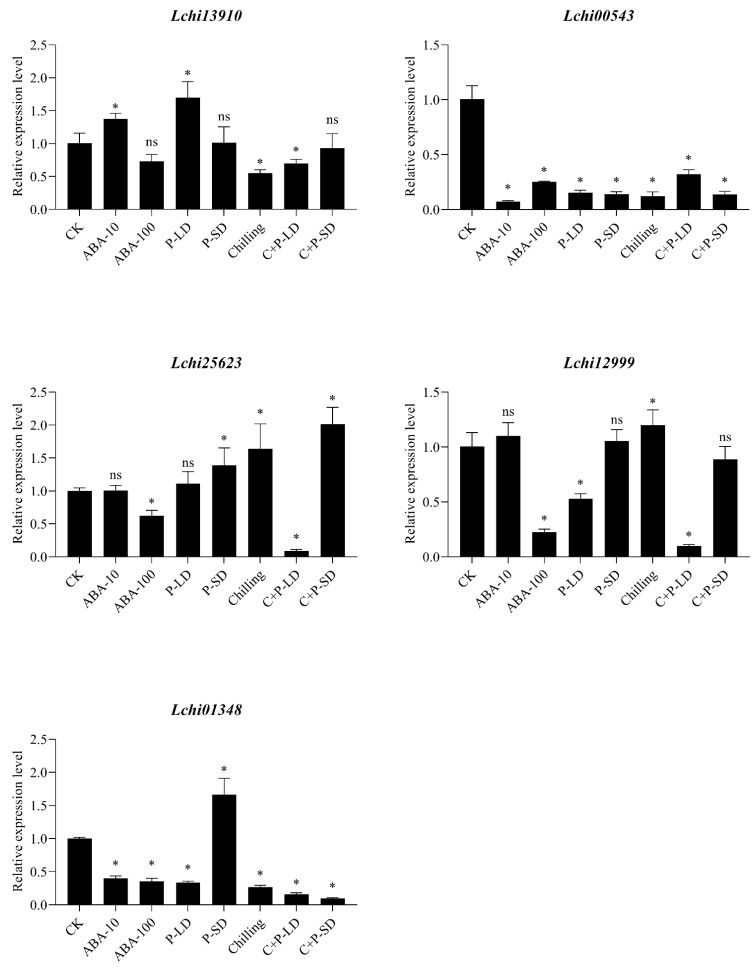
The expression of *LchiSnRK2s* by qRT-PCR in dormant buds of *L. chinense* at the seedling stage under different photoperiods (long day (P-LD) and short-day (P-SD)), chilling, various abscisic acid concentrations (ABA-10 µM and ABA-100 µM), and chilling as well as photoperiod (C + P-LD, C + P-SD). The Least Significant Difference (LSD) test indicates a significant (*p* ≤ 0.05) difference between the control and all conditions. * = Showed significant differences and ns = Showed non-significant differences.

Five *LchiSnRK2* genes were subjected to qRT-PCR-based expression profiling to look into their expression profiles under contrasting photoperiods (long day (P-LD) and short-day (P-SD)), chilling, various abscisic acid concentrations (ABA-10 µM and ABA-100 µM), and chilling as well as photoperiod (C + P-LD, C + P-SD) conditions (Figure 8). The expression level of the *Lchi13910* gene significantly increased under ABA-10 µM and photoperiod long day (P-LD) and significantly decreased under chilling as well as photoperiod long day (C + P-LD), according to the RT-qPCR data. In other conditions, including ABA-100 µM, P-SD, and C + P-SD, *Lchi13910* expression level displayed a non-significant difference. Similarly, the expression of the *Lchi00543* gene significantly decreased under all environmental circumstances, including chilling, photoperiod, and various ABA concentrations. In P-SD, chilling, and C + P-LD conditions, the expression level of *Lchi25623* was significantly up-regulated, whereas, in ABA-100 µM conditions, it was significantly downregulated. In the ABA-10 µM and P-LD conditions, *Lchi25623* displayed non-significant differences. The Lchi12999 gene was significantly up-regulated in the chilling condition, whereas it was down-regulated in the ABA-100 µM, P-LD, and C + P-LD conditions. In the ABA-10 µM, P-SD, and C + P-SD conditions, the expression level of *Lchi12999* did not display any significant differences. The *Lchi01348* transcript expression was also highly up-regulated in P-SD but significantly down-regulated in all other environmental conditions (Figure 8).

## 4. Discussion

Only two species of the genus *Liriodendron* exist in nature, *L. chinense* and *L. tulipifera*. It is an extinct plant. These are commonly planted for landscaping and timber production in China and the US due to their great material qualities and ornamental value [44,46]. China’s central-western and southern regions are home to *L. chinense* (Hemsl.) Sarg. (*L. chinense*), which is typically found in the mountains at altitudes between 450 and 1800 m [56]. Researchers studying the disjunctive distribution of flowering plants between eastern Asia and eastern North America have shown that *Liriodendron* is an ideal natural resource [57]. Numerous items, including furniture and agricultural machinery, are made from *Liriodendron* wood [1]. Several studies have demonstrated the powerful cytotoxic effects of extracts from *Liriodendron* leaves on tumor cell lines, as well as their inhibitory activity against farnesyl protein transferase (FPTase) and tumor cell development. [1,56] Despite *Liriodendron’s* high economic worth and *L. chinense’s* threatened status, little is known about their population’s genetic makeup and geographic variation [56]. ABA appears crucial for bud dormancy induction and maintenance but not for dormancy release. Bud dormancy-related gene expression in stratified peach seeds was studied with measurements of ABA levels to look into the relationship between seed and bud dormancy [58]. In numerous plant species, ABA has been shown to perform a notable role in inducing seed and bud dormancy and controlling stress responses [59]. For perennial plants to survive, the transition from bud dormancy is an essential developmental process [59]. Diverse endogenous genetic variables and environmental cues control the process; however, the underlying mechanisms are still poorly understood. According to other studies, ABA is only required for bud growth and maintenance, not for the release of dormancy [59]. In *Liriodendron* twigs, photoperiod substantially impacted the budburst percentage (BB%) and interacted with chilling [8]. The BB% in *Liriodendron* twigs responded strongly to photoperiod. In this species, the BB% under LD conditions was consistently higher than 50%, but under SD conditions, it was lower than 20%. This was true regardless of the length of the chilling period [8]. The high sensitivity to photoperiod would delay or prevent bud bursting during warm winters (high sensitivity of days to budburst, *Liriodendron* twigs, *Phoebe*, and *Torreya* seedlings), lowering the risk of frost damage brought on by false springs [8].

There are numerous forms of unfavorable environmental conditions to which plants are subjected. Plants can mitigate the negative impacts of extreme environmental conditions by utilizing various morphological, physiological, and molecular defense mechanisms [25,60]. According to Gillebert et al. [61], protein-kinase-mediated phosphorylation is crucial for reducing ecological pressures, and the *SnRK2* genes, as well-known members of the serine-threonine protein kinase subfamily, are essential for combating environmental challenges such as drought [62,63]. *SnRK2s* mostly regulate the development of abscisic acid-dependent plants and their responses to environmental stressors. *SnRK2s* are either not activated or weakly activated in plants in response to ABA regulating abiotic stress responses. Furthermore, *SnRK2* genes are essential controllers of the ABA signal-transduction pathway [64,65,66]. Fortunately, *L. chinense* whole-genome sequencing data are now accessible [44], which presents a fantastic chance to investigate this gene family at the genome level.

According to numerous research, each *SnRK2* gene uniquely responds to multiple environmental conditions. The majority of *SnRK2s* in model plant species such as rice [21], arabidopsis [24], and maize [27] have been described to date. The first *SnRK2* gene was discovered from an ABA-treated wheat embryo cDNA library [67]. Many plant species have been found to contain *SnRK2* genes. However, the *SnRK2* gene family in *L. chinense* had not yet been identified. Five distinct *SnRK2* genes were detected in the *L. chinense* genome by genome-wide analysis in the current study. This is fewer than the number of *SnRK2* genes found in other plant species, such as 20 in cotton [22], 22 in *N. tabacum* [23], 10 in arabidopsis [24], 10 in rice [21], eight in mungbean [25], eight in grapevine [26], 11 in maize [27], 10 in sugarcane [28], 10 in sorghum [29], nine in pepper [13], and 12 in apple [30]. According to published research, 8 to 22 *SnRK2* genes have been reported for several plant species.

The gene structure of *LchiSnRK2s* was analyzed and revealed two patterns. Two genes had nine exons and eight introns, while two other genes had six exons and five introns, and only one gene had two exons and one intron. The former gene structure pattern is consistent with those of cotton [22], *N. tabacum* [23], arabidopsis [24], rice [21], mungbean [25], grapevine [26], maize [27], sugarcane [28], sorghum [29], pepper [13], and apple [30]. The *SnRK2* family in plants has undergone very little evolutionary change.

A thorough phylogenetic analysis was carried out by building a phylogenetic tree to explore the evolutionary relationship between *L. chinense, A. thaliana, Solanum lycopersicum, Capsicum annuum, Solanum tuberosum, Vitis vinifera, Zea mays, Oryza sativa,* and *Malus domestica*. The phylogenetic analysis showed that the *LchiSnRK2* genes were grouped into the four previously described groups (I, II, III, and IV) [24,26,66]. *Lchi00543* was the only member of the group I, and *Lchi01348* was the only member of group II. *Lchi13910* and *Lchi12999* were the two members of group III, and *Lchi25623* was in Group IV. These findings demonstrate a potential evolutionary pattern within the *SnRK2* gene family [25].

The cis-regulatory motifs and elements present in the promoter sequences of the *LchiSnRK2* genes will help us better understand how these genes react to different environmental situations. Our findings revealed four types of cis-elements, including those that responded to hormones, stress, growth and biological processes, and metabolism (Figure 6 and Figure 7; Table S8). Light, ABA, methyl jasmonate, anaerobic induction, gibberellin, drought, auxin, salicylic acid, circadian control, cell cycle regulation, defenses and stress, low temperature, meristem, and other extreme cis-elements were among them. Previous reviews show cis-elements promote plant stress responses [22,23,24]. *SnRK2* genes were shown to have a significant role in specific agricultural plants under various environmental conditions by others who also reported comparable findings in these same plants [2]. These findings may improve our understanding of *LchiSnRK2* genes in diverse contexts and conditions. The possible functionalities of various *LchiSnRK2* were also characterized using 3D structural modeling. The *LchiSnRK2* box, which forms a single helix and is packed parallel to the -helix in the N-terminal lobe, is a notable feature of *LchiSnRK2*. For kinase activity, the *LchiSnRK2* box-C contact has been essential [68]. Understanding the biological functions of LchiSnRK2 proteins is possible by their modeled 3D structures.

Numerous investigations have shown that *SnRK2s* have a role in various abiotic stress responses. This study looked at how *LchiSnRK2* expression changed in response to ABA, chilling, and photoperiod treatments. ABA also increased the expression of numerous *SnRK2* genes from maize [27]. Numerous *SnRK2* genes are differently controlled by ABA in various organs of the rice plant [21]. Wheat *TaSnRK2.4*, *TaSnRK2.7*, and *TaSnRK2.8* expression levels are increased in response to various stressors [62]. *SnRK2s*, such as *TaSnRK2.3*, *TaSnRK2.4*, *TaSnKR2.7*, and *TaSnRK2.8* from wheat [69,70,71]; and *ZmSnRK2.3* and *ZmSnRK2.7* from maize, have been demonstrated to Huai et al. [27]. Additionally, *LchiSnRK2* genes may be effective genetic modifiers for chilling, photoperiod, and various ABA levels tolerance in plants and crops.

## 5. Conclusions

In conclusion, five *LchiSnRK2* genes were identified during genome-wide analysis of *L. chinense SnRK2* genes. The *SnRK2* gene family in *L. chinense* was subjected to a genome-wide investigation, and the expression patterns in dormant vegetative buds under ABA foliar sprays, photoperiod, and chilling conditions were studied. Through molecular breeding, it may be possible to increase the photoperiod, chilling tolerance, and bud dormancy release of *L. chinense* and other crops. Our understanding of the mechanism behind *L. chinense* stress tolerance may be aided by the molecular elucidation of these genes.

## Figures and Tables

**Table 1:** Characteristics of the five *SnRK2* genes identified in *L. chinense*. Note: GSL—Genomic sequence length, CDSL—Coding sequence length, PSL—Protein sequence length, SCL—Subcellular localization.

Gene ID	Chromosome	GSL	CDSL	PSL	SCL	Exon	Intron	Ka/Ks Value
*Lchi13910*	Scaffold2314	9980	849	282	Nucleus	6	5	0.09
*Lchi00543*	Scaffold915	369	267	88	Nucleus	2	1	0.99
*Lchi25623*	Scaffold1749	23,880	1068	355	Nucleus	9	8	0.18
*Lchi12999*	Scaffold1152	27,019	852	383	Nucleus	6	5	0.09
*Lchi01348*	Scaffold432	5717	1026	341	Nucleus	9	8	0.18

## Data Availability

The data presented in this study are available on request from the corresponding author.

## Supplementary Materials

The following supporting information can be downloaded at: https://www.mdpi.com/article/10.3390/genes13081305/s1, Figure S1. The details of the 10 motifs found in LchiSnRK2 proteins. Table S1. Details about the primers used in this study’s qRT-PCR gene expression investigation. Table S2. Arabidopsis protein detected by a blast in L. chinense. Table S3. Information on the protein sequences of the nine species’ known SnRK2 members. Table S4. List of identified domains. Table S5. Information of CDS sequence of SnRK2 family in Liriodendron chinense. Table S6. Genomic sequence of SnRK2 family in L. chinense. Table S7. Promoter sequence of SnRK2 family in Liriodendron chinense. Table S8. Information of cis-elements detected in the promoter’s regions of SnRK2s family.
