# Supplementary material for: Genome-Wide Identification and Expression Analysis of SnRK2 Gene Family in Dormant Vegetative Buds of Liriodendron chinense in Response to Abscisic Acid, Chilling, and Photoperiod
Source: Genes (Basel). 2022 Jul 22;13(8):1305. doi: 10.3390/genes13081305 (PMC9331246; doi:10.3390/genes13081305)
Supplement: Supplementary file 1 [file genes-13-01305-s001.zip › Table S1.pdf]

Table S1. Details about the primers used in this study's qRT-PCR gene expression investigation.

Gene name	Forward primer	Reverse primer
LtActin97	TTCCCGTTCAGCAGTGGTCG	TGGTCGCACAACTGGTATCG
Lchi13910	GATCGATCGGCGGTGAC	ACGAGCTCCTTGGTCTG
Lchi00543	GGTGGAATTGGAATTGAGAG	GGCAATGAGAGTACAGGGTAG
Lchi25623	CGCACGACCACACATCAG	CAAGTCCTCTCTGCTTCA
Lchi12999	CACATTGCGTAGATGATCTC	TACCTGCGGCTGGGACCC
Lchi01348	GGAGGAGAGATATGAGCC	CCCTCTGCACGTTCTCAT
