# Supplementary material for: Genome-Wide Identification and Expression Analysis of SnRK2 Gene Family in Dormant Vegetative Buds of Liriodendron chinense in Response to Abscisic Acid, Chilling, and Photoperiod
Source: Genes (Basel). 2022 Jul 22;13(8):1305. doi: 10.3390/genes13081305 (PMC9331246; doi:10.3390/genes13081305)
Supplement: Supplementary file 1 [file genes-13-01305-s001.zip › Table S3.pdf]

Table S3. Information on the protein sequences of the nine species' known SnRK2 members.

Liriodendron chinense

>Lchi13910
MDRSAVTIGPGMDMPIMHDGDRYELVRDIGSGNFGVARLMRDKQTKELVAVK
YIERGEKQVCHRDLKLENTLLDGSPAPRLKICDFGYSKSSVLHSQPKSTVGTPAYI
APEVLLKKEYDGKIADVWSCGVTLYVMLVGAYPFEDPDEPKNFRKTIQRILSVQ
YSIPDYVHISPECRHLISRIFVANPTMRITIPEIQNHEWFLKNLPADLMDDNTSQYD
EPDQPMQSIDEIMKIIAEATIPAAGTHGINQYITGSLDFDDDMDLETDPDIDVDSSG
EIVYAM

>Lchi00543
MENAGEEEEQDGLSVHSPGQAPPSSASSLPKEHSQVELELRVLEALEIYPPSKLQG
IFAINWFEISVLESLCFDIQNIEKLPCTLIAH

>Lchi25623
MEKYELVRDIGSGNFGVARLMRNKDTRELVAMKYIERGHKIDENVAREIINHRS
LRHPNIIRFKEVVLTPTHLAIVMEYAAGGELFERICNAGRFSEDEARYFFQQLISG
VSYCHSMQICHRDLKLENTLLDGSPAPRLKICDFGYSKSSLLHSRPKSTVGTPAYI
APEVLSRREYDGKLADVWSCGVTLYVMLVGAYPFEDQEDPKNFRKTIGRIMAV
QYKIPDYVHISQECRQLLSRIFVANSSKRITIKEIKSHPWFLKNLPRELTETQQAIY
YQRDNPSFSLQSVDDIMKIVGEARTPPPSSRSIGGFGWVGEEEEEEKEEDVDQEEE
EDEEDEYDKRVKEVHASGEFHLS

>Lchi12999
MDRAALTVGPAMDMPIMHDSDRYELVRDIGSGNFGVARLMRDKQTRELVAVK
YIERGEKQVCHRDLKLENTLLDGSPAPRLKICDFGYSKSSVLHSQPKSTVGTPAYI
APEVLLKKEYDGKIADVWSCGVTLYVMLVGAYPFEDPEEPKNFRKTIQRILSVQ
YSIPDYVHISPECQHLISRIFVANPATRITIPEIRNHEWFLKNLPADLMDENTVNQF
EEPDQPMQSIDEIMQIIAEATIPAAGTRSINQFLIGEDLDDDMEDLESDPELDVDSS
GEIIYAM

>Lchi01348
MEERYEPLKELGSGNFGVARLVRDKKTKELVAVKYIERGKKIDENVQREIINHRS
LRHPNIVRFKEVVLTPTHLAIIMEYAAGGELFEKISSAGRFSEDEARFFFQQLISGV
SYCHSMEICHRDLKLENTLLDGSPTPRLKICDFGYSKSALLHSQPKSTVGTPAYIA
PEVLSRKEYDGKIADVWSCGVTLYVMLVGAYPFEDPEDPRNFRKTIGRIMTVQY
SIPDYIRTSAECRHLLSRIFVADPSKRIAIPEIKNHPWFLKNLPRELIDYEKTNYENV
GSDSLSQSVEEIMRIIQEAGTPGEGLKVDGQSVAGLVDPDDTDTDIDTEEVDESG
DFVARV

Arabidopsis thaliana

>AT1G10940.2

MDKYELVKDIGAGNFGVARLMKVKNSKELVAMKYIERGPKIDENVAREIINHRS
LRHPNIIRFKEVVLTPTHLAIAMEYAAGGELFERICSAGRFSEDEEEGNKRKHARY
FFQQLISGVSYCHAMQICHRDLKLENTLLDGSPAPRLKICDFGYSKSSLLHSRPKS
TVGTPAYIAPEVLSRREYDGKMADVWSCGVTLYVMLVGAYPFEDQEDPKNFRK
TIQKIMAVQYKIPDYVHISQDCKNLLSRIFVANSLKRITIAEIKKHSWFLKNLPREL
TETAQAAYFKKENPTFSLQTVEEIMKIVADAKTPPPVSRSIGGFGWGGNGDADG
KEEDAEDVEEEEEEVEEEEDDEDEYDKTVKEVHASGEVRIS

>AT1G26470.1

MGGRTDESGGGGKEKEEEQDGLSVHSPCKALPSSASSLSKEQSQVELELRLLEAL
EIYPPVKLRGIHRHFVLYGLMEYLGRSFDRPFTADEVLQLLDRFYNIEMLKSDDE
DIDILNHEEDFTLPQSFFDKEEE

>AT1G60940.1

MDKYELVKDIGAGNFGVARLMRVKNSKELVAMKYIERGPKIDENVAREIINHRS
LRHPNIIRFKEVVLTPTHIAIAMEYAAGGELFERICSAGRFSEDEARYFFQQLISGV
SYCHAMQICHRDLKLENTLLDGSPAPRLKICDFGYSKSSLLHSMPKSTVGTPAYI
APEVLSRGEYDGKMADVWSCGVTLYVMLVGAYPFEDQEDPKNFKKTIQRIMAV
KYKIPDYVHISQDCKHLLSRIFVTNSNKRITIGDIKKHPWFLKNLPRELTEIAQAAY
FRKENPTFSLQSVEEIMKIVEEAKTPARVSRSIGAFGWGGGEDAEGKEEDAEEEV
EEVEEEEDEEDEYDKTVKQVHASMGEVRVS

>AT1G78290.2

MERYEIVKDIGSGNFGVAKLVRDKFSKELFAVKFIERGQKIDEHVQREIMNHRSL
IHPNIIRFKEVLLTATHLALVMEYAAGGELFGRICSAGRFSEDEARFFFQQLISGV
NYCHSLQICHRDLKLENTLLDGSEAPRVKICDFGYSKSGVLHSQPKTTVGTPAYI
APEVLSTKEYDGKIADVWSCGVTLYVMLVGAYPFEDPSDPKDFRKTIGRILKAQ
YAIPDYVRVSDECRHLLSRIFVANPEKRITIEEIKNHSWFLKNLPVEMYEGSLMM
NGPSTQTVEEIVWIIEEARKPITVATGLAGAGGSGGSSNGAIGSSSMDLDDLDTDF
DDIDTADLLSPL

>AT2G23030.1

MEKYEMVKDLGFGNFGLARLMRNKQTNELVAVKFIDRGYKIDENVAREIINHR
ALNHPNIVRFKEVVLTPTHLGIVMEYAAGGELFERISSVGRFSEAEARYFFQQLIC
GVHYLHALQICHRDLKLENTLLDGSPAPRLKICDFGYSKSSVLHSNPKSTVGTPA
YIAPEVFCRSEYDGKSVDVWSCGVALYVMLVGAYPFEDPKDPRNFRKTVQKIM
AVNYKIPGYVHISEDCRKLLSRIFVANPLHRSTLKEIKSHAWFLKNLPRELKEPAQ
AIYYQRNVNLINFSPQRVEEIMKIVGEARTIPNLSRPVESLGSDKKDDDEEEYLDA
NDEEWYDDYA

>AT3G50500.2

MDPATNSPIMPIDLPIMHDSDRYDFVKDIGSGNFGVARLMTDRVTKELVAVKYIE
RGEKIDENVQREIINHRSLRHPNIVRFKEVILTPSHLAIVMEYAAGGELYERICNA
GRFSEDEARFFFQQLISGVSYCHAMQICHRDLKLENTLLDGSPAPRLKICDFGYSK
VLFISLKSSVLHSQPKSTVGTPAYIAPEILLRQEYDGKLADVWSCGVTLYVMLVG
AYPFEDPQEPRDYRKTIQRILSVTYSIPEDLHLSPECRHLISRIFVADPATRITIPEITS
DKWFLKNLPGDLMDENRMGSQFQEPEQPMQSLDTIMQIISEATIPTVRNRCLDDF
MADNLDLDDDMDDFDSESEIDVDSSGEIVYAL

>AT4G33950.1

MDRPAVSGPMDLPIMHDSDRYELVKDIGSGNFGVARLMRDKQSNELVAVKYIE
RGEKIDENVKREIINHRSLRHPNIVRFKEVILTPTHLAIVMEYASGGELFERICNAG
RFSEDEARFFFQQLISGVSYCHAMQVCHRDLKLENTLLDGSPAPRLKICDFGYSK
SSVLHSQPKSTVGTPAYIAPEVLLKKEYDGKVADVWSCGVTLYVMLVGAYPFE
DPEEPKNFRKTIHRILNVQYAIPDYVHISPECRHLISRIFVADPAKRISIPEIRNHEW
FLKNLPADLMNDNTMTTQFDESDQPGQSIEEIMQIIAEATVPPAGTQNLNHYLTG
SLDIDDDMEEDLESDLDDLDIDSSGEIVYAM

>AT4G40010.1

MERYDILRDLGSGNFGVAKLVREKANGEFYAVKYIERGLKIDEHVQREIINHRDL
KHPNIIRFKEVFVTPTHLAIVMEYAAGGELFERICNAGRFSEDEGRYYFKQLISGV
SYCHAMQICHRDLKLENTLLDGSPSSHLKICDFGYSKSSVLHSQPKSTVGTPAYV
APEVLSRKEYNGKIADVWSCGVTLYVMLVGAYPFEDPEDPRNIRNTIQRILSVHY
TIPDYVRISSECKHLLSRIFVADPDKRITVPEIEKHPWFLKGPLVVPPEEEKCDNGV
EEEEEEEEKCRQSVEEIVKIIEEARKGVNGTDNNGGLGLIDGSIDLDDIDDADIYD
DVDDDEERNGDFVCAL

>AT5G08590.1

MDKYDVVKDLGAGNFGVARLLRHKDTKELVAMKYIERGRKIDENVAREIINHR
SLKHPNIIRFKEVILTPTHLAIVMEYASGGELFDRICTAGRFSEAEARYFFQQLICG
VDYCHSLQICHRDLKLENTLLDGSPAPLLKICDFGYSKSSILHSRPKSTVGTPAYI
APEVLSRREYDGKHADVWSCGVTLYVMLVGAYPFEDPNDPKNFRKTIQRIMAV
QYKIPDYVHISQECKHLLSRIFVTNSAKRITLKEIKNHPWYLKNLPKELLESAQAA
YYKRDTSFSLQSVEDIMKIVGEARNPAPSTSAVKSSGSGADEEEEEDVEAEVEEE
EDDEDEYEKHVKEAQSCQESDKA

>AT5G63650.1

MDKYEVVKDLGAGNFGVARLLRHKETKELVAMKYIERGRKIDENVAREIINHRS
LRHPNIIRFKEVILTPTHLAIVMEYASGGELFERICNAGRFSEAEARYFFQQLICGV
DYCHSLQICHRDLKLENTLLDGSPAPLLKICDFGYSKSSLLHSRPKSTVGTPAYIA
PEVLSRREYDGKHADVWSCGVTLYVMLVGGYPFEDPDDPRNFRKTIQRIMAVQ
YKIPDYVHISQECRHLLSRIFVTNSAKRITLKEIKKHPWYLKNLPKELTEPAQAAY

YKRETPSFSLQSVEDIMKIVGEARNPAPSSNAVKGFDDDEEDVEDEVEEEEEEEE
EEEEEEEEEEDEYEKHVKEAHSCQEPPKA

>AT5G66880.1

MDRAPVTTGPLDMPIMHDSDRYDFVKDIGSGNFGVARLMRDKLTKELVAVKYI
ERGDKIDENVQREIINHRSLRHPNIVRFKEVILTPTHLAIIMEYASGGELYERICNA
GRFSEDEARFFFQQLLSGVSYCHSMQICHRDLKLENTLLDGSPAPRLKICDFGYS
KSSVLHSQPKSTVGTPAYIAPEVLLRQEYDGKIADVWSCGVTLYVMLVGAYPFE
DPEEPRDYRKTIQRILSVKYSIPDDIRISPECCHLISRIFVADPATRISIPEIKTHSWFL
KNLPADLMNESNTGSQFQEPEQPMQSLDTIMQIISEATIPAVRNRCLDDFMTDNL
DLDDDMDDFDSESEIDIDSSGEIVYAL

>VviSnRK2.6

MDRSAITVGPAMDVPIMHDSDRYELVRDIGSGNFGVARLMRDKQTGELVAVKY
IERGEKIDENVQREIINHRSLRHPNIVRFKEVILTPTHLAIVMEYASGGELFERICNA
GRFSEDEARFFFQQLVSGVSYCHAMQVCHRDLKLENTLLDGSPAPRLKICDFGY
SKSSVLHSQPKSTVGTPAYIAPEVLLKKEYDGKIADVWSCGVTLYVMLVGAYPF
EDPEEPKNFHKTIQRILKVQYSIPDYVHISPECRHLISRIFVADPATRITIAEIRNHE
WFLKNLPADLMDETKMDNQYEEPEQPMQSIDEIMRIIGEAMIPAAGTQSLNQYL
TGSLDIDDEMEEDLETDPDLDIDSSGEIVYAM

Vitis vinifera

>VviSnRK2.7a

MERYEILKDIGSGNFGVAKLVRDKWSGELYAVKYIERGQKIDEHVQREIMNHRS
LKHPNIVRFKEVLLTPTHLAIVMEYAAGGELFERICNAGRFSEDEARYFFQQLISG
VSYCHSMQICHRDLKLENTLLDGSPAPRLKICDFGYSKSSVLHSQPKSTVGTPAYI
APEVLSRKEYDGKIADVWSCGVTLYVMLVGAYPFEDPDDPRNFRKTIARILSVH
YSIPDYVRVSMECKHLLSRIFVANPEKRITIPEIQNHPWFIRNLPIEFMQGGEASAQ
TDYVNDPSQSIDDIVKIIQEARIEGPKVGGHFFGASMDLDDLDEDADLDDIEASGD
FVCAL

>VviSnRK2.7b

MERYEILKDIGSGNFGVAKLVRDKWSGELHAVKYIERGQKIDEHVQREIMNHRS
LKHPNIVRFKEVLLTPTHLAIVMEYAAGGELFERICNAGRFSEDEARYFFQQLISG
VSYCHSMQICHRDLKLENTLLDGSPAPRLKICDFGYSKSSVLHSQPKSTVGTPAYI
APEVLSRKEYDGKIADVWSCGVTLYVMLVGAYPFEDPDDPRNFRKTIARILSVH
YSIPDYVRVSMECKHLLSRIFVANPEKRITIPEIQNHPWFIRNLPIEFMQGGEASAQ
TDYVNDPSQSIDDIVKIIQEARIEGPKVGGHFFGASMDLDDLDEDADLDDIEASGD
FVCAL

>VviSnRK2.8

MERYEIIKDIGSGNFGVARLVSDKRTRELFAVKFIERGQKIDEHVQREIMNHRSL
MHPNIVRFKEVLLTPTHLAIVMEYAAGGELFGRICNAGRFSEDEARFFFQQLISGV
SYCHSMRICHRDLKLENTLLDGSTAPRVKICDFGYSKSSVLHSQPKSTVGTPAYI
APEVLSRKEYDGKLSDVWSCGVTLYVMLVGAYPFEDPDDPRNFRKTIGRILSVH
YSIPDYVRVSIECRHLLSRVFVANPDKRITIPEIKTHPWFLKNLPIEMMDGGSWQK
NDVNNPSQSVEEILSIIQEARKTTGVRKAGGPFMEGSMDLDDIDADEDIETSGDF
VCPL

>VviSnRK2.11

MEKYELVKDIGSGNFGVARLMRNKETKELVAMKYIERGQKIDENVAREIINHRS
LRHPNIIRFKEVVLTPTHLAIVMEYAAGGELFERICNAGRFSEDEARYFFQQLISG
VSYCHSMQICHRDLKLENTLLDGSPAPRLKICDFGYSKSSLLHSRPKSTVGTPAYI
APEVLSRREYDGKLADVWSCGVTLYVMLVGAYPFEDQEDPKNFRKTISRIMAV
QYKIPDYVHISQDCRHLLARIFVANPSRRVTIKDIKNHPWFLKNLPRELTEAAQAL
YYRRENPTFSLQSIEDIMKIVDEAKTPPPVSRSIGGFGWGGEEEEEEEEEKEEEVET
EEEEEDEYEKQVKEAHASGEVHVS

>VviSnRK2.12

MEKYEMVKDLGSGNFGVARLMRVKETKELVAMKFIERGHKIDENVAREIINHRS
LRHPNIIRFKEVVLTPTHLGIVMEYAAGGELFERICNAGRFSEDEARYFFQQLISG
VSYCHSMQICHRDLKLENTLLDGSPAPRLKICDFGYSKSSLLHSRPKSTVGTPAYI
APEVLSRREYDGKLADVWSCGVTLYVMLVGAYPFEDPDDPKNFRKTIGRIMAV
QYKIPEYVHISQDCKQLLSRIFVANSSRRITIKEIKNHPWFLKNLPRELTESTQANY
YQRDNLSFSLQSVEEIMKIVGEARIRCPSSRSIGGFGWGAEEGKGGEDDLDVEEE
EIEEEDEYDKRVREVHASGEFHVS

>VviSnRK2.13

MEERYDALKELGSGNFGVARLVRDKKTKELVAVKYIERGKKIDENVQREIINHR
SLRHPNIVRFKEVLLTPTHLAIVMEYAAGGELFERICNAGRFSEDEARFFFQQLIS
GVSYCHSMEICHRDLKLENTLLDGSPMPRLKICDFGYSKSALLHSQPKSAVGTPA
YIAPEVLSRKEYDGKIADVWSCGVTLYVMLVGAYPFEDPEDPRNFRKTIGRIMSV
QYSIPDYVHVSAECRQLLSRIFVANPAKRITIPEIKQHPWFLKNFPKELIEGEKTNY
GELHHDQLVQSVEEIMRIIQEARTPGEGSKVDGHSLDGALDSDDLEADLESEIDV
SGEFMAPV

>VviSnRK2.14

MDRSTLALGPIMDLPIMHDSDRYDFVRDIGSGNFGIARLMRDKLTKELVAVKYIE
RGDKIDENVQREIINHRSLRHPNIVRFKEVILTPTHLAIVMEYAAGGELFERICKA
GRFIEDEARFFFQQLISGVSYCHAMQICHRDLKLENTLLDGSPAPRLKICDFGYSK
SSVLHSQPKSTVGTPAYIAPEVLMRQEYDGKIADVWSCGVTLYVMLVGAYPFED
PDEPKDFRKTIQRILSVQYSIPSSVQISTECHHLISRIFVADPGARITIPEIKNHEWFL
KNLPADLMDEKTIGNQFEEPDQPMQSHDEIMGIISEATVPAPGVYGLNSYMTDG
LDMDDDMDLENDSDIDVDSSGEVVYAM

Malus domestica

>MdSnRk2.1

MEERYEAMKDLGSGNFGVARLVRDKKTKELVAVKYIERGKKIDENVQREIINHR
SLRHPNIVRFKELLLTPSHLAIVMEYAAGGELFERICTAGRFSEDEARFFFQQLISG
VGYCHAMEICHRDLKLENTLLDGSASPRLKICDFGYSKSAILHSQPKSTVGTPAYI
APEVLSRKEYDGKIADVWSCGVTLYVMLVGAYPFEDPEEPRNFRKTIERIMSVQ
YTIPDYVRVSADCKHLLSRVFVANPSKRISLPEIKEHPWFLKNLPKELIEVERTNY
ASVERDQPKQSIEEINKIIHEARTPGEGSKAGGKAVAGPSDSDDLEADLDSEVDCS
GDFSGHP

>MdSnRk2.2

MDRQAMTVGPAMDMPIMHDSDRYDFVRDIGSGNFGVARLMTDRQTRELVAVK
YIERGNKIDENVRREIINHRSLRHPNIIRFKEVILTPTHLAIVMEYASGGEMFERIHN
AGRFSEDEARFFFQQLISGVSYCHAMQVCHRDLKLENTLLDGSQAPRLKICDFGY
SKSSVLHSQPKSTVGTPAYIAPEVLLRQEYDGKIADVWSCGVTLYVMLVGAYPF
EDPDEPKDFRRTIQRILSVQYSIPDTVQISHECLELISRIFVPDPAARISIPEIKNHSW
FLKNLPADLMDESNQFEEPDQPMQSLDAIMHIIEEATIPAAGTLGLGSYMMDNLD
MDDDMDDLDSESELDVDSSGEIVYAI

>MdSnRk2.3

MDRQSMTVGPAMDMPIMHDSDRYDFVRDIGSGNFGVARLMTDKQTKELVAVK
YIERGSKIDENVKREIINHRSLRHPNIIRFKEVILTPTHLAIVMEYAAGGEMFERISN
AGRFSEDEARFFFQQLISGVSYCHAMQVCHRDLKLENTLLDGSPAPRLKICDFGY
SKSSVLHSQPKSTVGTPAYIAPEVLLRQEYDGKIADVWSCGVTLYVMLMGAYPF
EDPAEPKDFRKTIQRILSVQYSIPDTVQISHECLELISRIFVPDPSARITIPEIKNHPW
FLKNLPADLMDEGNQFEEPDQPMQSLDAIMQIIAEATIPAAGTLGLSPYMTDNLD
MDDDMDDLDSESELDVDSSGEIVYAI

>MdSnRk2.4

MEKYEVVKDIGSGNFGVARLMRNKETKELVAMKYIDRGHKIDENVAREIINHRS
LRHPNIIRFREVVLTPTHLGIVMEYAAGGELFERICNAGRFSEDEARYFFQQLISG
VSYCHSLQICHRDLKLENTLLDGSAAPRLKICDFGYSKSSLLHSRPKSTVGTPAYI
APEVLSRREYDGKLADVWSCGVTLYVMLVGAYPFEDQEDPKNFRKTMKKIMG
VHYKIPDHVHMSQECRHLLSRIFVANPARRITIKDIKNHPWFLKNLPRELTEAAQI
IYYRKENPTFSPQTVEDIMKIVEEAKIPPPVSRSIGGFSWGEEDGDTKEEVEGEEEE
EEDEYVKTVKEVHASGEVS

VS

>MdSnRk2.5

MEERYEAMKDLGSGNFGVARLVRDKKTKELVAVKYIERGKKIDENVQREIINHR
SLRHPNIVRFKELLLTPSHLAIVMEYAAGGELFERICTAGRFSEDEARFFFQQLISG

VSYCHAMEICHRDLKLENTLLDGSASPRLKICDFGYSKSAILHSQPKSTVGTPAYI
APEVLSRKEYDGKIADVWSCGVTLYVMLVGEYPFEDPEDPRNFRKTIEKIMSAQ
YTIPDYVRVSADCKQLLSRIFVANPSKRISLPEIKKHPWFLKNLPKELIEVERTNY
AVVERDQPKQSIEEINKIICEARTPGEGGSKAGGQAVAGPSDSGDLETDLDSEVD
VSCGFSGYP

>MdSnRk2.6

MNRSVLTVGPAMDMPIMHDSDRYELVRDIGSGNFGVARLMRDKQTGELVAVK
YIERGEKIDENVQREIINHRSLRHPNIVRFKEVILTPTHLAIVMEYASGGELFERICN
AGRFSEDEARFFFQQLISGVSYCHAMQVCHRDLKLENTLLDGSPAPRLKICDFGY
SKSSVLHSQPKSTVGTPAYIAPEVLLKKEYDGKIADVWSCGVTLYVMLVGAYPF
EDPGEPKNFRKTIHRITNVQYSIPDYVHISPECRHLISRIFVAEPEKRITIPEIRNHEW
FLKNLPADLMVENTMNNQFEEPDQPMQSLDEIMQIIAEATIPAAGANSLNQYLA
GSLDIDNMEEDLESDPDIDIDSSGEIVYAI

>MdSnRk2.7

MERYEIVKDIGSGNFGVAKLVKDKWSGELFAIKFIERGQKIDEHVQREIMNHRSL
KHPNIIRFKEVLLTPNDLAIVMEYAAGGELFEKICNAGRFSEDEARYFFQQLISGV
SYCHSMQICHRDLKLENTLLDGSSAPRLKICDFGYSKSSVLHSQPKSTVGTPAYIA
PEVLSRKEYDGKIADVWSCGVTLYVMLVGAYPFEDPEDPRNFRKTLQRILSVHY
SIPGYVRVSMECRHLLSRIFTANPEKRITIPEIKQHPWFLKNIPVEFMEENGLPNND
QNYESSSQSNEEILAIIQEGRKAAANGAKYGGRLLGSMDLDELDDADIDDIETSG
DFVCAL

>MdSnRk2.8

MERYEIVKDIGSGNFGVARLARDKLTRELFAVKFIERGHKIDEHVQREIMNHRSL
KHPNIVRFKEVLLTPTHLAIVMEYAAGGELFGRICSAGRFSEDEARFFFQQLISGV
RYCHLMQICHRDLKLENTLLDGSPAPRVKICDFGYSKSLLHSQPKSTVGTPAYIA
PEVLSKKQYDGKIADVWSCGVTLYVMIVGAYPFEDPEDPRNFRKTIGRILSVQYA
VPDYVRISIECRHLVSRIFVENPEKRITIPEIKNHPWFLKNLPVEMMEGGSWQCKD
INYPSQSLEEVMYIIQEARKPLSVPDASRPLIGSSSMDVDDLEDVEDIETSGGFVLP
NRSN

>MdSnRk2.9

MEKYELVKDIGSGNFGVARLMRNKETKELVAMKYIERGQKIDENVAREIINHRS
LRHPNIIRFKEVVLTPTHLAIVMEYAAGGELFERICNAGRFSEDEARYFFQQLISG
VSYCHSLQICHRDLKLENTLLDGSPAPRLKICDFGYSKSSLLHSRPKSTVGTPAYI
APEVLSRREYDGKLADVWSCGVTLYVMLVGAYPFEDQEDPKNFRKTINKIMSV
QYKIPDYVHISQDCRHLLSRIFVANSGRRITIKEIKNHPWFLKNLPRELTEAAQTV
YYRKENPTFSLQSIEAIMKIVEEAKNPPPVSRSIGGFGWGEEDGDTKEDVEGEEEE
EEDEYDKTVKEVHASGEVRFS

Capsicum annuum

>CaSnRK2.1

MEEKYELLKELGAGNFGVARLVKDQKTKELFAVKYIERGKKIDENVQREIINHR
SLRHPNIVRFKEARFFFQQLTSGVSYCHAMEICHRDLKLENTLLDGSPSPRLKICD
FGYSKSGLLHSQPKSTVGTPAYIAPEIADVWSCGVTLYVMLVGAYPFEDPEDPK
NFRKTIGRIMSAQYSIPDYVRISADCKNLLSRIFVANPSKRITIPEIKKHPWFLKNLP
KELMDGQQAKYEEASEQLQQSVEEIMKMIQEAKIPGVVSKPEGKDPAGTTVQDD
TEEDLESEVDSSSDFAVYV

>CaSnRK2.2

MDRGPGPCMDMPIMHDSDRYDFVKDIGSGNFGVARLMTDKQTKELVAVKYIER
GDKQVCHRDLKLENTLLDGSPAPRLKICDFGYSKSALLHSQPKSTVGTPAYIAPE
VLLRQEYDGKIADVWSCGVTLYVMLVGAYPFEDPDEPKDFRKTINRILSVQYSM
PENIQISEECRHLISRIFVGDPAQRITMPEIRNHVWFLKNLPADLMDDKMISDQFE
EPDQPMQSIDTIMQIISEATVPPVGLYNLEMMDDDMDDLDSDPDLDIDSSGEIIYA
M

>CaSnRK2.3

MERYEIVKELGSGNFGVAKLVSDKKTKELFAVKFIERGQKYISGLSLQQICHRDL
KLENTLLDGSAAPRVKICDFGYSKSSVFHSQPKSTVGTPAYVAPEILTRKEYDGK
LADVWSCGVTLYVMLVGAYPFQDPSDAKNFTKTIAKILGVRYSIPEQVRISLECR
HLLSRIFVADPEKRITIPEIKMHPWFLKNLPVELMEGGSYQCVDVNNPSQSMEEV
LAIIHEARVPLQVPKGGTYSCGGSMELDELDEADIEDVIETSADFTGLM

>CaSnRK2.4

MERYEIQKDIGSGNFGVAKLVKDKWSGELFTVKYIQRGKKSAALHSQPKSTVGT
PAYIAPEVLSRKEYDGKIADVWSCGVTLYVMLVGAYPFEDPDDPRNFKKTITRIL
SIQYSIPYYVRVSKECNHLLSRIFVADPEKRITIEEIKKHPWFLKHLPKEFMEGEEA
SLVQVNGGEKPLQSIEEALAIIQEARKPEEGSKASDYFVNSSISMDLDDFDTDADL
DDEIDTSGDFVCAL

>CaSnRK2.5

MEERYEPLKELGCGNFGVARLVRDKKTKELVAVKYIERGRKIDENVQREIINHRS
LRHPNIVRFKEARFFFQQLISGVSYCHSMEICHRDLKLENTLLDGSPIPRVKICDFG
YSKSGLLHSQPKSTVGTPAYIAPEVLSRKEYDGKVADVWSCGVTLYVMLVGAY
PFEDPEDPRNFRKTIGRIMSAQFSIPDYIRISAGCMNLLSRIFVANPSKRISIPEIKKH
PWFLKHLPKNLMDGRNVNYGEASGQQLQRVEDIMRIIQEAKFLGEGSKYGQAP
AEATIDPDDSEGDLESEIETSGDYIIHF

>CaSnRK2.6

MERYELVKDIGSGNFGVARLMRHKETKELVAMKYIERGHKIDENVAREIINHKS
LRHPNIIRFKEARALIPSARYFFQQLISGVHYCHNLQICHRDLKLENTLLDGSAAP
RLKICDFGYSKSSLLHSRPKSTVGTPAYIAPEVLSRREYDGKLADVWSCGVTLYV

MLVGAYPFEDQEDPKNFRKTIQRIMAVQYKIPDYVHISQDCRHLLSRIFVANPAR
RITIKEIKSHPWFLKNLPRELTEAAQAAYYRKENPTFSLQSVEEIMKIVEEAKAPPP
ASRSVSGFGWGGEEEEEEKEGDAEEEVDEEEEEEDEYDKQVKQAHQSLGDVRLS

>CaSnRK2.7

MDRTAVTVGPGMDVPIMHDSDRYELVRDIGSGNFGVARLMRDRQTNELVAVK
YIERGEKAISCKFQARFFFQQLISGVSYCHAMQVCHRDLKLENTLLDGSPAPRLKI
CDFGYSKSSVLHSQPKSTVGTPAYIAPEVLLKKEYDGKIADVWSCGVTLYVMLV
GAYPFEDPEEPKNFRKTIQRILNVQYSIPDYVHISPECRHLISRIFVADPAKRITIPEI
KNHEWFLRNLPADLMDNTTNNQFEEPDQRMQSIDEIMQIITEATIPSVGTVLFYIL
IGS

>CaSnRK2.8

MERYEILKDIGSGNFGVAKLVKDKLTNELYAVKYIERGKKVCFASSLSLIQFIVFP
LGVRYPLQRSKQICHRDLKLENTLLDGSSTPRLKICDFGYSKAIILNSSSVLHSQPK
STVGTPAYIAPEVLSRKEYDGKLADVWSCGVTLYVMLVGAYPFEDPEDPRNFRK
TLTRILTVQYSIPYYVRVSKECKHLLSQIFVADPGKRITIEEIKNHPWFLKDLPKEY
MEGEEASLETINGENEPSQSIDEVLAIIEEARKPGEGPQACDLLVKGSSISMDDLD
EDDSDIDDDTDDEIETSGDFVCEL

>CaSnRK2.9

MQNYEVVKELGSGNFGVARLMRHKETKQLVAMKYIERGRKQICHRDLKLENTL
LDGSPAPRLKICDFGYSKSSVLHSRPKSTVGTPAYIAPEVLSRREYDGKSADVWS
CGVTLYVMLVGAYPFEDLDDPRNFRKTISRIMAVQYKIPDYVHISHDCKHLLSRI
FVASPARRITLKEIKNHPWFLKSLPKELTESAQAVYYKRDNPTFSLQSIEEIMKIVT
EARSPPPTSRPVPGFGWGTEEEEDGETKGDEEEEEEEEDEYEKQVKQVHASGEFH
ITHDDA

Solanum lycopersicum

>SlSnRK2.1

MEKYELVKDIGSGNFGVARLMRNKETKELVAMKYIERGHKIDENVAREIINHKS
LRHPNIIRFKEVVLTPTHLAIVMEYAAGGELFERICNAGRFSEDEARYFFQQLISG
VHYCHNMQICHRDLKLENTLLDGSAAPRLKICDFGYSKSSLLHSRPKSTVGTPAY
IAPEVLSRREYDGKLADVWSCGVTLYVMLVGAYPFEDQEDPKNFRKTIQRIMAV
QYKIPDYVHISQDCRHLLSRIFVANSARRITIKEIKSHPWFLKNLPRELTEAAQAA
YYRKENPTFSLQSVEEIMKIVEEAKTPPPVSRSVSGFGWGGEEEEEEKEGDVEEE
VEEEDDDEEEEDEYDKQVKQAHQSLGEVRLT

>SlSnRK2.2

MDRTAVTVGPGMDVPIMHDSDRYELVRDIGAGNFGVARLMRDRQTNELVAVK
YIERGEKIDENVKREIINHRSLRHPNIVRFKEVILTPTHLAIVMEFASGGELFERICN
AGRFSEDEARFFFQQLISGVSYCHAMQVCHRDLKLENTLLDGSPAPRLKICDFGY

SKSSVLHSQPKSTVGTPAYIAPEVLLKKEYDGKIADVWSCGVTLYVMLVGAYPF
EDPEEPKNFRKTIQRILNVQYSIPDYVHISPECRHLISRIFVADPAKRISIPEIKNHE
WFLKNLPADLMDNTTNNQFEEPDQRMQSIDEIMQIITEATIPAAGTNSLNHYLTG
SLDIDDDMEEDLESDPDLDIDSSGEIVYAM

>SlSnRK2.3

MDRGPGPCMDMPIMHDSDRYDFVRDIGSGNFGVARLMTDKQTKELVAVKYIER
GDKIDENVQREIINHRSLRHPNIIRFKEVILTPTHLAIVMEYASGGELFERISNAGRF
NEDEARFFFQQLISGVSYCHSMQVCHRDLKLENTLLDGSPAPRLKICDFGYSKSA
LLHSQPKSTVGTPAYIAPEVLLRKEYDGKIADVWSCGVTLYVMLVGAYPFEDPD
EPKDFRKTINRILSVQYSVPENIQISEECRHLISRIFVGDPAQRITMPEIRNHVWFLK
NLPADLIDDRMISDQFEEPDQPMQSIDTIMQIISEATVPPIGLYNLEMMDDDMDDL
DSEPDLDIDSSGEIIYAM

>SlSnRK2.4

MERYEIQKDIGSGNFGVAKLVKDKWSGELFAVKYIERGKKIDEHVQREIMNHRS
LRHPNIIRFKEVFLTPAHLAIVMEYASGGELFERICSAGRFSEDEARFFFQQLISGV
SYCHSMQICHRDLKLENTLLDGSSTQCLKICDFGYSKSAALHSQPKSTVGTPAYI
APEVLSRKEYDGKIADVWSCGVTLYVMLVGAYPFEDPDDPRNFKKTITRILSVQ
YSIPYYVRVSKECNHLLSRIFVADPEKRITIEEIKKHPWFLKNLPKEFMKKGEEAS
LVQMNSEEKPLQSIEEALAIIQEARKPGEGSKASDYFVNSSISMDLDDFDTDADL
DDEIDTSGDFVCAL

>SlSnRK2.5

MMERYEIVKELGSGNFGVAKLVCDKNTKELFAVKFIERGQKIDEHVQREIMNHR
SLKHPNIVRFKEVLLTPTHLAIVMEYAAGGELFARICNAGRFNEDEARFFFQQLIS
GVSYCHFMQICHRDLKLENTLLDGSAAPRVKICDFGYSKSSVFHSQPKSTVGTPA
YVAPEVLTRKEYDGKLADVWSCGVTLYVMLVGAYPFQDSSDPKNIKKTISKILT
ARYSIPEQIQISLECRHLITRIFVADPEKRITIPEIKMHPWFLKNLPVELMEGGSYQC
VDVNNPSQSMEEVLAIIQEARVPLQVGTHSFGGSMELDELDEADIEDVIETSADF
AGLL

>SlSnRK2.6

MQNYEVVKELGSGNFGVARLMRHKETKQLVAMKYIERGRKIDENVAREIINHRS
LRHPNIIRFKEVLLTSTHLGIVMEYAAGGELFDRICQAGRFSEPEARYFFQQLISGV
HYCHNMQICHRDLKLENTLLDGSPAPRLKICDFGYSKSSVLHSRPKSTVGTPAYI
APEVLSRREYDGKSADVWSCGVTLYVMLVGGYPFEDVDDPKNFRKTISRIMGV
QYKIPDYVHISHDCKHLLSRIFVASPARRITLKEIKNHPWFLKSLPKELTETAQAV
YYKRDNPTFSIQSIEEIMKIVSEARNPAPPSRPVPHFGWGTEEEEEVVEEEEEEEEE
EDEYDKQVKQVHASGEFHIIHDDA

>SlSnRK2.7

MEEKYELLKELGAGNFGVARLVKDKKTKELLAVKYIERGKKIDENVQREIINHR
SLRHPNIVRFKEVLVTPSHLAIVMEYAAGGELFGRICSAGRFSEDEARFFFQQLIS
GVSYCHTMEICHRDLKLENTLLDGSPSPRLKICDFGYSKSGLLHSQPKSTVGTPA
YIAPEVLSRKEYDGKIADVWSCGVTLYVMLVGAYPFEDPEDPKNFRKTIGRIMS
AQHSIPDYVRVTPDCRNLLSRIFVANPSKRITIPEIKKHPWFLKNLPKELMDVEHA
RFEEASEQLQQSVEEIMKMIQEAKVPGVVSKSEGKDPAGTAEQDDLEEDLESEID
SSNDFAVYV

>SlSnRK2.8

KDIGSGNFGVAKLVKDKLTNELYAVKYIERGKKIDEHVQREIMNHRSLRHPNIIR
FKEVFLTPTHLAIVMEYAAGGELFERICNAGRFSEDEARFFFQQLISGVSYCHSM
QICHRDLKLENTLLDGSSKPRLKICDFGYSKSSLLHSQPKSTVGTPAYIAPEVLSR
KEYDGKLADVWSCGVTLYVMLVGAYPFEDPEDPRNFRKTLTRILSVQYSIPYYV
RVSKECKLLLSQIFVADPSKRITIEEIKKQPWFLKDLPIEYMEGEDASLQMKEENE
PTQSIDEVLAIIQEAKKPGEGPKGCDLFVNGSSSIDFEDDDSDIDDDTDDEIETSGE
FVCAL

Solanum tuberosum

>StSnRK2.1

MERYEIVKELGSGNFGVAKLVCDKNTKELFAVKFIERGQKIDEHVQREIMNHRS
LKHPNIVRFKEVLLTPTHLAIVMEYAAGGELFARICNAGRFNEDEARFFFQQLISG
VSYCHFMQICHRDLKLENTLLDGSAAPRVKICDFGYSKSSVFHSQPKSTVGTPAY
VAPEVLTRKEYDGELADVWSCGVTLYVMLVGAYPFQDSSDPKNFTKTISKILTA
RYSIPEQIQISLECRHLIARIFVADPEKRITIPEIKMHPWFLKNLPVELMEGGSYQC
ADVNNPSQSMEEVLAIIQEARVPLLVGAHSYGGSMELDELDEADIEDVIETSADF
AGLL

>StSnRK2.2

MEEKYELLKELGAGNFGVARLVKDKKTKELLAVKYIERGKKIDENVQREIINHR
SLRHPNIVRFKEVLVTPSHLAIVMEYAAGGELFGRICSAGRFSGDEARFFFQQLIS
GVSYCHTMEICHRDLKLENTLLDGSPSPRLKICDFGYSKSGLLHSQPKSTVGTPA
YIAPEVLSRKEYDGKIADVWSCGVTLYVMLVGAYPFEDPEDPKNFRKTIGRIMS
AQHSIPDYVRITPDCKNLLSRIFVANPSKRITIPETKKHPWFLKNLPKELMDVEHA
KFEESSEQLQQSVEEIMKMIQEAKIPGVVSKSEGKDPAGTAEQDDLEEDLESEIDS
SNDFAVYV

>StSnRK2.3

MDRTAVTVGPGMDVPIMHDSDRYELVRDIGAGNFGVARLMRDRQTNELVAVK
YIERGEKIDENVKREIINHRSLRHPNIVRFKEVILTPTHLAIVMEFASGGELFERICN
AGRFSEDEARFFFQQLISGVSYCHAMQVCHRDLKLENTLLDGSPAPRLKICDFGY
SKSSVLHSQPKSTVGTPAYIAPEVLLKKEYDGKIADVWSCGVTLYVMLVGAYPF
EDPEEPKNFRKTIQRILNVQYSIPDYVHISPECRHLISRIFVADPAKRISIPEIKNHE

WFLKNLPADLMDNTTNNQFEEPDQRMQSIDEIMQIITEATIPAAGTNSLNHYLTG
SLDIDDDIEEDLESDPDLDIDSGGEIVYAM

>StSnRK2.4

MEKYELVKDIGSGNFGVARLMRNKETKELVAMKYIERGHKIDENVAREIINHKS
LRHPNIIRFKEVVLTPTHLAIVMEYAAGGELFERICNAGRFSEDEARYFFQQLISG
VHYCHNMQICHRDLKLENTLLDGSAAPRLKICDFGYSKSSLLHSRPKSTVGTPAY
IAPEVLSRREYDGKLADVWSCGVTLYVMLVGAYPFEDQEDPKNFRKTIQRIMAV
QYKIPDYVHISQDCRHLLSRIFVANSARRITIKEIKSHPWFLKNLPRELTEAAQAA
YYRKENPTFSLQSVEEIMKIVEEAKTPPPVSRSVSGFGWGGEEEEEEKEGDVEEE
VEEEEDEEEEDEYDKQVKQAHQSLGEVRLT

>StSnRK2.5

MERYEIQKDIGSGNFGVAKLVKDKWSGELFAVKYIERGKKIDEHVQREIMNHRS
LRHPNIIRFKEVFLTPAHLAIVMEYASGGELFERICNAGRFSEDEARFFFQQLISGV
SYCHSMQICHRDLKLENTLLDGSSTQCLKICDFGYSKSAALHSQPKSTVGTPAYI
APEVLSRKEYDGKIADVWSCGVTLYVMLVGAYPFEDPDDPRNFKKTITRILSVQ
YSIPYYVRVSKKCNHLLSRIFVADPEKRITIEEIKKHPWFLKNLPKEFMKGEEASL
VQMNSEEKPLQSIEEALAIIQEARKPGEGSKASDYFVHSSISMNLDDFDTDADLD
DEIDTSGDFVCAL

>StSnRK2.6

MQNYEVVKELGSGNFGVARLMRHKETKQLVAMKYIERGRKIDENVAREIINHRS
LRHPSIIRFKEVLLTSTHLGIVMEYAAGGELFDRICQAGRFSEPEARYFFQQLISGV
HYCHNMQICHRDLKLENTLLDGSPAPRLKICDFGYSKSSVLHSRPKSTVGTPAYI
APEVLSRREYDGKSADVWSCGVTLYVMLVGGYPFEDVDDPKNFRKTISRIMGV
QYKIPDYVHISHDCKHLLSRIFVASPARRITLKEIKNHPWFLKSLPKELTESAQAV
YYKRDNPTFSLQSIEEIMKIVSEARNPPPPSRPVPHFGWGTEEEEEDGETKEEDEE
EDEEDEYEKQVKQVHASGEFHITHDDA

>StSnRK2.7

MIEGYEFVKDLGCGNFGVAKLVRDYKTKELFAVKFFERGQKIDEHVQREIMNHR
SLSHPNIIRFKEVLLTPTHLAIVMEYAAGGELFQRICKAGRFNENEARFFFQQLISG
VSYCHFMQICHRDLKLENTLLDGSTAPRVKICDFGYSKSSVFHSQPKSTVGTPAY
VAPEILSKKEYDGKVADVWSCGVTLYVMLVGAYPFEDPTDPKNIRKTISRIFSVQ
YSIPQNVQISVECQHLLSRIFVADPEKRITIPEIKKHPWFVKNLPVEFMEEGRNECI
DVNNPKQSMEEVLAIIQEARIALQVLASSSQGIMEEFDELDDADIQDIETSGDFLC
YL

>StSnRK2.8

MERYEILKDIGSGNFGVAKLVKDKLTYELYAVKYIERGKKIDEHVQREIMNHRS
LKHPNIIRFKEVFLTPTHLAIVMEYAAGGELFERICNAGRFSEDEARFFFQQLISGV
SYCHSMQICHRDLKLENTLLDDSSNPRLKICDFGYSKSSVLHSQPKSTVGTPAYIA

PEVLLRKEYDGKLADVWSCGVTLYVMLVGAYPFEDPEDPRNFRKTLTRILSVQY
SIPYYVRVSKECRLLLSQIFVADPSKRITIEEIKKHPWFLKDLPIEYMEGEDASLQM
KEENEPTQSIDEVLAIIQEAKKPGEGPKGCDLLVKGISSSIDLDDDDDDDSDIDDD
TDDEIETSGEFVCAL

Oryza sativa

>OsSAPK1

MERYEVMRDIGSGNFGVAKLVRDVATNHLFAVKFIERGLKIDEHVQREIMNHRS
LKHPNIIRFKEVVLTPTHLAIVMEYAAGGELFERICNAGRFSEDEARFFFQQLISG
VSYCHSMQVCHRDLKLENTLLDGSVTPRLKICDFGYSKSSVLHSQPKSTVGTPA
YIAPEVLSRKEYDGKVADVWSCGVTLYVMLVGAYPFEDPDDPRNFRKTITRILS
VQYSIPDYVRVSADCRHLLSRIFVGNPEQRITIPEIKNHPWFLKNLPIEMTDEYQRS
MQLADMNTPSQSLEEVMAIIQEARKPGDAMKLAGAGQVACLGSMDLDDIDDID
DIDIENSGDFVCAL

>OsSAPK2

MERYEVIKDIGSGNFGVAKLVRDVRTKELFAVKFIERGQKIDENVQREIMNHRSL
RHPNIVRFKEVVLTPTHLAIVMEYAAGGELFERICSAGRFSEDEARFFFQQLISGV
SYCHSMQICHRDLKLENTLLDGSIAPRLKICDFGYSKSSLLHSQPKSTVGTPAYIA
PEVLARKEYDGKVADVWSCGVTLYVMLVGAYPFEDPDEPRNFRKTITRILSVQY
MVPDYVRVSMECRHLLSRIFVANPEQRITIPEIKNHPWFLKNLPIEMTDEYQMSV
QMNDINTPSQGLEEIMAIIQEARKPGDGSKFSGQIPGLGSMELDDVDTDDIDVEDS
GDFVCAL

>OsSAPK3

MEERYEALKELGAGNFGVARLVRDKRSKELVAVKYIERGKKIDENVQREIINHR
SLRHPNIIRFKEVCLTPTHLAIVMEYAAGGELFEQICTAGRFSEDEARYFFQQLISG
VSYCHSLEICHRDLKLENTLLDGSPTPRVKICDFGYSKSALLHSKPKSTVGTPAYI
APEVLSREEYDGKVADVWSCGVTLYVMLVGSYPFEDPGDPRNFRKTISRILGVQ
YSIPDYVRVSSDCRRLLSQIFVADPSKRITIPEIKKHTWFLKNLPKEISEREKADYK
DTDAAPPTQAVEEIMRIIQEAKVPGDMAAADPALLAELAELKSDDEEEAADEYD
TY

>OsSAPK4

MEKYEAVRDIGSGNFGVARLMRNRETRELVAVKCIERGHRIDENVYREIINHRSL
RHPNIIRFKEVILTPTHLMIVMEFAAGGELFDRICDRGRFSEDEARYFFQQLICGVS
YCHHMQICHRDLKLENVLLDGSPAPRLKICDFGYSKSSVLHSRPKSAVGTPAYIA
PEVLSRREYDGKLADVWSCGVTLYVMLVGAYPFEDQDDPKNIRKTIQRIMSVQY
KIPDYVHISAECKQLIARIFVNNPLRRITMKEIKSHPWFLKNLPRELTETAQAMYY
RRDNSVPSFSDQTSEEIMKIVQEARTMPKSSRTGYWSDAGSDEEEKEEEERPEEN
EEEEEDEYDKRVKEVHASGELRMSSLRI

>OsSAPK5

MEKYEPVREIGAGNFGVAKLMRNKETRELVAMKFIERGNRIDENVFREIVNHRS
LRHPNIIRFKEVVVTGRHLAIVMEYAAGGELFERICEAGRFHEDEARYFFQQLVC
GVSYCHAMQICHRDLKLENTLLDGSPAPRLKICDFGYSKSSLLHSRPKSTVGTPA
YIAPEVLSRREYDGKLADVWSCGVTLYVMLVGAYPFEDPKDPKNFRKTISRIMS
VQYKIPEYVHVSQPCRHLLSRIFVANPYKRISMGEIKSHPWFLKNLPRELKEEAQ
AVYYNRRGADHAASSASSAAAAAAFSPQSVEDIMRIVQEAQTVPKPDKPVSGYG
WGTDDDDDDQQPAEEEDEEDDYDRTVREVHASVDLDMSNLQIS

>OsSAPK6

MEKYELLKDIGSGNFGVARLMRNRETKELVAMKYIPRGLKIDENVAREIINHRSL
RHPNIIRFKEVVLTPTHLAIVMEYAAGGELFDRICSAGRFSEDESRYFFQQLICGVS
YCHFMQICHRDLKLENTLLDGSPAPRLKICDFGYSKSSLLHSKPKSTVGTPAYIAP
EVLSRREYDGKMADVWSCGVTLYVMLVGAYPFEDPDDPKNFRKTIGRIVSIQYK
IPEYVHISQDCRQLLSRIFVANPAKRITIREIRNHPWFMKNLPRELTEAAQAKYYK
KDNSARTFSDQTVDEIMKIVQEAKTPPPSSTPVAGFGWTEEEEQEDGKNPDDDE
GDRDEEEGEEGDSEDEYTKQVKQAHASCDLQKS

>OsSAPK7

MERYELLKDIGAGNFGVARLMRNKETKELVAMKYIPRGLKIDENVAREIINHRSL
RHPNIIRFKEVVVTPTHLAIVMEYAAGGELFDRICNAGRFSEDEARYFFQQLICGV
SYCHFMQICHRDLKLENTLLDGSPAPRLKICDFGYSKSSLLHSKPKSTVGTPAYIA
PEVLSRREYDGKTADVWSCGVTLYVMLVGAYPFEDPDDPKNFRKTIGRIMSIQY
KIPEYVHVSQDCRQLLSRIFVANPAKRITIREIRNHPWFLKNLPRELTEAAQAMYY
KKDNSAPTYSVQSVEEIMKIVEEARTPPRSSTPVAGFGWQEEDEQEDNSKKPEEE
QEEEEDAEDEYDKQVKQVHASGEFQLS

>OsSAPK8

MAAAGAGAGAPDRAALTVGPGMDMPIMHDSDRYELVRDIGSGNFGVARLMRD
RRTMELVAVKYIERGEKIDDNVQREIINHRSLKHPNIIRFKEVILTPTHLAIVMEYA
SGGELFERICKNVRFSEDEARYFFQQLISGVSYCHSMQVCHRDLKLENTLLDGSP
APRLKICDFGYSKSSVLHSQPKSTVGTPAYIAPEVLLKKEYDGKTADVWSCGVTL
YVMVVGAYPFEDPEEPKNFRKTIQRILNVQYSIPENVDISPECRHLISRIFVGDPSL
RITIPEIRSHGWFLKNLPADLMDDDSMSSQYEEPDQPMQTMDQIMQILTEATIPPA
CSRINHILTDGLDLDDDMDDLDSDSDIDVDSSGEIVYAM

>OsSAPK9

MERAAAGPLGMEMPIMHDGDRYELVKEIGSGNFGVARLMRNRASGDLVAVKYI
DRGEKIDENVQREIINHRSLRHPNIIRFKEVILTPTHLAIVMEYASGGELFERICSAG
RFSEDEARFFFQQLISGVSYCHSMQVCHRDLKLENTLLDGSTAPRLKICDFGYSK
SSVLHSQPKSTVGTPAYIAPEVLLKKEYDGKIADVWSCGVTLYVMLVGAYPFED
PEDPKNFRKTIQKILGVQYSIPDYVHISPECRDLITRIFVGNPASRITMPEIKNHPWF
MKNIPADLMDDGMVSNQYEEPDQPMQNMNEIMQILAEATIPAAGTSGINQFLTD
SLDLDDDMEDMDSDLDLDIESSGEIVYAM

>OsSAPK10

MDRAALTVGPGMDMPIMHDGDRYELVRDIGSGNFGVARLMRSRADGQLVAVK
YIERGDKIDENVQREIINHRSLRHPNIIRFKEVILTPTHLAIVMEYASGGELFERICN
AGRFSEDEARFFFQQLISGVSYCHSMQVCHRDLKLENTLLDGSTAPRLKICDFGY
SKSSVLHSQPKSTVGTPAYIAPEVLLKKEYDGKIADVWSCGVTLYVMLVGAYPF
EDPDEPKNFRKTIQRILGVQYSIPDYVHISPECRDLIARIFVANPATRISIPEIRNHP
WFLKNLPADLMDDSKMSSQYEEPEQPMQSMDEIMQILAEATIPAAGSGGINQFL
NDGLDLDDDMEDLDSDPDLDVESSGEIVYAM

Zea mays

>ZmSnRK2.1

MERYEVIRDIGSGNFGVTKLVRDVRTKELFAVKFIERGQKIDEHVQREIMNHRSL
KHPNIIRFKEVVLTPTHLAIVMEYAAGGELFERICSAGRFSEDEARFFFQQLISGVS
YCHSMQVCHRDLKLGNTLLDGSVAPRLKICDFGYSKSSVLHSQPKSTVGTPAYI
APEVLSRKEYDGKVADVWSCGVTLYVMLVGAYPFEDPDDPKNFRKTITRILSVQ
YSIPDYVRVTMECGHLLSRIFVGNPERRITIPEIKKHPWFLKNLPIEMTDEYQQSM
QLADMNTPGQSLEEVMAIIQEARKPGDAMNLAGQLPCLGSVDFDDIDFDDIDDID
TENSGDFVCAV

>ZmSnRK2.2

MERYEVIKDIGSGNFGVAKLVRDVRTKELFAVKFIERGMKIDENVQREIMNHRSL
RHPNIVKFKEVVLTPTHLAIVMEYAAGGELFERICNAGRFSEDEARFFFQQLISGV
SYCHSMQICHRDLKLENTLLDDSIAPRLKICDFGYSKSSVLHSQPKSTVGTPAYIA
PEVLARKEYDGKVADVWSCGVTLYVMLVGAYPFEDPDEPKNFRKTLTRIISVQY
AVPDFVRVSMECRHLLSRIFVAKPEQRITIPEIKNHPWFLKNLPIEMTDEYQMNLQ
LVDMNVPSQSLEEIMSIILEARKPGDGLKHAGQLPGLGSMELDDTDVDDIDVEDS
GDFVCAL

>ZmSnRK2.3

MEERYEALKELGAGNFGVARLVRDKRTKELVAVKYIERGKKIDENVQREIINHQ
SLRHPNIVRFKEVCLTPTHLAIVMEYAAGGELFEKICSAGRFSEDESRYFFQQLIS
GVSYCHSMEICHRDLKLENTLLDGSPTPRVKICDFGYSKSALLHSKPKSTVGTPA
YIAPEVLSRKEYDGKVADVWSCGVTLYVMLVGSYPFEDPEDPRNFRKTISRILGV
QYSIPDYVRVSSDCRRLLSQIFVADPSKRITIPEIKHHPWFLKNLPREISEREKANY
KDADAAEPAQAVDEIMRIVEEAKTPGDMSKVVDPALLAEMAELESDEEEADAD
DTY

>ZmSnRK2.4

MDKYEAVRDIGSGNFGVARLMRNRETRELVAVKCIERGHRIDENVYREIINHRSL
RHPNIIRFKEVILTPTHLMIVLEFAAGGELFDRICDRGRFSEDEARYFFQQLICGVS
YCHYMQICHRDLKLENVLLDGSPAPRLKICDFGYSKSSVLHSRPKPAVGTPAYIA
PEVLSRREYDGKLADVWSCGVTLYVMLVGAYPFEDPDDPKNIRKTIQQIMQVQ

YKIPDHVHISTECQQLIARIFVANPMRRITMKEIKSHPWFLKNLPRELTETAQGMY
YRRDNRVPSYSDQTSEEVMKIVQDARTMPKSSRSGYGWSSEYSDEEEEKEEEHR
PEEHEEEEDEYDRRVKEVHASGELRMDALHI

>ZmSnRK2.5

MDKYEPVREIGAGNFGVAKLMRNKDTRELVAMKFIERGNRIDENVFREIVNHRS
LRHPNIIRFKEVVLTPTHLAIVMEYAAGGELFERICDAGRFHEDEARYFFQQLVC
GVSFCHAMNICHRDLKLENTLLDGSPAPRLKICDFGYSKSSVLHSRPKSTVGTPA
YIAPEVLSRREYDGKHADVWSCGVTLYVMLVGAYPFEDPKDPKNFRKTIQRIMS
VQYKIPEYVHVSHNCRHLLSRIFVQNPYKRITMSEIKTHPWYLKNLPRELKEEAQ
AAYYYSRQGDSSSSSSNNATAAPAYSSQSVEEILRIVQEAQTVPKPPSRPDEAEQQ
EEDDDHDDDDDYDRTVRQVHASGEFDMIMSRLQI

>ZmSnRK2.6

MEKYELLKDIGSGNFGVARLMRNKDTKELVAMKYIPRGLKIDENVAREIINHRSL
RHPNIIRFKEVVLTPTHLAIVMEYAAGGELFDRICSAGRFSEDEARYFFQQLICGV
SYCHFMQICHRDLKLENTLLDGSPAPRLKICDFGCSKSSLLHSKPKSRLELRRTLL
REVLSRREYDGKMADVWSCGVTLYVMLVGAYPFEDPDDPKNFRKAIGRIVSIQY
QIPEYVHISQDCRQLLARIFVANPAKRITIREIRNHPWFLKNLPRELTEAAQAKYY
KKDNGAPTFSDQTVEEIMKIVEEARTPPQSPAPVAGFGWAEEEEQEDGKKPEDD
EQDGEDEQYDGEDEYDKQVKQVHASGDFRHLIK

>ZmSnRK2.7

MEKHELLKDIGAGNFGVARLMRNKETKELVAMKYIPRGQKIDENVAREIINHRS
LRHPNIIRFKEVVLTPTHLAIVMEYAAGGELFDRICNAGRFSEDEARYFFQQLICG
VSYCHSMQICHRDLKLENTLLDGSPAPRLKICDFGYSKSSLLHSKPKSTVGTPAYI
APEVLSRREYDGKTADVWSCGVTLYVMLVGGYPFEDPDDPKNFRKTIGRIMSIQ
YKIPEYVHVSQDCKELLSRIFVANSAKRITIREIRNHPWFLKNLPRELTEAAQAMY
YKKDNSAPTYSVQSVEEIMKIVEKARTPPPSSTPVAGFGWAEEDEQEDSKDPDEE
HEDGEDEYEKQVKQVHASGEFHLSC

>ZmSnRK2.8

MAGPAPDRAALTVGPGMDMPIMHDSDRYELVRDIGSGNFGVARLMRDRRTSEL
VAVKYIERGEKIDENVQREIINHRSLKHPNIIRFKEVILTPTHLAIVMEYASGGELF
ERICKNVRFSEDEARYFFQQLISGQVCHRDLKLENTLLDGSDAPRLKICDFGYSKS
SVLHSQPKSTVGTPAYIAPEVLLKKEYDGKIADVWSCGVTLYVMVVGAYPFEDP
EEPKNFRKTIQRILNVQYAIPDNVNISPECRHLISRIFVGDPATRITIPEIRNHSWFL
KNLPADLMDDDSMSNQYEEPDQPMQTMDQIMQILTEATIPPACSRSINVLADGL
DMDDDMDDLDSDSDLDIDSSGEIVYAM

>ZmSnRK2.10

MDRAALTVGPGMDMPIMHDGDRYELVRDIGSGNFGVARLMRNRADGQLVAVK
YIERGEKIDENVQREIINHRSLRHPNIIRFKEVILTPTHLAIVMEYASGGELFERICN

AGRFSEDEARFFFQQLISGVSYCHSMQVCHRDLKLENTLLDGSTAPRLKICDFGY
SKSSVLHSQPKSTVGTPAYIAPEVLLKKEYDGKVADVWSCGVTLYVMLVGAYP
FEDPDEPKNFRKTIQRILGVQYSIPDYVHISPECQNLVSRIFVADPATRITIPEIRNH
PWFLKNLPADLMDDSTMSKQYEEPEQPMQSMDEIMQILAEATIPAAGPHGLNQF
LNDGLDLDDDMDDLDSDTDLDLESSGEIVYAI

>ZmSnRK2.11

MDKYEAVRDIGSGNFGVARLMRNRETRELVAVKLIERGHRIDENVYREIVNHRS
LRHPNIIQFIEVILTPTHLAIVMEYAAGGELFDRIVDRGRFSEDETRYFFQQLICGV
SYCHHMQICHRDLKLENVLLDGSPAPRLKICDFGYSKSSVLHSRPKSAVGTPAYI
APEVLSRREYDGKLADVWSCGVTLFVMLVGAYPFEDQDDPKNIRKTIQRIAAIQ
YNIPDNIRISDDCRQLISRIFVSNPLRRITMREIKSHPWFLKNLPRELTEAVQLSYFR
RGNSVSAFSDQTTEEIMKIVKEARTLPKSSRSGYGYSEECSDEEEKEVESEPKEEE
EEAECDKTVREVRESGELDMTSLHI
