# Supplementary material for: Genome-Wide Identification and Expression Analysis of SnRK2 Gene Family in Dormant Vegetative Buds of Liriodendron chinense in Response to Abscisic Acid, Chilling, and Photoperiod
Source: Genes (Basel). 2022 Jul 22;13(8):1305. doi: 10.3390/genes13081305 (PMC9331246; doi:10.3390/genes13081305)
Supplement: Supplementary file 1 [file genes-13-01305-s001.zip › Table S5.pdf]

Table S5. Information of CDS sequence of SnRK2 family in *Liriodendron chinense*.

>Lchi13910

ATGGATCGATCGGCGGTGACGATCGGTCCGGGGATGGACATGCCGATAATGCACGACGGTGATCGGTACGAGCT
CGTGAGAGACATCGGGTCGGGGAACTTCGGCGTTGCCCGGCTGATGAGGGATAAGCAGACCAAGGAGCTCGTCG
CCGTGAAATACATCGAGAGGGGTGAGAAGCAAGTTTGCCATCGCGATCTAAAGCTGGAAAACACCCTATTGGATG
GAAGTCCTGCTCCTCGTTTGAAGATATGTGATTTTGGGTACTCCAAGTCGTCTGTGCTGCATTCACAACCCAAGTCC
ACTGTTGGGACTCCTGCCTACATTGCTCCAGAAGTATTACTCAAGAAGGAATACGATGGCAAGATTGCAGATGTGT
GGTCATGTGGAGTAACCCTTTATGTCATGCTAGTTGGCGCATACCCTTTCGAGGATCCCGACGAGCCGAAGAACTT
TAGGAAGACAATACAGCGTATTTTGAGCGTGCAGTACTCAATCCCAGATTATGTTCACATATCTCCTGAATGCCGG
CACCTGATCTCAAGGATCTTTGTTGCCAATCCCACGATGAGAATAACCATTCCCGAAATCCAGAATCATGAGTGGT
TTCTAAAGAATCTTCCTGCGGATCTCATGGATGACAACACAAGCCAGTATGACGAGCCCGATCAACCCATGCAGAG
CATCGATGAAATCATGAAGATCATAGCGGAAGCCACCATACCTGCGGCCGGGACACATGGCATCAACCAGTACAT
AACAGGCAGTCTCGACTTTGACGACGACATGGATCTGGAGACCGATCCCGACATTGATGTCGACAGCAGCGGGG
AGATTGTCTATGCGATGTGA

>Lchi00543

ATGGAAAACGCAGGGGAAGAAGAAGAACAGGACGGTCTATCTGTGCATTCTCCAGGCCAAGCTCCTCCATCTTCT
GCATCTTCTCTCCCCAAGGAACATTCACAGGTGGAATTGGAATTGAGAGTCTTAGAAGCTCTGGAAATCTATCCTC
CTTCCAAATTACAAGGTATATTTGCTATCAATTGGTTTGAAATCTCTGTCCTGGAAAGTCTCTGCTTTGATATTCAAA
ACATTGAGAAACTACCCTGTACTCTCATTGCCCATTAG

>Lchi25623

TTAGCTGAGATGGAATTCCCCGCTCGCATGCACTTCCTTGACCCGCTTGTCATATTCGTCCTCTTCATCCTCCTCTTC
CTCCTGATCCACGTCTTCTTCCTTCTCCTCCTCTTCTTCTTCCCCAACCCAGCCAAAGCCCCCGATAGACCGGGACGA
TGGCGGTGGGGTCCTTGCCTCCCCAACGATTTTCATTATGTCATCAACGCTTTGGAGGGAGAAGCTCGGGTTGTCT
CTCTGATAGTAGATTGCTTGTTGTGTTTCTGTCAGCTCCCTTGGCAAGTTCTTCAAGAACCATGGGTGGCTCTTAAT
TTCTTTAATTGTAATCCTCTTGGATGAATTTGCAACGAAAATCCGAGAAAGGAGCTGCCTGCACTCCTGGGATATAT
GAACATAGTCGGGAATTTTGTATTGGACTGCCATGATGCGCCCGATGGTCTTTCTGAAATTCTTGGGGTCCTCTTG
GTCTTCAAAAGGGTAAGCTCCCACCAGCATAACGTATAGAGTTACCCCGCACGACCACACATCAGCCAACTTCCCA
TCGTACTCGCGTCGAGAAAGTACTTCAGGCGCAATGTAAGCGGGCGTTCCCACCGTTGATTTGGGTCGGGAATGA
AGCAGAGAGGACTTGGAATAACCAAAATCGCAGATTTTCAAGCGCGGAGCTGGGCTTCCATCCAACAGAGTATTC
TCCAGCTTCAAATCTCTATGGCAAATTTGCATTGAATGACAATAGCTGACTCCTGAAATCAGCTGCTGGAAAAAAT
ACCTTGCCTCATCTTCGCTGAATCTCCCAGCGTTACAGATCCGCTCGAAAAGCTCTCCCCCTGCAGCATACTCCATC
ACAATCGCCAGATGCGTAGGCGTTAAAACCACCTCCTTGAATCGAATTATATTAGGATGGCGAAGCGATCTGTGAT
TTATAATCTCCCTCGCCACGTTCTCATCAATCTTGTGGCCTCTCTCGATGTATTTCATTGCGACGAGCTCTCTCGTAT
CCTTATTTCTCATAAGCCGGGCGACGCCGAAATTCCCAGATCCGATGTCTCTCACCAGCTCGTATTTCTCCAT

>Lchi12999

TCACATTGCGTAGATGATCTCCCCACTGCTGTCGACATCAAGTTCAGGGTCAGACTCTAAATCCTCCATGTCATCGT
CGAGGTCCTCACCTATCAAAAACTGGTTGATGCTGCGGGTCCCAGCCGCAGGTATTGTGGCTTCTGCAATGATCTG
CATGATCTCGTCAATGCTCTGCATGGGCTGGTCAGGCTCTTCAAATTGGTTCACCGTGTTTTCGTCCATAAGATCTG
CTGGGAGGTTCTTTAGGAACCACTCGTGGTTTCGGATCTCCGGAATGGTTATCCTCGTGGCAGGATTGGCGACAA
AAATCCTAGAGATCAGGTGCTGGCATTCAGGCGATATGTGAACATAGTCCGGGATAGAGTACTGCACACTCAAAA

TTCGCTGTATTGTCTTCCTAAAGTTCTTGGGCTCCTCAGGGTCTTCAAAAGGGTATGCACCCACAAGCATGACATAG
AGGGTTACTCCACATGACCACACATCCGCAATCTTTCCATCATATTCCTTCTTGAGTAACACTTCAGGTGCAATGTAT
GCAGGAGTCCCAACAGTTGACTTTGGTTGCGAGTGCAGTACAGAAGACTTGGAATACCCAAAATCACATATCTTCA
GACGAGGAGCAGGACTACCATCCAACAGAGTGTTTTCCAGCTTCAGATCGCGATGACAAACTTGCTTCTCGCCCCT
CTCGATGTACTTGACGGCGACGAGCTCTCGGGTCTGCTTGTCCCTCATCAGCCTTGCGACGCCGAAATTACCCGAC
CCGATGTCTCGGACGAGCTCGTACCGATCGCTGTCGTGCATTATCGGCATGTCCATGGCGGGGCCCACCGTCAGC
GCCGCTCTGTCCAT

>Lchi01348

ATGGAGGAGAGATATGAGCCGTTGAAAGAACTGGGGTCGGGGAACTTTGGGGTGGCGAGGCTGGTGAGGGATA
AGAAGACAAAGGAGCTGGTTGCTGTCAAATACATTGAGAGGGGGAAGAAGATTGATGAGAACGTGCAGAGGGA
AATCATCAATCATAGATCATTAAGGCATCCAAACATTGTCCGGTTTAAAGAGGTTGTTTTAACGCCCACACATCTCG
CGATCATTATGGAATATGCTGCTGGAGGTGAGCTCTTTGAGAAGATTTCCAGTGCTGGACGATTTAGTGAGGATG
AGGCAAGATTTTTCTTTCAGCAGCTAATATCTGGAGTCAGCTATTGCCATTCTATGGAAATTTGCCACAGGGACCTT
AAACTGGAAAATACACTCTTAGATGGGAGCCCAACGCCGCGCCTTAAAATATGTGATTTCGGTTACTCCAAGTCTG
CATTGTTGCATTCGCAACCCAAATCAACAGTAGGGACGCCAGCATACATTGCCCCAGAGGTTCTATCACGAAAAGA
GTATGATGGAAAGATTGCGGACGTTTGGTCGTGTGGGGTGACACTGTATGTGATGTTGGTGGGGGCATATCCATT
CGAGGATCCTGAGGATCCTAGAAATTTTCGGAAGACAATTGGGAGGATAATGACTGTACAATACTCCATACCAGA
CTATATACGCACATCAGCAGAATGCAGGCACCTTCTTTCTCGAATTTTTGTCGCTGACCCATCAAAGAGGATCGCAA
TCCCAGAGATAAAAAACCATCCTTGGTTCCTGAAGAACTTGCCTAGAGAGCTGATCGATTACGAGAAAACAAACTA
TGAGAATGTAGGCAGTGACAGCCTGTCTCAGAGCGTTGAAGAGATAATGCGGATCATACAAGAAGCAGGGACAC
CCGGTGAAGGTCTGAAGGTTGATGGTCAGTCTGTCGCAGGGTTGGTGGACCCTGATGACACCGACACCGATATCG
ACACTGAGGAAGTCGATGAAAGTGGCGATTTTGTGGCACGGGTGTGA
