# Supplementary material for: Genome-Wide Identification and Expression Analysis of SnRK2 Gene Family in Dormant Vegetative Buds of Liriodendron chinense in Response to Abscisic Acid, Chilling, and Photoperiod
Source: Genes (Basel). 2022 Jul 22;13(8):1305. doi: 10.3390/genes13081305 (PMC9331246; doi:10.3390/genes13081305)
Supplement: Supplementary file 1 [file genes-13-01305-s001.zip › Table S6.pdf]

Table S6. Genomic sequence of SnRK2 family in *Liriodendron chinense*.

>Lchi13910

ATGGATCGATCGGCGGTGACGATCGGTCCGGGGATGGACATGCCGATAATGCACGACGGTGATCG
GTACGAGCTCGTGAGAGACATCGGGTCGGGGAACTTCGGCGTTGCCCGGCTGATGAGGGATAAGC
AGACCAAGGAGCTCGTCGCCGTGAAATACATCGAGAGGGGTGAGAAGGTAATTTTCAGTGGATTCC
GTGGGCTTTCTCTTTTCGCGTTCTTGAAATGGTGTTTTTTTTTTTTTTTTGTTTTGGTTATGGGTTGAA
AATCAGTAAGTTGGTGTTTGAATTAATGAGTTTTGAGTGATTTTTGGAGCATTTTTATGATTTTTGGA
ACTTTTTTTCTTGAATTTTTGGAGTCTTGGTGTTGTTGGTCGACAGTGTGCAGCTGTGGAATTTGGTG
TTGAATTAGAAAGCTGTTAATGGGAGTTGTGCGTCTGGATTTACGCAGCATGATGGCTGGGCATTGA
GTCATTGCCGTACATTGGGTGGGCCCCACTATGTATAGTGCTGTGTCCCGGAGATCAGACTGCCATG
GTTACCATGTGGGCTGGTTTTGTTTGAAAAGGAGATGTGCTTGTGTTGCGACATGAGGGGGGGGGG
GGGGGGATCCTGAATTTTGGCTGGGAGCACATACATGGTGAGGCCCGCCCGATGGACGTGCTTGAT
CTTTCTCACTTGCACCATGTTAGCACATTCCTTTCGGAGGGTTTGGATGCATGAGTCCTGTTTGAATTT
CTCACTGAATTATGAAGATGGGTGTAGAACCAGAATTTTTGTTTTTTTTTTTTTTAATTATTATTTTTTT
TCCCGTGTTATTTGGAAATGGTTATGTTAAGGATGTGGAATTTTTGCTTATTTTGGTAGCTTGGAAAT
GCTGCTGTTGTGGTCTGGATTAAACTGATATCTGTAGAGGAGCTTTGTGGTTTATTGTGATGAACTGA
CATTGTTTCATTGCCATTCATCACTGTACATATGGCTGTATGATCGAGTGAAGCAGGACCGCCCATCT
GGTTGGCCTCACCATGTTAAGCCCACAATACAAAAATGGAAATGATTGGACGATCCTAGCCTTCAAG
TTAGAAGATCTTCCCCCTTTAAAATTGGACCATTGATTGTTTTCTATTTTCATTTTTCGTTTGAGGCCAT
GGACTGGAAACTGACATTGCCTTATTGGTGTGGTTTTCTACTTTTGCCATCTATGGAGTTGTGTTGAT
CAATCATGGCCACACATATGGTGGAGGGGTGATGAACCAGGACTAGTCCATCACCTGATGAGAAGA
GTTTGCCAGATTTCGATAACTGTAAACTATGGTTTTTCTTCACCGTGCCGACCTTGATTTTGGATCCCA
TTGAGCAAATTGGACACAAGACCGTTACATCAGAAAGGAATGCGATGTGCTGATGTCCTATTCCTTT
AGCTATGTGAGTTGACGGAACATAGTTTTAGTTAAAGAGATCCAGACCATATTTTAGTGATGGGCAA
CTGATGAAAATTTCAACAACCTTCTAAAAGAACAGAAAAAAAAAAAAAAAAACCAGTTGTTGGGGATT
TGGGTATTTTTTTCCCAACCAGCAACCAACTAAAACTTCATTAATGAAAGAAACTCAGAGACGCAGTG
TTTCACAATGGTACAAGAGACCTGAGAGGTCTAGCAATTCAAACTCTTAAAACCAGTGAGTCTTTCTT
GCTAGAGATAATAGTAACAAGTAACAAGATTTTGTTTGCATATGGGGTAAAGCAGCAACAGAAACA
ACTGCTATTATGTGGATTTATTGTATTATTTCTTGTCTCCTCATTCTGCTAATCTTTGCTACAGATAGAT
GAAAACGTACAACGAGAAATTATCAACCACAGGTCATTAAGGCACCCCAACATTGTTAGATTCAAAG
AGGTTTGTTTAGTGAAAAGTTTTCTTTGTTGTATCTGTTTAAATTGAAATGCTGTCAGTCAATATCTTA
CATCTCTGCTAAAATTCATTTTTAGGTTATATTGACACCGACCCATCTGGCCATTGTGATGGAATATGC
GTCTGGTGGAGAGGTGTTCGAGCGTATATGCAATGCAGGCCGCTTCAGTGAGGACGAGGTTGTCAA
TCTGATCCATCGATCATCTACCCCTTTGCATCCGTGTTGTTTTAATATCATGTTGTCCTCCTGCAGGCCC
GCTTCTTCTTCCAACAACTTATATCAGGAGTCAGCTATTGCCACTCAATGGTATGTAGAAGAGCACCT
GTTTTCAAATGTCATTTCTGGTCTGTATTTGGGATATCATCCAGGTCACTAAATCAGCCTACCTATGCT
GTATTTTATAATGAAGCAAGTTTGCCATCGCGATCTAAAGCTGGAAAACACCCTATTGGATGGAAGT
CCTGCTCCTCGTTTGAAGATATGTGATTTTGGGTACTCCAAGGTAATTGTTGTCTTTTGCATTATTTTC
CATTTGGGTGGTCATAATATGCTTGTTGGATTTTCAGAGTTTATAAGCATCTGGTCCTGTGCATCTTTG
TGAATGCCTATTTGCTCATGTAATCCCCCCCCCACAAAAAAAAAGGCTCATCTAATACTCCTACCCCAA
TCAAACATGAACAAAAATACATCATGCGGTAGGGTTTTGGAGCTGGTTTGGACTTGAGAGGTTTTGC
TCATATTGCCTCTTGCTTCGAAAATTCGAATAACAATCTCAAAATAGAAGATCTTGTTCTGTTGATTAG
GCATAAGTGAAACTTAAAGTCTCTTATTGTCCAGGCTATCTGGCACTGTTGCGAGCGTCATCATTTAT
ATTTGTGCAATTCTAACTTCAGTTACTTTTCTCAGTCGTCTGTGCTGCATTCACAACCCAAGTCCACTG
TTGGGACTCCTGCCTACATTGCTCCAGAAGTATTACTCAAGAAGGAATACGATGGCAAGGTAAGATA

TTTCTTACTGTTGAAAGTTGAAACTTGCATGTGAATAGTTTTACCCACATTTTTCTTAGTGTTCCACCCA
CATACATGGAATTTTTGTGAAGGGAGGAGATTGGTGTGCTTTTGAACTGCCAAGTTGGTATTCATAT
GGCTTTCGTTTCCTATGTTTCTTATGCAAATTGTAATAATTTGCTTCTTATTTTCTGGTAAATCATCTTT
AACCCTGGTGAAATCTAAGTTGATCGATATCCTGGACAATCACTTCAGGCTTGTTTCGGTTTCTTGTA
GAGGGGTTAAGGAACTGAACTTTTGAAGGAATGAAATTCAATAATCAACTGAGCCTCTTCAATATCG
TTTTTACTGATTTATGAAAATTAGTGAGCAAAACTTGAAATTAAAGAAAATGCGGTACTAGTGTAGAC
ATTAAAATCTGAATCGATATTAGGTATGTTAGATTGTCAGTATAAACTGGTGTAATCTTGAAGAAACC
CCAACATCATGGATTATATTCACAAGGATCATGGCAACTACACTGTTGGTGTTGATGCTTGTCGGCTA
CTCATTGGATGAAAACTCAGCTTTGTATGTGCGAGACGCCACTCACGTTTAAGTTGAAAAATACATGT
ACGTTCATAATTTTAGAAGTTGAGATTTTCTTCCTTGAAGATTTATTGTAGGACATGTTTGGATTGGA
GAAATCCTTGGTGTGGGATTGGAAATCTCAATCACCATGTATTGGTTATTTCCCAAGGCACAATGCAA
GAGTGGATTCCACTATAATAGGGGTCTCGTTTGGATATCAAGTGAAAATCTCTCATATCCCTCTCGCA
ATAGTCACTTCATATGTTGTGCTTGAGAGTTTAGATTGTGGAAAAGACTAATGATTGCTTGGTGTAGT
CCACCATTTCCTTGGCTATACAAACAACACAAAAGGTCTCATCAATGGAAAACCTTTTCCATTTCCCTG
GATTTCCATAGCTTTCCTCGTATCCTAACATGCACAAGAAATGGTTCATACCTTGATAGAAATCTTTGT
CAAAGAAGGGAATCTTGCCACATATATTTGTATTTCCTTATCCACAAGTGTCTATAAACTAAACCTCAA
ATAATGACAATTTGGTTTCAATGATTAAAACTTAAGGACAAGTTGGAACCGATGATACTGAACACGG
AAACAGGGATGTTAGGAAATCCAACACTTCATAACATTTAATATTGGAAAATGTGGCCCTATATAATT
TTAGTTCATGAATATGAGAGACACGGGAAATATATCTACATAACCAACTAGAAGCATAGAACTATAA
AAATCGCATACAAATGCTGTCAAAATACAAGAAGAAAAGCTAAATTAGTTTCTTCTTTTTTATTTTGTT
CCATACCAAAGTTGTTTCATGCACGCTGAAAACATGTCTGACGCTGGTCTTGGACATAATATTTTCTTT
TTTGTAAAAAGTCTTTGTTACATAATTTTTTTGTTTGTAAGGTGAAAGATTTATCAGGCAGAGCCGAA
AAGAAGAATACAAAGCAAAGAAAGCAAACAAAAATAAAATAAAAGAAAATGCAACAACCAGCCTCA
AACTGGCCAAAAACCCCAAAGAACAGAAGGACTAAGGAGCCTAATCCCTAAAAAGAACAAGAAGTC
TAGAAAACACCCCAACAGATGATACCTTAATGTTTCCAAAACATCGGTTGTTCCTCTTAGCCCACATG
GCCCATAAGATTGCCATTAGAAGAACCCTCTACTTCCTCCTCCCAAGAATTCCTCCTCGGGTCCGCGT
GCTAGATGCAAAAGAGACCATCCAAAGAGATGGGCATAGCCCACGAAACCCCTATCAAACGAAGGA
CACTCCACCATACATCCGTTGAGAAGGGACAATGGTTAAAGAGATTCTCCACTAATTCCTCAGCCTGA
AGGCACAACAAACAAATGTTCAGTAAGATCATTCCTTGTTTCCTTAAGTTGTCGATCGTTAGCGTGCG
ATTCCTGCTTGTGAGCCACCCAATTGCTGCAACCTTCGGGGGGACACTGTAGAACTAGAGCGAACCC
GTATAGCAGCACCTGGTCGGCGCAAGAGCAACAGATAGGGATCTGTATAACGATCGCACTGAGAAT
TGACCGGAACTCGCAAGAGGCCAACAAAGGGAATCATTCGAGCTAGACACAAGGGAATGATCCTAT
AATCGAGAAAGGAACTGAGTGAGATCCATAATTTCAGAGTCAGAAAGGTTCCTACGAAAGGGGGGA
AGCTAGACCCCTCCTACCCCTTTATTAGAGAAACAATCAGCTACCAAAACATTTGGATTGGGGCTGAT
AGCTATTAGGGTTGGGAAAGATTCAGAAAGAGAGGGGGATCCACACCGAAGATCCAACCAGAAAA
GAATCCTGAAGCCATTGCCTAAGGAAAATGATAATCTTTTGTGGACTTTAGGGGCCACCTTAGCTCTA
CCTTCTAAAGAAATGAAGCACAATATAGAGAGGAGGATTTAGTCCACCATCCTTGATGAGTTTATTCA
TATTTAGCAGAGATGACCATCCTCCAAAGATTTCCATTCTCACACCCAAATCTCCAAAACCATTTTCCC
ATAAGGGCCTTATTGACAATCTCCAATCTTTTTATGCCCGCTCCCCCTTCCTTGAAAGGAGTACAAATC
TCCATCCAGTTCAATAATGGAAATTTCCTTGAGGCCTATCCACCTTGCCACAAAAAGTTCCTACGAAG
CTTATCAATCTTCAGGATGACAGACTTGGGCAAATGGAATAGAGACATGAAATAAATTGGCAAATTT
GAAAGGAAGGCCTTTATCAAATTCGATTTGCCACCCAGGGATAAAAGTTGCATTTCTAGGAGGACAA
TTTCCTTTCTAATCTTTCAATGATCTTATCCCACAGGTGCAAAGGAGGTTTCCCCATACATAGGGGAA
GACCCAGATAAGTAGTGGGAAGACTACCAACCTTATAGCCAAGAGATTCAGGCAGGGAAGATATTA
CCTTCGAAGATAATTTGATCCCAAAGAATTCACTCTTCAAAAGATTCACCTTTAAACCTGAGACCACCT
CTAAGCAGCAAGTGGTCATGCTAAAAAAATGTCACCATATTGTCCTTAGCTTCACAAAACAGCAAAGT
GTCATCTGGAAATTGAAGGTGACAAATGCGCTAGGGGTTCCACCTGGAAACCCCTGAAAACCCTGAT
TCTTGGCCCTTGGACAACATACAATTAAAAGCTTCAGTAACAAGCACAAAAAGAAAAGGGGATAAG

GGATCACCCTGCCGAAGACCCCTGGATGCCCTAAAGAAGCCAAATGGGGATCCATTTATAAGAATTA
AATATCTAGGAAAGGAGACGGTTTCTAATCCAAGCCCTCCACTTAACACCAAAACCTAGCCTTTTCAT
GATGTAGTTTGTGAAAGTTCAGTTCACACGATCATAGGCCTTCTCAAGATCAAGTTTGCGAAGAATTC
CCAACCTTCTCGCTCTCAAACAGGAATCTAGACACTCATGGGCGATAAGCACACTGTCCAGAATTTGT
CTACCTTGAACAAAGGCAAACTGAAAAGGGGAAATGATCTTGGGAATAACCAGCCTTAACCTCTGAC
TGAGGATTTTAGATAGGATTTGTTAAGGGCTACCAATGAGATTGATGGGACGAAAATCTGTAAGGTT
TAGAGTCCCTTCCAGTTTGGGAATTAGGGCTAAGAAAGCGCTCCCTATCTCTGAAGGCAGTCTATTG
AATTCAAAGAAATTAGAAACAAAGCTGAGAAGATCCAGCTTCACTAAATCCCAGAAGGATTGATAGA
AACTGATCGGGAATCCATCTGGGCTAGGAGCTCTAACTGTATATTTAATCTCGTCAAGTTCCACGAGT
TTCTCCAAAAGAACAACATCCTCGGAAGGGACAAAACCGAAGGATAATAAATCATCAATATAAGGG
AAGGAAATATTATCAGAAAATAGCAAATTATAATAGAAGTTGACAATAGAACTGCAAATGGAATCCT
TATTCGAGACAAGTATCGTCAATAAGCAAGGAGGAAATAGAATTAGCCCGTGCCCTAGCACTGGCTA
TACCATGGAAAAACTTTGTATTACGATCGCCTTGTTTAAGCTTGATAGCTTGGGATCACTGGTACCAT
TTTGTTTCCTCCTCTTTCAGTGTAGAACAATAATCAGCATACAACTTAGCTCTAGAGGCTATCTCCTTA
GATAATTGATCCCCTTCCTCTTTAACATCCAACTCTTGAATAGCAGAAAGCAAAGAAGACATTTCCGC
TTCTCTCTGACCAAAGACCTCATGATGCCAAACCATCAGCTTATACTTCAGCATTTGAAGCTTTTTGAA
AAGTCTAAAACCTGGGAAATCATCAATATCAAAAGAAGCCCACTAATCTGACACTAGAGAACTAAAG
CCTTCCATTTCCAACCAAGCTAATTCAAACCTAAAAGGTTTAGGCCCCCCAATTTATTTCGTTACTATTT
AGAAGAACTGGGCAATGATCAGAGATAGACTTGGGCATACCTTTTTGGTGAGCAAATGGAAATTGG
TGGATCCAATCGGGAGAAACTAAAAATCTATCAAGAAGGGATATTGCCAAAGAAGACCAACCGTTA
GACCCTAGATCGACAAATAACCCCCCAGGTTTTGGATATGGTCATACTTCCTACAATATGTCTAGGTA
GAATAAATCGGTGCTGATTCCACTAACCTACAATTAACTTAAATAATTGTTGCATAAGAATAGTAATA
ATGAATAACATTTTGATCTTTCAAAAAAAAAAAAAGGGCTAACACTCTGCTAACTGTACATATTTTGTTT
TTGTATTTGTACTCTGATGTAGAATATACATTTCATGCATGCCAAATAAACAAATCATCTTTGGCATAA
GGCATAACAATACGATATCCTAATTATACAAGTGCTAAAAGAATCGAATATCATTTTCAGACTAGCAT
ATCTAAGGGGTTCTTGCTTCCCATGTCACTAGTGGCATTGCCTGCCAGCTACAACATTCAACAATTAC
CATGGAAAACTTTTTTTTCCCTAGCTTGTTCCCATAGTAGCCCACCTCATTTTGATTGATAAGCTATGT
CCTATGCAGTTCTTTTCTTTTCTTTTCCTTTTCCTTTTCCCTTTTATATCCGCACTTCATGAAAATCTGTTC
GGGTAGATGGTCTACCAATTATTGTTGTTACGCAAGCCTATGGGGTCGCTTCTGTAACTGGATAACC
AGCACTGAAAACCGTTGCCATCTCATTCTTCAACAGGCACGCGGTCAGTTATAGTAATTTTTTTTTTT
AATTAGAGGCCGTGAAATTTTTTCATTGTTGTTTTCCAACCACAAATTTTCTTACTTCTGTAAGTCTTAG
AGTAAGTGTAAATTTCAAAATTTCCCATCGTCATCGTCATCATCATCATCATCATAGCCTTTTTCATAG
ATATTGGGGCCGGCTTTATGAATCCTGTTTCCCCGAATCAATACTATTTAAGCCCCATTCCCAGTAAGT
GAATAGGCTAGCCTTCATATCCATTCATCACCTCGATCCAAGTCCTTCTAGTTCTTCCTCCCAGCCATG
TTGTGGCCCTTCAACCCTAATCAGTGTTCCCCTCCTCACCGGAGCTGTCCAGAAGCACATTACCAAAC
CATCTCAATATGCTCTCTATCACTAGCATCTTTTCTATTTCTTACTTCTTTCCCAATTTCGCTATGTTGAC
TCCTCACTAGCTGTCTTTACTGCTTTAACTCAGATTGCAGATGTGTGGTCATGTGGAGTAACCCTTTAT
GTCATGCTAGTTGGCGCATACCCTTTCGAGGATCCCGACGAGCCGAAGAACTTTAGGAAGACAATAC
AGGTTATCACCTTGTTCTTTTCTTTTTAATACAGTGAATGGAATGTCATGCGTTAATCAGTTCTAAGGC
ATTCCTTGCTTCTTGCAGCGTATTTTGAGCGTGCAGTACTCAATCCCAGATTATGTTCACATATCTCCT
GAATGCCGGCACCTGATCTCAAGGATCTTTGTTGCCAATCCCACGATGGTTAGTGGTCTATATTTTGA
AGGGCCTGAATTTGCTCTTGATAATCATCTGTTCAGGTTATTTGTTTTGCACAGGTAGGGCCTGTGGG
TTGGTAATCCAGACTGTTTGTCTGATGCATCCCCGCAGTGGGTTCCCGTGCACCGAAAAATCTTCCAG
ACGGAAAAATTTTCTAACCCTTCATTTTGGAATGTCACGGCCCACATTCAACTAAAAAAAGAAACACG
ATTCTGTTTTTTTTTTTTTTTTTCCTTGGTTTTTGGCATTGGCTATCCATGGTGAGGCCCATTGTATCCA
CTCTTTTGATCACTTAACCATGGACCCTCGTATTTGTTTGAACCAGCAGTCATACAGTCGGGGCCCAA
GATTTCACTTATCTAAGTTGTTCATCTGGTGGACCCCATTATGGGCAGGTCATAGCCCTGGATCTCCT
CTATCGGACTATCCCACCCGACATATTGGTTGCTTGTAAAAGCAATAATGGCCAAAGGTCCACATTTA

CTAGGGCATTGATCGGATGGTTAGCATTGCCCGAGGGGGAGATGTTTGGGTTATTATATCCTATCAA
AGTCAGTGCCCGCCCCACAAGCTATGAGGAACAGTTTGTATGATTAGTGTTTGCATTTTCTTCTGGGA
TTGATGGTGATGACTTTTCTGGTTAGTGAAGTGCAGAGAAATCTATTTTTACGTTGAGAGCTTCTTTT
TCATTTTTTGGTTTCTAGTCTCAGGCTCTCAGCTTTAGATATTTTGACTGAATTACAGAGAATAACCAT
TCCCGAAATCCAGAATCATGAGTGGTTTCTAAAGAATCTTCCTGCGGATCTCATGGATGACAACACAA
GCCAGTATGACGAGCCCGATCAACCCATGCAGAGCATCGATGAAATCATGAAGATCATAGCGGAAG
CCACCATACCTGCGGCCGGGACACATGGCATCAACCAGTACATAACAGGCAGTCTCGACTTTGACGA
CGACATGGATCTGGAGACCGATCCCGACATTGATGTCGACAGCAGCGGGGAGATTGTCTATGCGAT
GTGA

>Lchi00543

ATGGAAAACGCAGGGGAAGAAGAAGAACAGGACGGTCTATCTGTGCATTCTCCAGGCCAAGCTCCT
CCATCTTCTGCATCTTCTCTCCCCAAGGTCTCTCCCTCTCCCTCTCTCTTTCTCCATGATCTATGCAGCG
ATATTCTCCACTCGATTTCATTCATTAATTTCTGTCCTTTCTGTTTTCTTTGGAATTAGGAACATTCACA
GGTGGAATTGGAATTGAGAGTCTTAGAAGCTCTGGAAATCTATCCTCCTTCCAAATTACAAGGTATAT
TTGCTATCAATTGGTTTGAAATCTCTGTCCTGGAAAGTCTCTGCTTTGATATTCAAAACATTGAGAAAC
TACCCTGTACTCTCATTGCCCATTAG

>Lchi25623

TTAGCTGAGATGGAATTCCCCGCTCGCATGCACTTCCTTGACCCGCTTGTCATATTCGTCCTCTTCATC
CTCCTCTTCCTCCTGATCCACGTCTTCTTCCTTCTCCTCCTCTTCTTCTTCCCCAACCCAGCCAAAGCCCC
CGATAGACCGGGACGATGGCGGTGGGGTCCTTGCCTCCCCAACGATTTTCATTATGTCATCAACGCT
TTGGAGGGAGAAGCTCGGGTTGTCTCTCTGATAGTAGATTGCTTGTTGTGTTTCTGTCAGCTCCCTTG
GCAAGTTCTTCAAGAACCATGGGTGGCTCTTAATTTCTTTAATTGTAATCCTCTGCAATTTTAGAGAAA
ATAAGTATTCAAAAATGTTTATCCCCTGACCGATCATTTCCTCCCTTGTAGGAGGGTTTAGACAGTTAT
ACTACGTAACAATTGTGATCCATAAAGAAAAGGACCATTCACATTCACATATTGAACAAGACATTGG
AGTGAACCAATCCTCCCTTGGGTGGCAAGGAACCATTCCAAGCCTAAGCAAGTGTGATTGACAAATG
CTGCCTTTCCCAATTGTATGTTGTAGGCTACAGATGGAAGGTCAGGATCTTGCAAATGGGAGATTTTT
GGAGAACCACCATCCAGTAGGATCCATCAGATAATGTATGGTCTGGATAACCAAACTCTAGGCTTAC
ATGTACAGATGTGCCTCAATAAAGGGAACATTTGCCGAGACCTAGGGTGTTATAATTCCTCGCTTGT
GTTTTTGAATTCACAAAAACAAAAGTGTCATACTATGGAGCATATCCTAGCATTAGACAAGTGGATG
GTAAGGTCATCAACTCATGGTATTAAAAAATGGTCAATAAGGTAACGGTGGGCAGTGTTACGTGATA
CGGGGCCGTGACAGCCATTAAAAAAATAATAATAATAATAATAAAAGGTCTGCATGGGCTGGTAAC
GGCTGTAACGAGCCTTTACAGTCCTTTAAGGCATGTAAGCCTGTAATGGACCATTTTTGTTATATATA
GTAACAGAATTTTTCTTCAAAATTCTTACAAAAATGGTACAAATGATTAGGGATGTCGCTTAGTGGGA
TGACTCCCATAGATTGCAAGAATATGGATATTTTAATAATGGAAATTTAGTCCAAACCTACATAAAAT
GATACAATCCATTTATCCTACACAAACCTTTTATCCTACATAAAATTTGATTGCAAGAAATTTTTTTTT
TTTTTTTTCCTCAAATTTCTTGGGGGAAATGGTGTTATAATGATTAGAGATATGCATAAGTCTAATCAT
TATACACCATTTTGCGATATTAAATATAGGAGTTACTAGAGTCAATAAGGTAAAATGAACTCAGGCA
GCCAGAGACAGCATTCTTGCCAAAAAGTGAACGTTTTCACCATCACATACATGTTCAAAACCAGTAAA
CAATACATATTTGGGATCCTCCCATTTTTCTGAAAGCTTTTAATATTCTTTCACTTTTTTATCTTTTTTTT
TTTTTTTCTTTTTTTGGACCATTACAATTCTTAACGGCCATAAGGCTGGCTATTACAGATTGAATAACTT
GCTTCAACCAGCATGACAATTGGATTTGTCTACAACAAAATACTAACATCGAGTTGAAAATATCTGTA
ATCTAATGTTCAGGTTCTATGTACAAGTTATATGGGAACTGGTATGCATGTGGAAAGAGGGCAGGA
GCAGTGGTGGCAGAGTCTAGAGAGACTAATGAGTGTGGTGCTTTTCGAATAAATAGCTGAGGGTAA
GTTTAGTTCCACCAATTATGTACCATGAAATTTCATGGATAGGTACTTTAGAATCTAAAGGATTTAAA
ATTAGTTGGACTAAAACAGAGTATATGAAGTGTGAATGAGGAATTAGTAAAAATTGCTGACCAAGCC
GTTTTCCAAAATGACCACTTCTGATATCTTGGGTCAATAATTCATGAGAGTGGAGAGATTGAGAGGG

ATGCTACCCATAGAATTCAAGAGAGGTAGAAGAAATGGAGATGTGCCTCTGAAGTTTTATGTGATCG
TTGTGTACCACTCAAACTGAAATGGAAATTTTATAGGATGTCTATAAGACCAACCATGCTTTATGGGG
CCGAATGTTGGGCAGTTAAGGAACAACATATCCATAGGATGAGTGTAGCTGAAATGAGGACATTGA
GATGAATGAGTGGCAAAGCTGAAATGAGGATGGTGAGATGAATGAGTGGTAAAGCAAGGATGGAT
GGAATTAGAAATGCTTGAATTCGAGGGAACTTAAGAGTTGCAGCAATAGATAATAAGATCTGGGAA
AGTAAACTACAATGTTTTGGTCATGTGAACAGGAGACCAAGAACCACGCCAATTAGAAGTAAGGTC
GTACAAGTCAAAGGCTCTAAAAGGGCAAGGGGAAGGCTCAAAAGGACATAGATGGAGGTAATATG
AAAATACCTTCTTTTTCTTTTTTACACACGCACCCCACCCAAATGCACACAACCCCTACCAACGAAGCA
AGGACTCGAACCCTATGAAAATACTTGATGACCTATGGTCTAACTAAAGTTATGGCCCTTGATAAAGT
GGAATGGCAGAAAAGGATTCTTGAAGCTAACCGTTATTAGTTGAGGCTTAGTTGATGATGATTCTTG
TTGGTACAAGTAGAAAGAATGGGCACTGGCTGGGTCAAGATAATGATTCCCTCTCTTAAAAATAATT
TGTATAACTATTTAAATAAGATTTATGAATTTTCTATCATATGTAAAGGGCATTTTATGATAAGCTAGT
ATAAATGGCGGCAATCAGAAGCCGAGGGTGAATTCTATACTTATTAGAGGAGCCAGTGTTGGGGAA
GCAGGTGTAGGTGGTTTATGACTTTCCTATACAAACTTTCCTAACATGAAATCAGCAAAGCAAATTTA
TGGATTTATACATATTTCAGTGGGCTTCTTTCTATTTTGTGCAGTCAAGCAACTGAGCATTTCCTTTCC
TTTTTACGTCAGATGATGGTACATGTGTATGCCATCGTCCTTGTGGGCTTGCCTAACACAGCATGCTC
TAGAGACGAAACATTCACGCATTCCCCATTGTGGGTATGTGGGTAGGACTGTAATAATTGAAATAAC
ATGCAACTTGGGCTTATGACCATTTTGGCATGCAAGTGGTTAGATATAACATGGATAAATGGGTTCC
TTGCTATGTAGCCAACCTTCTATCTATGTGCATGACTCATGCATAGTAATGCTTGACAATGCCGTCTCC
AGCTTGTTAGTCAAGCCAGTAGAGACGTATACCACTAGGGGAATGACTAGACTTCGAGCCTGCATAA
GTTCCTTACCGACCCCTTGGAGGTGGATCCTCTGGTGTGGTGGATCCTCTAGTGTCGACAAATTTACT
TTTAGTTCCTCAGCTTTGCTAATATTGGCTATGATGTCATTTTTATCCTTCGAATGTATTGTGCAAGAC
CATGATTCAAGGAGGGCAGTTGGTAGCTGCCCTAGGATTGGCAAGCTTTATAGTGTGCATCACCTCC
ACCTTCCACTTTTGCTGCTTTCCTTGCTTTTCTTCCAACCTTAGTTTATAATTTTCTTTTATGGCATGGTA
GATTTGGTCATGTCTCTGCTTCTAGACTACAATATATAGGTTCTTCTAGTGTTTGGGGACGACTCTCAA
CTTCTGGAATATCAGATTGTAAGGGCTACAACTTGGCAAAACAATTGGCCCTTCCATTTTGTAAAAGT
GAAACTGTTTTTGTTGCTGCCTTTGATCTTAATACACTTTGATGTTTGGGGCCTTGCACCTCAATTGCC
TAAAGGTGGCTCGTCTTACTGTGTTACATCTATTGATGATACCCACGTTGTATATGGGTTTATCTGAT
GCATCATGGGCCCAAGCTATTATTAGATTAGAACTTATTTCCATGACTCACACTCAATTCACTATTAAA
ACCAAAGTGTATCTTTAATGCAAGTGGAGAATATTTATCCTCCTCCTTCCATGCTAGTCTTTCTTCACA
TGAAATCATTCCTCAATTGTCATATCCCTACACTCAACCAAAATGGGGTTACTGAGACGAAACATTAT
CACATCCTTGAGACCGTGATGCAAGACGACTAACCACTTGCTCTAAAAGCTTGAACTAATAGAGCGT
GACGAATCAAGTCCTTTATCTCATAGCCCAGACCCCAAGTCCATGAGTTAGGTCCTCAACCGAACCCT
CCTTCGTGGGCCCTAAGTCCATGGGTATATATCACATGAACCACCCGAGTCCACAGGTGGGGCCCGA
ATCCATGAACCACCCACGCGAGTAAGGCCTGAGTCCACGAGCCACCCAACTCACACGGGCCGCCCAA
CCCGAGTGTGTCCCCGCTTCCACAGGCTACCCCACTCGAGCCTGGTGTGACAATGCCCCCACATTAAA
CTGCTTGGTCTCTTCTTCTCTCTACTTCTATTCCAAGTCAGTAATGGAAAGAGACCTTGATTTCTATTCA
TCTTATCAAACACATTCCTTTATCTATCCCATGTGGTATATCTCCTCTCAAGCACTTATATTATGTTGGA
GGCAATGGAAAGACTTCGTTACTGAAGTCAATCTAACAAGTCTCAACTAGATGTTTTCTTAGCATCTG
GGGTATGCAAGTTTATTTTTGTTCCAAGTATATCTTCTCTCTACTTTTGTTCCAAGTTAGGTTTGGGTA
GAGGATTTCTTGATTTCTGTTTATCTAATCGATCGGATTCCTTCATTTGTCACATCTAAGTACAACTCCT
CTCAACATATATTCTCGGATGGAGGTGGTGGAAAGATTCTTGTAATAAAGACCGTCAGACGGATCTT
GGTCGAAGATTTTCCTTGTCTCCAGGGTAGGTGAGTTGGATAGGATTGTTACATTGGGATACTATAC
CAAATAAAATATGATAGGTCCTCAAATTCTTGAGAGAGTGGACTATTATCTCACTGAGACATAGGCTC
AATTACTCCAGGTTGTCTTTGGTTGCACATATTTTCTCCCCCTAAAGCATAAACATACTAATCTTTCTAC
TCGGTCTGCAATGTGCGTCTTCTTAGGTTATAAGATTAAGCATAAAGAATATCAATGTATGATCCCTC
AATTGGTTGAGTCCTTATCTTGCCATGTCTTCTTTCACAAACAAATCCCATTTTATTCATCATCTTCCCA
CACCTTCACCTCCGCTCACTAACCTAATCTCCTTTCTTAGTCGTTTTCCGGACCCTCCACTTGGTGAACC

TTCTTTGTGAATTAACACTTCATCCATTGATCCTCCACCTTTGCTCGATGACCATCCACCTCAGGTAACT
ATCCTAAGGATCCCCACCCTAAAACTATCCACAAGTTTATACCCGGTGAATTCCTGGTCCGACCTCAC
CAACACCTATTGCTTGTCCTATTTCTCATACTAATGTGGGACATATTCCTTCTGAGCTATGTCCTCGTTA
ATATGTTGCACGTGAAAAACGCCTTCCTACTCACTATTGTATTTCTACTGATGACCTTTCTACCCAACA
TATTTTGTCTTTTGCACTAGGCATCTGGTATTGTGAATTGGCAACGAACCATGGATGAAGCTCTCTAC
ACCAATCTATACTCATAAATGAAATGTTATCCTCTTCCTAAGCAAAAGAAACCCATTAGTTATAAATG
GATTTACAAAGTCAAAACTCACTCCGATAGCTCTCTTAACTAGCATACGGCTTGTCTAGTTGAGGAGA
CGGGAGTATGGCATTGACTATGAGGAGACTTTTGCTCCTATGGCCAAAATGAACTCCGCCTACCAAC
GTATGCAAAACGGCAGCAACTAATCATAAAACCCATGTCAATCTACCTCTTAAAAGTTGACCCCGAAT
TATTGGGACGAGGCTAAGATGGCGACAGTGATAATGATGATGATGGTAAATGCTTGAATTTTCAATC
TAATAAGGGGAATCATCATGATCATCATCAAAGCCTCATCCCTGATATTTGAGGTTGGATTATTCTTA
AGATGCGGACAAAAGTTTTGAGGGTTTGCTGCTCACCTTAAATATGAAGTAGTTAAAGTTCTATAGA
AATGTATAAAGTATAAGAAAAAGATAAGCCAAACCCTTGGTTAAATACTGGCATTAGTGTTTTAAAT
AGCGAATAGCGTGTAGCGTAGCCTTCACCTCTCTGAAGCGTGAGGCGTAAGCTACATAGCGTGTAGC
GTAAGCTACACAACACTTAGATTTTTAATCAAGGTTGCACTTGGTTGCACCAAATTAGATGATTAATA
ATAAAATTTAACAAAATTTCATGATATTTGGTGCAACAAATGCAACCTAAAGTAATAGCAAGGGTAA
TGATTAAGTATAAAAGTGCAGAAAGAGCAACAAAACCGATTTAAAACTATTTCTTATATTATTTTACT
ATAAGCATCGAAAAGACTAATGAATTTTCATTAAAATAGAATTATGTAACAAATCATTTTTAATGATT
GATTTTAATGTTGTCGTGAAAAATGACTTGTAATGTGAGCTATGTAGCGTGTAGTGTAGTTTATGAA
GCGTGTAGTGTATCCAAATTAATCATATAGCGTAGGCTGCACGCAACTTAAGCTATTTTCATGAGCTA
CGTAGCGTTTAGGCTAAGCTACGCTACGTAGCGTAAGCCACACGCTACATAGCGTAGCTATTTAAAA
CACTGACTGGCATAGGGGTATTATGTAGTGATGCTATAAATACTTAAATATGATCACATTTTTCTAAT
GCAAAAATAAGGTAATTTTCAAAATGATTACAGTAAACATCACTGAGCTACTTTATCAATAGATGTAC
AGTGTAGGTTTCACAAAGTTCAAGATTTTCACTTAGTTGGTGACATAGAATTGTGGTATCATGATATC
GAATGCAGTTATATTGTACATATAGCATGTGTTTGACATCTAGAGAGTCTATCTTGTGGAGCCCAGAA
CATGCATTGATAATTATCTAAAAATGAGATTATGGAAACAAACCGACAAGATCACTAGTGGAAATTG
ACCAAAAATAATTGTTTTTTTTTAAATGAGGAGCCACCACTAGCTGACGTATCTCTGATCTACGAAAG
GTTGGAGTCCATAAAGCCATGAAATCCGAAGGTCAAATTGATGACTTTGATTCCGACTGAATGACTC
CAAGTCTGGTTTAGCTTAAGGAGATTGAAGCAAGGGCCTTAATTACAGAGAAGGATGTGGTAAGGC
CCCTTTTCTGCCCATGTGTGAATGCGGTCTCTACTTTGCAAGATTCAAACTTCTTGGATTAGTGCTCTA
AATAAAGATCCACACAATAATGGCAAACGACGATACTAAAGCATATAGTAACAAAAATAAAGAAGA
TCATGACTAATAATGAGAGCAAGGGAGGTTTAGAAGAGATGGAAATGTCACAATCTAGATAACGAA
AACTCCATTTGGTGGTTTGCAGTAGTTCTCACCCTACTCCTAAAAGTTTAGTTTTGTGGTGGCTAAAG
GTGTAGAGAGGCAACAGGTAGTGTGAAGAGGCTACGGGTAGAGAACTAAGGGTCCAAGAAGAGAC
AGGGTGGCATTGAGAGGCTAAGGTTGGAGACTAAGGATGTAGGAAGGCCTAGGATTTTCTCATCAA
GGACCAAGGCACTCGAGAAATCTGGTGGAGGAACCTATTTATAAATGACATGGGTGGCTTTTTAAGT
AATTTGGGTGCAGTTTAAGGGCAAAGGATAGATAGCATTGTCTTATTTGGTCATGAATGCAAAGGGA
CATATGTCACCTCCTCATAAGGCTATGGCAGTAGCAAGACTTTTAATTTTGGACTTCCCTTAAGAAAA
AGCATGGAAATGTCACGTCAAATTCTAAAAAAAAAAAGAAAAAAAAAAAAAGAAAACAGAGAGAG
AGAGAGAGAGAGATAAATTGAGAGGTAGTCTAGAAAACTAAAATGCTGATTATTATGAGGGGGAG
GTGGCAAACTAATGAGGACACAAGGCCATTTCAAGTGTACCAACGAAGAAAGCGCCATCCACAAGT
ACCATCCTTGTGGATAGGCGACTATTGAGGAGCTGAAGACCCTCCACACGTGATCACACACATAGAA
GACCCCATCCAGGGAAGACACGTGTGTAGGAAGAACCCAGAAGACCATTGCCTTCAGGCACCCGAA
CTTAACCTCTTGAGCGCCCGAAACTCTCTGGACGTTTTTGGACGTCTTCGGGCGCCCGACCGAGCGCC
CGAACTTTTGCGCAGTTTTGCTTATTTACGCATTTTTGCTTAGTTTAGGGATAGTTTAGTACTTTTACTT
GTTTTATTTTATTATTTAAGTGGGCTAGACCCTAAACACGAAAATTAACTTTGATATTGATTAATGAAA
GCCTCTTTGAGCTTCTTCTTGCCTCCTCTCTCTCTCATGGTGATTCTTCTCTTCTCTTTGATCTTCTTCTTC
TGATTTCTTCTTGTGCCCCTCCAACTTCTTCCAGGTATTTTATCTTATGTTATTATCCTTCTAAACTTCTG

TTTTGGTTATTCCCTACGATCTAAACCTTGAGAATCGATCCAAACCCTAAATCCCCAAATTCTCAAATC
CCTAATCCGAGAAACTTCCTGATTCTTCGGACTAGTGATTTTAATTGATCGATCGGGTATAGCCATGC
AAGATCGGGAGGTTCCATCCCTCGCCGGATCCAACCTATGCACTAATTGAATCCCATACTCCATGGTT
GTGATGATGGCCCGTTCCAATGCATGTTTCCTTTATGTTTTTCTTAGTTTATGATGATTAATGCTAGAT
TTCATTATTATTATTGCTTTTACTTCCGCTGAATAATTGTGTTCATGAGCATGCATCATATTTAGGGCT
GTCCTGCATCACAAACATGTCAAAATGAGGTCACTCACATTCCTGCATGCTTGCTGTAGACCCATCCT
GGACATATGTGGTTTAGTACATGCAAGCTAACACACATGTAGATATGAGCACCTTATTGCGATAGGA
TCTGGTGGATTAAGTGGGTCTCAAGCTCAATTATTGCGAGGATTGGGAATATTGGAGTGGCCCTCTT
GATCCAATCCAGTCAAACAATTTTTGTTAACCTTTATTTCGAGTGGGGCGTCTACATAAAGAGAAATC
CCCATTGGAACGAAATTTCAAAACACATCCAGAAATTCTCAAAATTTCTAGGGACTTCCAAACATTCT
CAAAATCTAAGGTTTTCAAATTTTCAAAAAGAAATCTTCGAAGAAAAATTCCCAAACATATTTGACAT
TTCTAAACCAAAAATTTACAAGCCCTTGTCATCTAAGGCACCAAGGAGACTTAAATACTCCTTGGTGA
GTGGTGACTCCACAACAAAAATCAAGTTCGGCTTTATCTAGATTCCTAAGGCAAATCAATGTGGTCA
GCCTGAAACCAGCACCAATTTGATCACATCACATACAAAAATGGAAATTCAATCTTCAAGTGTGCAAA
TATCCCAATCCCATCAAATAGTTCATACATCTCCAGCAAAACAATCATCCTGGTGGAAAGCTAAAATT
GGAAGTCTAATTAATGAAGGATAATATGCGCAACCATCTATCAAACATTTCAATCCATGATAGGGGT
CAAGCCCATCACAACATACACATAGCTTGGGCCATCCACACAACAAAAGGATCCCTATAGTGTCGTA
GGATGCGTAGGGCATCTACTTTAACTCCAAAGGCTCGCTAGATGTGTCCCCGTAAGGCTATAAAATG
CGTAGGACATCTACCACCACTAGCGTTGGACATGAGCCTGAAAAGCTCGATCAACTCGCTCGGATCA
ACTCGAAATAGCTCGACTCAGGAGGGGTTCGAATCGAGTCCGAGCTGGATTTTGGGACTCGAAAGC
AGTTCGAGTCTAGCACCGAGCTGGCCCAGGCTTGACTCGACTCAAATCAAAATTTGGCTCGAATCCA
ACTTGGTTCGACTCAAATCAGATTGACTCGTTTGATCGGGTATAAATACCCGGTCGTAAGTAACCCTA
ACCCTTTTCAAAAAAAAAAAAAAATTCTCACCCATTTCCCCCTTTCTCCTCCAGCCCTCCAGCGACTGGT
CGGCCTCCCCCATTTTCCATTTCCCCCATCTACTTCCAACCCTCCCATTTTCCATTTCTCCCTTCTCTTCC
ACCCCTCCAGCGCCCGGCTCAACAAATCCAACTCGAAACATGAACTTGAACTCGGACTCGGCTTGAA
CTCGCTCTGTCTGCTGACCGAGTCAGACAGTTAGGCTTGAAGCCGAGTCGAGTCGAGTACCAAGCAA
GGGGTGTATGTTGGCCAAGTCGAGTCGAGCAGGGCCAAGCTCGACCCGGATTGACTCGTGTACAGC
ACTAACCACCACCCACTTCAATCGTGCATGAGTCATGATTCATTAGACAAACTACCAACACAAGCTCA
AACTAGAATGCTTGAACCCAACTTTAACTACTTGCATATATGGTAGGGTGTCATAGGCTTTGTCATTC
GGTTGCACGCAACCTTCACCAAAGAGTGGCAATTGTAGACATCCTACCCTACATGACGGTCAACAAT
ACCATTTGAGCAAGCTGAATCAAGAGAGCCGAACCCACTACTCTCACTCCCCACCGTAATCAAGCCTT
ATAACTCATCCAATGCCAAGATCCTACCATAATCAATGTGCCGGAACGATTCCTATGTCCACACGTGT
CCTCCTACACATGCCAACCCATGTGTCCAAGATGGGTCTTTAAGAAGCGGGTGGGATCGCCAGCATA
TGACCACATTTTGACATGTTTTCCACATACCTCCCACAATAATTACCCTTTTAAATTCATAAATTACGCT
TACATTTATCTCTAATTTTAAGAAATTGACATGTGGCACTCCCCATGCCTTCATCCTTAAGGAAACATT
GAAATTATAAATCTTGGCACTTCGTGGCCTTACAAGGAAGTGACATGTATCCCATTGCATCCATTGGC
CAAACAAGGGGACGCCCCATGTCCCTTTTCCCTCCAACATCACCTAAATTACTATAAAAAAATCCACA
CCTACTTTGAACTAGTTTCCTCATCCCATTTCTTAAGTGCTTTCATCCTAGGTGAAAAAATCCTTAATCT
CCCATTTCTCACGTTCCTTCATACTTGAACCCTTAACCTCCCTACACCTTACTCCCACACTTAATATTCA
CCTTTAGCCTCTCCACACCACCCTTACCCTCTCTACACCACCCTTTGTCTCTCTCTAGACCCTTAGTCTCT
ACCCTTTACCTCTCCACACCACCCCTAGCCTCTCTAGACCTTTAACCACCAAGGATTTTCCCTTTGGGC
ACTGGTACGAGGTTCCACCACAAGAAACAACCTTGCAACCGTTGCAGCAGCTAATACTATGGGTTGT
GCTTTCCTCCCTTAAATCTCCAGTCAGGGATCATATTTCTAAGAACTCATTCTTGTATGAAGAGACCAT
TCATTGTAATGTAGAGGAAAGAGAAGGGTTACAAGATCTAAAAGTCATAGGAATCTTCCATTTGGGC
TTTGGTACAAGGGTCCACCGAGAGGAAAAGCCTTCGACCATTGCAGCGGGGAACATTCCTCTCTTAG
ATCTCTAATCAAGGCCATCATTAAGAAACCACTACACACCATCTGACAGAGTTTCCATTTATCTTTCTT
GCAACATTTCCATCTCCTCTACCCCCCCCCCCCCCCCCCCCCCCCCCCCCACTTGCTCTTTTTATCTTGCA
TTATTTTTTTTATTTTTTTTTTGTTATTGTTTACATCATGTTCTTTGTTTTGCCATTATTGTGGAGATCTTT

GTGTCGAGCACTAATTCATGAATCCTACAAGGTAGAGACCAAATGCGAGAAGAGAAAGTGTCCCAT
ACCTTCTTCTCCGTAACCAAGGCCGTTACTTAAATCTTTAAAAGCTAGACCATACTTGGAGTCATTTAA
TCAAGGTCATCAATTTGACCTTCAAGTTATGTGTCCCTAGACTCTCTACCAACTCTAGCCTTCCATAGA
TCCGAGATTTGTCGACTAGTGGTGACTCTGCATTTTTTTATTAATTTTGTCGAATTTCCACTGATCTTG
CCAGTTTGTTTCCATAGAGATTGATTTAACTCTAATGTTTTGATTCGAGGATTTTTTCCTATTAAATGT
GGTTTTGTGTATTTTGTCCCCATTCGTCCAATTGCAAGCCATTGGCCACCAATTGGGCTAAAGGCCAC
TAACAATAGCATTGTTCCAAATATTGGAAACAAGTGCACCGCATGTGGAATTTAAATCGAGGGCCAC
TAAGTTGTTGCATCCCTCACACGAATGAGGGCACACCCAGGGATTCAAATATCGGTATTGATGCACG
TATCACACCTTCAGATGCCAAAACATATCATTATAGTATAGGAAATATCGCATGTATTGGGAAATGTC
TACTTCTTTGAGGGAAACATTGAAATTTTAAGATGTGTTAAAGGAGACATGATTATATGCAGAACAC
AAAAGATTACAAAAAAAAGGAGTATGTACAACAAGTTTTCTTTGTATTGTTTGACAAAAGTGATGCA
ATTCTATCCAAAACAAGGTATTCCATTATGGTAACAATGACCATAATGGCCACCGTCGCTACCATTAT
AATACGGGCCATAACAGACGTTGTTTTTTTTTTTTTTTATATATAACTATTTCAGCCCCCCATAATAGAC
CATTACTGGCCAATATGGTAAAATATGGTAATGTAACGATATATCGCCGATACATTGCATAGTTTTGA
AGGATACAAGAGAATTTAATAACTCCCATTTATATCGTATCAGCCACATGGATATCAATAGTATAGGC
TGATACTATCGATATTTAAATCACTGGGCATATCCATGTATTATAATCAGGAATGATTTATTTGTCTTT
GGGAGTAAATTCTGTGGATCCCATTGTTTTGCAATTTAATCTAATGGAATGCCCCAACTAGCTTTTGG
AATAGGGACCACGGAATACATCGTGGTCCCGTATTCATTAAATAAATAAATCATAGTGGTCCCATAAT
GAGAAGCGGGAGACTTCAATATATGGAGACTAACTTCTTTTCTTATATTAATAAGGGAGCCCTTTCCT
CTTCTATCTTTTTCAAATTAAATAAAAGTGCACAGATATGTTATAAAGTGTATAAGATGCATGATTCAT
CAGATGGACATCATACTACCTACCACAAAACTTTTGGCAGGATGGACATCGACAGAAGTCATACAAA
TTGCAGAGTCAAAACACCTTACCACCAGGTATATGAATCTAGATGGAAAGATTTCACTACATATGGA
GAATTTGCCTATGATACATCAAAATTCCTGTTTCCAATTAGATTGGTGAAACAAGTCATACAACCAAC
CACAGATAGCTAGGACAAGATCATAATGATGATGATGATAATGATATGATGAACTTCCCTATTGTAT
AATACTATGCACTTCACATGTTTATTTGTGCTAACTTCATCATGGAAAAGAATCCGGCATTACCATGA
ATATTTAAATTAATTATCTAGCTTTATCTAGGTGGGCATGCACCCATGAATAGATAAAGATTGTTCAT
CTACCAGGCACTACCATGAATTCTGTACCCAAAAGGTTGGACAGTTTAATCACTGATACATGGACTTC
CAATGATTGGAAATGGGTGTTAGGGAAGAAAAACTGCAACCAACAGTCAACATTCAATTTGCACAA
GAATCCTCCACTCAGCAGCCAGAATCATCCATGCTAGAAACCAAATGAACAGTCCAAATATCATACAT
GGGTGGCATATTTGCAGAGATTAAGTGCTTGACTAATAAGTTAATACAACCATTTAATAGTGTCAGG
TTTGGAACTTCTTTACATCTTGTCCTATCATGTGCCACTTCCACATGGCAGTGACACGTTTATATTTTGC
CAATGGTAATGCCTACAAAAGATGAGCCAAGCTTTCCAACATAACAGGGATGCAACACCTCAAACAA
GAACCATATAGCACTTGGGACATACCTTGGATGAATTTGCAACGAAAATCCGAGAAAGGAGCTGCCT
GCACTCCTGGGATATATGAACATAGTCGGGAATTTTGTATTGGACTGCCATGATGCGCTGCAAGAAA
ATTTCCAACAAGGGCATTAGAGTGATGAAACATAAACAGGAAAAAGAAAAAAAAAAGTAGAGTAGA
GAGACGGGAGGCAGGGCCCACCCCGATGGTCTTTCTGAAATTCTTGGGGTCCTCTTGGTCTTCAAAA
GGGTAAGCTCCCACCAGCATAACGTATAGAGTTACCCCGCACGACCACACATCAGCCAACTGCATTA
ACCACAGAAAGCAGTAAAAAATGAAATTTAAACCTTCTGAATGATGCCCATCTCAAACTGCCTAGTA
ATACACTATTGGCCCCATTTCAAGGATGCAAATTAGAAAATCTAGGACTGGATCTGGCCAGAACATG
TACAAATTGGACACCATGTAGTCAAATCGCGTTAACTGTAACATATAAGATTGGCTAACACAGCCAAT
CCAAACCAACAACAGCTCTAAAGTACCATAGCCCACTGGCAAAAGGGAGATGTATCGAATGATTATA
AACTTCCAATTGTGTTATTTGTGCTATTGCTCAACCAGGAGATTGGCCCTTTGAACCCTCTGGATGCAT
CACTGTATATAACAATCAGATGGTTTGAGCATAGGTGATAGAATCCTGACCAAAAGGTAAAGAGAG
ACATAGGAGACCCACAAAAATGAACTCATGGATGGATTGTGTTACACATGCCAACATCATGTGAGAA
TCCAGCTGTTTGAACATAGGCACGTTGAACTCTCTGGATACATCACCATATACATCAATCAGATGGTT
TGAGCAGAGGCACGTTGTTAAATAGTACCCAACACAAATCCTGACCGAAAGGTAAAGAGAGACATG
GGAGACAGACAAAAATGAACTCATGGACATGCCAACATCGTGTGAGAATCCAGCCGTTCATCAGTG
AGGCACCACTTAGAATGCTCCCTGGCTGAAAAATCAGGCCAACCTGCTTGTCAGCCACGGGTGTATT

TTCAATGTGCACAGTTAGGGCAGCCAACAGACTGGGCTTGGGGTCGGCTTCAGATCGGATTTTGAAA
TTTTGGGCCAGGACAATGTTTTAAATAGTGAAGAGTGTAGCGTAGCCTTCACCCCTCTGAAGCGTGA
GGCATAAGCTACACGCTACTTCTAGATTTTTAAACAAGGCTATGCTTGGTTGCACCAATTTCATGATA
TTCTACACCAAATTAGATTGACTAATAATAAATTATAACAAAATTTCATGATTTTGGTGCAACCAAACA
CAACCGAAAGTAATAGCAAGGGGTAATGAATAAGTATAAAAGTGAAGAGAGAGCAACAAAACTGA
TTTAAAACTGTTTCTCACATTATTTTACTAAAAGCATTGAAAAGATTAACGAATTTTCATTGAAATAGA
AAATTGTAACAAATCATTAAAAACATTATTGATTTTAATGTTATAGTGAAAAATACCTTGTAATGTGA
GCTATGCAGCGTGGTTTTTTTTTTTTTTACACGCGCACTTTACCCCACACACATGCACTTACACCGCAG
TGGGATTTCACCATAATGGGTACTCGAACCCATGACCTCGTGTTGAAACTCTTATGAGTCTACCACCG
AGGCATGAGTAAGGACCCAAGCTACGCAGCGTGTAGCGTATACAAATTAACCAAGTAGCATAGGCT
ACATGCAACTTAAGCTATTTTCATGAGCTTCATAGCATTTAGGCTAAGCTATGTAGTGCAAGCTACAC
GCTACATAGTGTAGCTATTTAAAACACTGGACTGGAGCTATTCAGATTTTGATTTTGATTCACCAGGA
CCTGGCCCATTGAGAGCACAATGCACGGCTGGTAAACTTGGTTTAACCATCCGAATTTTGTGCGTGA
GTGAATGTTGCCCACGTAAGTGGACTGCCATAATTTTTTTGAGCCAGTCGATATGCATGGTAGCCCCA
CAAAGAATGGTGATGGCCTTGTTCACACGTTACATGTTGGCACATTTGGGGGTACGCAATTGGAATG
CAGACACAGGAATCACCAAGCATGGTCTAGAAATTTGTCAGAAATGACATTTGAACCCACACCCTCT
CTAGGACCAGAATTTGAGTCTGGCGTCTTTAGACCACTTGGCTATAGCATGGCTTATAAATTAAATTG
AAACTCTAAAATAATCTCCAACTGGTGGGTTTACGAGCCCCACAGATCACATGTATCAAAACAAAATT
CATGCCCATCCAACATGCATTAGCAAATCAAGCCCAACCATCTGTATGTTTTTTAATGATCTGAAGTG
TCCATCTAATGGGCATGACCATGGATGGGATGCCCATCCAACATGCATTAGCAAATCAAGCCCAACC
AGCTGTATGATTTTGGATGATCTGAAGTATCCATCTGATGGGCATGACCATGGATGAGATACAGCCA
CGAACTCAAGCTGATTGATGATCCTGACCACCCACTTGGTGCTAACTGGAGGTTGAAGACAAAACTA
ATATCAATCTTCGTTCACTTGGAAAGAATTGTGTATTAAGATGTCAAGCCATTGTGATTTGGAACTAC
ATCCCATCTGCAATTGCTCCCACCAATTGGACCCTTTGGGTCATCCAAATTTGAAGCCATGAAGGCCC
CATCAACAAAATTGCTGGCAGGTATTAGTCCCCATTCCCATATCACAGGTGCCTTAGGAATGATGCAG
TAGAGCCCAATGTTCCAAACTCGGTGGTATTATTGATAATATAACCGGTTTTAAAATTTTCAAATATC
ACTGATATTTTTTATAATATCAGCGTATTATCGATATCAACGTTTTTTAAGATGATATCAGTGATATTTT
GCGAAGAATCCCTTATAAAAGCTCTCTAGTATCTGCTATAATATCTCCAATACCAACGATAATATCCCC
GACATTGGGGAACCATCCATAAATAAGCAAATTTTGATTTTAAAAAATGGACAAAAAATTCTAAAAA
CTAAAAGATCTCCTTTTCTTTTTGGAATTTTGTTCCTTAGTGATTTTGATCCCTCTTAGCTAAATCAAAG
TTTGAATTTGATGAGAAATCTTAACTCGGAAAGAAGATAGGCCAGAACTCAGAGTCAAAGGTGACA
AAAAGTCCATAGAAACTTCATTTTTTTCAAATGTTAGCATGGATTGCAATGGATATCCATGTCTATGC
ATGATTCTCATACATTGACATGGATTTGTGGTGAAAAATTAACATTTAAATGAAAATAGGGGAACCA
TGGTGTCCCATAACGGTAACGGTGGCCGTAACGGCCACCACCGTTATCATTACGATACGGGCCGTAA
CGGACGACGTTACGGACCCTTTTTTATTTTTTGAAAAAAAACTGTTATTAGATCGTTACAGATCCGAT
ACGGGCCGATACGGGCCGTTACGGCCTGTTTTTTCTGTAACGGCCGTTACAGCCGTTTCGACCTTGTA
ATGTGTAACGGTCATGACCGTTTCCGTTACGTATCGGCCAATACGGCCGATACATAACTGTTTTGGGA
TACCTTGAGGGGAACTGGTGAAATCTTAATTTTTTTCATTTTTATATTAGAAAATGTGTTTTTCACTGT
TAATTAATGTGAAAATTTTATATATTACTAAGTATTAACTAATCTAACAATTGGCTACAAATTTTATAT
ATTAATGAGTATTTGACTGATCGAACAATTGGCTACAAATTTTATATATTTATTTTGCACAGATTTTTT
GATAACACATGGTGAGGTCCCTATAACATGGAAACTTATTATGTATAGTCTTCTTTTGCAATATTTTCA
TTCCTACATGTGTAAACATGTGTCTTTTTAAATTTCATACCAAACCGCGAACACTGTTAGAACCCAAGA
AGAAGTGGCGCCATTCGGGATCATCCATCAAGTGACGCCTACTATGGACATGAAATGGTTCAAAATA
AACTGATTGGACCACTCTAGCCATCAAACTAGTGGCCTAATGGTCAAGGTTCAATGTGGATGAGTGC
AAGAAAACTGATGGCTCGAATTGCCCCAAAATTCTGGTATTTGCATTGTGGTGCATGTCTCCAGCTG
GATAACGCAGCTGGTCATGATGCCATCTCACCCCCTACATTACCCGGTCAGAGACCTAGTGCAGGTC
ACCATCTGGAATAGCGGAGCAAGTTATGGACGGAACTGCGCGAGCCTAACAAGGCATAACCAGACA
GGCGTGCCTACTACTAACAAACGGCATGTGGGGGAACTCATGCATGAGATCCACCCTGTTCATCAGG

TGGCTTGCCTCCCATCCAAAACTATGGTAGGCCACTGTAGAGAATAATTGGGATGGGACGCCAACCA
TTAGTTGAGTGTAGAGCCAACTGTGGTTTATGCATGCTATCCAATTTGTTCATCTAATGCGAATCACC
AGCATGAAGGAAAGATTCAAAAATCACACTATTCACCAAAATCAGCTGGGCCATACCAAGTTTTAGG
CATGATTTTACATTGTTTCCTATGTTGAGTACCACCTAAGTTCAAATCAGTCTGATGTTCAGGGTCAAG
ACCTAATGGATGGTGTGAATCTCGTGCACGTAGGGGTGAAAATGGGTCAGGTCGGCTGTGGTAAAT
GCAGTGTTTTAAATAGCTATGCTATGTAGCGTGTAGCTTACGCTACGTAGCTCATGAAAATAGCTTAA
GTTGCGTGTAGCGTATGCTACATGATTAATTTGGATACGCTACACACTACATAGCTCACATTACAAGT
CACTTTTCACTGTAACATTAAAATCAGTCTCTTTCTTCACTTTTATACTTAATCATTACCCCTTTCTATTA
CTTTAGGTTGCATTTGGTTGCATCAAATATCATGAAAGTTTGTTAAATTTTATCATTAATCATCTAATTT
GGTGCAACCAAGTGCAACCTTGATTAAAAATCCAAGTAGTGTGTAGCTTACGCTACATGCTATGTAG
CTTACGCCTCACGCTTCAAAGGGCTGAAGGCTACGCTACAAACTATTCGCTATTTAAAACACTGGGTA
AATGGTAGGCCTGGACCCAACCAATTTAAGAATCGGGTCAGACTTTTGGACCTCAACCCAAAACATT
ACAATTCAAGCCAAGTTTGGTTAGATCCAAGTAAAAAATAGCCAACCCATTTACTTACAGCCTGTTTG
GAATGTGGAATTAGTTGGGATTGGGTGGTATGGAATTGCATTTGGTCTCATGTAAATTCCATCTAGC
CTTTGGAAGTGATGGGTAGTATTGGATTAACCGAGGTATTTGACCTTCCAATCCCACCATGAGATAA
CAACAGGATTTCTGGTGGAATTCAAAATCCACTACATCTGTCGGCCCCATGTTGATGCATGTGTTTTA
CCCATGGTGCCCATCCATATGCACACTTTACACATGAAACACAAGTTGCATATGAATATAGGTGACCA
GCATCAGTTGTGAAATCTAGTCAGTTAATTACAATTCTAGCATCCACCAAATGCTACTTTCACGTAAA
ACATTTTTTTCTCGAAGGAAGAATTCAATCCCGAATGTGGGATCAGATGATCAAAATACCATGATATT
TCCAGACAAGTAATCATTGTGTGATAATGGTTTTAATTCCATCTAATGCAATTCAATACCAACTAATTC
CATGTTTCAAATACGCCCTTAATAAATTGGGACTGGCACAAAAGGGCGAGGACCTGGACCTGGACCT
GGACCCGGACCCGGACCCAATCCATTTAGTACATGGATCAGGTTCAGATCAAATATAAAAATTATAA
TTGAGTTTCATACAAATAATATTCCAAATAAATTCAATTGGTGGTGGGTGTCAAAAAGTGAGTATCAA
TTTTGATGGTAAAAAAATATAATGGCCAATGGTCCAAATTCAACAAGTAAAATAAAATGGCTATGGT
AATCCAATTAAGGTTATTTTTTTCGGTATCTTCCCCATCAATAATGGGCCCAATATTTGCAGTGTGATT
GTCATAAGTAATAGATGGGTAACTGAAAAAGATGGTGAACCATTGTTGAATCTACCAAAAGAAAAA
ATAAATAAATAAAATAAAATAAGCCAGCATGCTCAGTTAATTACTTTTTCTTGGGTCTAGGTGGGATT
TGGATTCAGATCATGTCTATTTTAGCATCACCCAATTGGATCCATTTTAAAACTGAGTTTGGGTTACTT
AGGTCAGGTCAATTTTGAGACAACCTTGACCTCACCCATTTCGAAATTTGGGTTGAAGTTTTGGACCT
AGACCAGCCCCATGGGTTGAAGTTTGACACCTACCAATTGTCAGGTCAGTCAGGTCCCCCCAACCTG
CTTGCACCCTTACATGCCCATGAGGCCGCTCCCCCCACACAAGTGGAAAGCACGCCTTTGTCTAGACT
ACTCCAGCATTTTCCCCATATGGCAGCCATCGAGAGTGGGGATGGATTGCCTGTGACTCCTTGCACA
GATATATCTCTGTCCTCAGGGTTGCGTGGGGTCTATAGAGATGTCCGTGATAAATCCACTCTGTCCAT
CTGTTTTGAAAGACCACAATAGAACAGGATTCTAAAAATCAGGCAGATCCAAAACTTAGGTGGGCCA
CACCGACAAAACAGTGGGAACAGCAACGTCCACCATTTAAACCTACCTGAAGATGACCATGATGTTT
ATAAGCCACCCAAACCGCTCATAGAATTATTACCACTCAGATAAACTCTAGCGACAAATATCATCTTG
ATACAAAACTTCCATCACTAACAGGTGTTCAATTCCCACTGTCTCGTGTGGCATGGGCCACATAAGTT
TTGGATCTGCCTGATTTTTAGAATCCTGTACTATCATAGTTTTTCAAAACAGATGGACAGAGTAGTTT
GTCAAGGACATCTCTGTGGACCCCACACAGCCCCAGGCATAGAGATACATCTATGCGCGGGGTCACA
AGCAATCGGCTCCCATTGAGGGAGCTCCAAGGAATTCTGCAGTGGGTCCCACCCTGATATTAGCTGG
ACTCAGATACCAAAAAAACATTGTGAGGCATTTATGTGAGGGACCGAGGACGCCATCCAGCGTGTTA
CAGGGACTTTGACAATGGGACACAGCAATCACTAGCTTGGACCAAAAACCACCTAACCCTTCATTAG
GTGACCCGCACTCACACCGGGCCTGTTTGATTTTCCAATTCCTTAGTAAATACCAAGGAAATAAGTCA
TTATTACTTTCTCACCCGTTTGAAAATTCCATGTAATTCTTGCTTTGAAAACTAATCCAAAAGTAGTTTA
AAATTGTGGGCCCCACCATAATATATATGACAAATCTACACCGTCTATTAGTTTTATCAGAATATTTTG
AGGCATGAGCTGAAAAATGAGACTAATCTAACACTCAAGTGGCCCACACCAAAAGAAACATTGGCC
CCACGAAGTTTTCAACATAGATATTCAATTCTTTTTTTTTATGGTGTGGTCCACCTTAGCTTTGGATTTG
CGTCATTTTTGGCTCATTTCCTAAATTCATCTTGGCAAAACAAATGGACGGTGTGGATAAGCCACATA

AATTATGGTGGACCCCACCAATATTTCACACTCTTAGCAATAAAAAAGAAAAGTTGGCAGCAGTCAA
ATCAGGCACAGGGAACAGTGCAATAATTACCCAAGGAAATAACCATTACGCATATACAATATATTTA
CCAAAAAAATTGAATCTCTGATAAATACATTGTAAATATATAATGATTCTCCATGCATGATTTGACTGA
ATACTTTTTTATTTGCTAAAATCGGGGAGAACGACAAATATTGGTGGGGCCACTGTGATATATGTGG
CTTATCCACACTGTCCATCTGTTTTGCCAAATGAACTTAGGGCATTGTGGACCAAAAAATAAGACAAA
TACAATGCTTAGGTGAACCACACTTCAGGAAAAAGGGAATCCAACACCATTAGTTGAAAACTTCTTA
AGGCTACTGTTTCCTTTGGCGTGGGCCACTTGAGTGTTGGATCAGCCTAATTTTTTGAATCATAACTC
AAAATATTCAAGTAAAACTAATGAACTGCACGGATATATCATATATGTCATGTGGGGCCCAAAATTTT
AAACCGCTTCTTTTGAACCGGTTTCAAGGTAGGAATCAACCGAATTTTCAAACAGGTTAAAAATGTAA
TAATGCCTTATTTCCAGGGTATTTACAAATAAAATGAAAAATCAAGCAGGCCCTTCTTAGAAAATGGA
CGGTTAGAAAATCACCACCGGCCTGTGTGGCCAATGACCATACTCAAGTGTACCATCGTCACTTTTGT
AACGTGGCACGTCCCAATGGAAACCCACAATATCCCGGAACATGCACACATGTCCACGATGGCACGA
GCGAAGACAAGACCTTTCCCGCGAAGAAAAGACGCTGCCGCTCACATCCGCCCATGTGTCAGCACTC
AGCAGTAGCTCTCAGTTTGGTGGCGTACTACGCACTACGTGTACCTTACTCTGACGCCCGCCCCTAAA
TTTGGACCAACACAAAAAATGCAGAACCGAATTATCTGTCCTCCAAAAAATAGAAAATAAACAGAAA
AAAAAAGTCTCAGCCCTAAAAATTTACTACCTAAACCAGATTAAAACCCCTAATCCGGATCTAATGGA
CCCAAAAAAATAACTAGAAATAAAAAAATGACTGTTATTCTGCCCACATTGGATTGATTTCTCACAAA
AGTGTACTACTCTTATTCCAAAATTGAAAACTGGGTGTGTGCGGATAAAAATAGTAGGTATAGAAGT
CAGCATCTATTTTACCTTCCCATCGTACTCGCGTCGAGAAAGTACTTCAGGCGCAATGTAAGCGGGC
GTTCCCACCGTTGATTTGGGTCGGGAATGAAGCAGAGAGGACTGAAATTTCAAATATAAAAGGAGT
GTATTTCAATTTTATGAAGTTTCTGTTGGAAAAAAGCTGATATTTCCACCCCTCAGAATTGATTGGAC
AAACCCCACGAGAAGTTTGGTTGTCCATTGAAAAGTTAGCAGAAACTCAAAACCACCTAGATACCAT
TTTTGGGGGGTGAAAGTATCAATTTCAGGGGGTCTTTTGTAAAAGCACCTTGGAATAACCAAAATCG
CAGATTTTCAAGCGCGGAGCTGGGCTTCCATCCAACAGAGTATTCTCCAGCTTCAAATCTCTATGGCA
AATTTGCTGCAAAATTTTAAAAATAAACAATTAAAAAATTAAAAAGAAAATTTCATAAATCAATGAAA
AAATTTGATTTTTTTTTTCCATTTTCTCTGATTGAAATTACCATTGAATGACAATAGCTGACTCCTGAAA
TCAGCTGCTGGAAAAAATACCTTGCCTGAAAATAAAAAATAAAATTCAAGCACCAAAAAATTAACAA
AAAACAAAAATCTGACTTTTTAAATTCAAAAAATAAAAAGTAAAAAACCTCATCTTCGCTGAATCTCC
CAGCGTTACAGATCCGCTCGAAAAGCTCTCCCCCTGCAGCATACTCCATCACAATCGCCAGATGCGTA
GGCGTTAAAACCACCTGCAAAAAAAAATACTAAAAGAAAATTCCAGGAAAAAAAACCCAGAAAATT
GATAGACAACACCACCCAAAACTAATCCCAAGGTATAAATCACACCTCCTTGAATCGAATTATATTAG
GATGGCGAAGCGATCTGTGATTTATAATCTCCCTCGCCACGTTCTCATCAATCTGCAAAAATTAGAAA
AAAAAAAAAAACCCAAATTCTCAAATCTCTCAAAAAACCAGAAAATGGATTAACAAGACAGAAACAA
TACGAAAATTCGTTTTTTTTTTTCAGACCTTGTGGCCTCTCTCGATGTATTTCATTGCGACGAGCTCTCT
CGTATCCTTATTTCTCATAAGCCGGGCGACGCCGAAATTCCCAGATCCGATGTCTCTCACCAGCTCGT
ATTTCTCCAT

>Lchi12999

TCACATTGCGTAGATGATCTCCCCACTGCTGTCGACATCAAGTTCAGGGTCAGACTCTAAATCCTCCA
TGTCATCGTCGAGGTCCTCACCTATCAAAAACTGGTTGATGCTGCGGGTCCCAGCCGCAGGTATTGT
GGCTTCTGCAATGATCTGCATGATCTCGTCAATGCTCTGCATGGGCTGGTCAGGCTCTTCAAATTGGT
TCACCGTGTTTTCGTCCATAAGATCTGCTGGGAGGTTCTTTAGGAACCACTCGTGGTTTCGGATCTCC
GGAATGGTTATCCTCTGTGATGCAAGCAATATTCAAAGATGATAATCAAAAGAGCAAAACTTTCTGA
GTTCCAACTTAACAAAGCGGGCTTAGCATTTGAGGCTGGCATGGAAAATGGAACCTGCATTAAGCAA
ACATACTTTCTCGTCTTCTCCGGGTTGGTGAGAATCACAAGTGAGATATATAGGAAATATCTCACGAT
CAAGATATGCAATGACATAATAACTACCGTTTAGGTCTTGGGAACAAGGAATTTTCAAAAGTGGACT
AAGGGCCAGTTTTCTAGAGATGCCAAAAAATGAATGATGAGGACACAAGACCGTTTAAAGTCTATTA
GTGAAGAAAGCGCACGCCACAAGCGTCTTTCTAGCGGCTAGGCGACTATTGAGGAGCATTGAAGAC

CCTACACAAGTGATTTGACACATTGAAGGCCCCACATTGGGAGGACGTGTGTGTAGGAGGAAGAAG
ACATGCTGGACAGTTTTCACTATCTTCGGGCGCTCGATCGGGCGCCCGACCACTGTCTTCAAGCGCCA
GAAACTTACTGGACGTTTTTGGACATCTTCGGGCGCTCGACCGAGTGCACGACTACCAGTGCAAATT
TTTTTTTTTATAATTTTTGCGAACTTTTCAAGGGTATTTTAGTCCTTAGTTAATTTATATTGCTTATTTAA
GTGGGCTAAGCTCTAAACTCAAATTAGTTGATTGATTAATGAAAAGCAAACCCTTTGGTTGCCTCCTC
TCTCTCTCTCTCTCTCTCTCTCTCTCATTGAACTTCATCATTTTCCTTGATCTTCTTCTTCTCTTTTCTTCTT
GTGCCCCTCCAACTTCTTCCAGGTAATCTATCTCTTATTCTCATTATTCTAAATCCTTGTTTTAGTTAATT
CCCCACGACCAAAATCTGAGAATTAATCCCAACCCTAATTCCTCAGATTTTCAAATCCCTAATCCGAGA
AACCTTACGATTCCTCGGACTAGTGATCTCTATTGATCGATTGGATTAAGACTATGCAAGATCGGGA
GGTTCCATCCCTCGCCAGATCCAACCTAAGCAATAATTGAATTTCACACTTCGTGGTTGTGACGATGG
CCCGCTCCATTGCATGTCTCTTTGTGATCTTCTTAGTTCATGTTGATTGATGCTAGAATTTTATATTATT
ATTGTCTTTTATTTTCGCTGCATTATATGTATTCATGAGCATGCATCATATTTAAGGCATCCTACGTCA
AATTTGGTATCAGAGAATAAGCATGCATGGTTTTGTCTAGATCGAGTTATCAAATTAGGGTTTTGATT
TTTAGGTCTGTTTTTGAAAATTTTCAGATTTAAAAAGTTTTCAATTTTAGAAAGTTTCCTAATTTGGAA
AATTTTCAGATTTGACTAATACTCTGTTTTAATTCATTATTTGTCAGTTCATACTAGTTTTGCATTCACC
GCATTCATCTTGTGAGTTGTAAAACCGTAGGAGGGCAAGTTGGGTAGAATTTGGTTTGATTTTTAATT
GTATGCCCATGAAAAGAGGTAAAGGTTTCGGACTTCAACCAACCATGAATAACGAGCAGTTATCTGA
GCAGCTTGCTCAAGTGATACGAAAGATAACCGCTTTAGAAACGAGTCAAAGGCAAGAGTTCGCTCGT
ATGGACAACCAAATCGCAGCTACCATGACTAAGGTCGAGCAATTAGAGACATCCCGAAAGGAGAAC
CCTACTATTAGGGATAATGCGCACTCCCAAGTGAGAGAAATTGTTGAAGAACGCACTCCCACACATA
GAGATAATGGTGGTAGAGATCATGGAACGGGTCGTGGTGTTGTGCGAGAAACACAAGCCATATATG
ATCATCAAGACCGCTACGATCCAGATGAGCGTGCGATGAATAATGTGAGGGTAGAAGCCCCAAGTT
TTGATGGGCGTCTGGATCTCAAGGCATTCCTTGACTGGGTTGCTGACATAGATCACCACTTCAAGTG
ATATGGCATGTCAGAAAATCGTCAAATGCGGTTTGCAAAAATGAAACTTATGGGCCAGGTCAAGTTA
TTTTGGACCAACACTGAGCGAAAGATAGAAAGAATTGGTATAGCCCCTATCACATATTAGTATGAGA
TGAAAGAGAAGCTGAAGGAAAAATATCTTCCTCTCTCATACCGACAAAAGCTCATTGACTAATGGCA
ATCCTTACGCCAAGGTTCAATGTCTGTCACTGACTACATCGACAAATTTGAGGAATTTATTATGCGAT
GTGATGTCTAAGAGGACCCTCTAGTAACGCTCTCACGTTTCAAGACGGCCTTCGAACGAATCTTCAAT
GCGAGCTTATGACTCGGCCCATACTGGACTTCGATGAAGCGTACCAAGTTGTGCAAGAGTTGGAGC
AATACTTGACAACTCCTATCATTCGATGTTTTGAGTCCCGAGACTCTAATGCTAGATCTGGTTCCCAG
AGAACTAGACTTAATGTTGGTACTCGACCTAGGGCGCAGGCGACTGTTTCGCCTAAGCCTAAGGATA
ACAAGGGCAAGGGGGTTCTTGGTGCCAATCCTAGTGGGGGTAGCCAAGTGAAATGTTATGCGTGTG
GGAATATCGATCACATATCCAATCAGTGTCCCACGAAGAACCGTGGAAAAGCCTTAATTATAGGCAA
ATCTCACGAGGGTGTTGATGACCAGGGTGATTATGAAGTAGAATAATATCACCCTGAGGAGGTCTTA
CTGATGAAGACAAAGTGGGCGATGAAGCAACGCTTGTAGTAATTAGGCATGTGCTATCTCAGTTAG
GAGTAGTGTTGATTGGCGTCGAAACTCTTGCCAAGTGTGGAGAGAAAAATTGCTAGGTAATAGTGG
ACAGTGGAAGTTGTCAAAATGTGATTTCCGCTAACACCTTGGACCGTCTGAAGTTGAAATCCACACCT
CATCCTAACCCATACGAAGTTTCTTAGGTTGACAAAACATCCATTCCTGTCACCCATCAATGTTTAGTT
CCTATCAAGTTTGGGTCGTACAAGGAGTCCCCATAGTGTGATGTGATACCTATGAATGTTGGACATA
TTATCCTAGGTAGACCGTAGCTATTCGATAATGACGCCACGATCTTCGGTCATTCTAATGCGTGTACC
TTCATGCATAATGATAAGAAGATCAAACCTGATCCAATGCCACCTGAAAGCACCTCTGGGAAGAAAA
AAGATGAGAAGGCGAGCGAGCCTAAGGAAGTAGGGAAATCCAAACCTAAGTCTCTTCATATAATCA
ATGCGAGAGAATTTGAGAAAGAGACTAAGGAGGATTCCATGGTGTATGCCCTTGTGGCAAGAGAGA
TTACACCCAAGGTTCCAGCAGAGTTACCCCATGAGGTAACATCGGTTTTGAAGGAATACAGTGATGT
GTTCCCTGATGATTTACCGAATGAGTTGCCACCTACATGGGACATTTAGCATGTCATTGACCTCGTCC
CGGGGTTGACTCTACCGAACCTTCCTCACTACAGGATGAACCCTGCAGAGCATGCAGAGTTGAAGAG
GCAAGTTGATGAGCTTTTGCAAAAGGGTTTTATTCAGGAAAGTATGAGCTCGTGTGCTGTGCCCGCA
TTGTTGACGCCAAAGAAAGACGGCACGTGGCTCATGTGTGTGGACAGCTGTGCCATAAACAAAATTA

CCGTCAAGTATAATTTTCCTATACCTAGACTTTATGATATTCTTGACATGATGGTCAGGTCCACAATTT
TCTTCAAAATTGATCTCAAGAGTGGATATCACCAAATCCGCATACGCCCAGGAGATGAGTGGAAGAC
GGCATTCAAAATGAAAGATGGGTTGTATGAATGGATGGTCATGCCCTTCGGCTTGACTAATGCACCC
AGTACTTTTATACGAGTGATGACACAAGTCTTGAGACCGTTCATGGGTAAATTCCTAGTGGTGTACTT
TGATGACATTCTTATTTATAGTACCACGAAGGAATCACACATGAAACATCTGAAGCAGGTCTGTAGC
GTCTTTAGGACTAAAAAGTTGTACGCTAATCTTAAAAAATGTGCGTTCATGTCTAATCGAGTCGTGTT
CTTAGGATTTCTAGTGTCATCAGAAGGAATGCGCGCAGACCCTGAAAAAATCAAAGCCATAGTTGAG
TGGCCTGAACTGAAGAACATTCACGAGGTGCGAAGCTCCCACAGCTTAGCAACTTTTTATCGTCGATT
CATTGGGAGATTTACCACAATCATGGCTCTCATTACTGAATGCATGAAAAAGGAATAGTTCGTGTGG
TCGAAAGTCGCAGTCAAGGCTTTTAAGGAAATTAAGGGCAAAATGGTAGAAGCTCATGTCATGCGTC
TACCTGACTTTTCTAAAATCTTTCAGATGGTGTGTGATGCGTCTGGTGTTGGTATAGGTGGAGTGTTG
AGCCAAGAGAGTCATCCAGTAGCCTATTTCAATGAGAAACTAAATGAGGCAAAGCAGAAGTACTCC
ACTTATGACAAGGAGTTCTATGCGGTAGTGCAATCCCTGAGACATTGGCGTCACTATCTCCTACCGCA
AGAATTCGTCTTGTTTTCAGACCATGAGGCCTTAAGATATTTGCATTCTCAGAAGAAACCCAACCCCA
GGCACGCGAAGTGGGTAGCGTTTCTTCAAGAATATTCGTTCGTCCTGAAACATAAGGCCGGGGTCG
AAAACAAACCAGCAGATGCCCTTAGTAGAAGAGTAGCGTTGCTCAACTCCTTGACTGTAGAGGTAGT
CAGATTTGAGCAACTGAAAGATAAGTATCCCATATGTCCTGATTTTGGGAACACTTATGCGTCACTCT
CTAGTGATCAGCATAGCACGGGTGATTATGTGCTTAAAGATGGTTTCCTTTTCAAGGGAGATCGACT
AATTTTTTTCCTTTTCAGTGCAAATTTTTTTTTTATAATTTTTGTGAACTTTTCAAGGGTATTTTAGTCTT
TAGTTAATTTTTATTGCTTATTTAAGTGGGCTAAGCCCTAAACTCAAATTAGTTGATTGATTAATGAAA
GGCAAACCCTTTGGTTGCCTCCTTCCTCTCTCTCTCTCATGGAACTTCATCCTCTTCCTTGATCTTCTTCT
TCTCTTTTCTTCTTGTGCCCCTCCAACTTCTTCCAGATAATCTATCTCTTATTCTCATTATCCTAAATCCC
TGTTTTGGTTAATTCCCCACGACCAAAATCTGAGAATTAATCCCAACCCTAATTCCTCAGATTTTCAAA
TCCCTAATCCGAGAAACTTTTCAATTCCTCAGACTAGTGATCTCCATTGATCGATTGGGTTAAGCCCAT
GCAAGATCGGGAGGTTCCATCCCTCACCGGATCCAATCTAAGCAATAATTGAATTTCACACTTCGTGG
TTGTGATGATGGCCCGCTCCATTGCATGTCTCTTTGTGATTTTCTTCGTTTATTATGATTGATGCTAGA
ATTTTAGATTATTATTGTCTTTCATTTCTGCTGAATTATATGTGTCCATGAGCATGCATCATATTTAAGG
CATCCTACATCAATGAGTTAAATCTCATTGTAGTTAATCATAACTATGTACAGCAGATCATGATCTCTA
ATACATCATTCCATTTAGTTACTCATAATTATATATTAGAGATCATGACCTCTAATACATAACCATGAT
TAACTAAACTGAGATGAACTCATCTTCAGGCATCTACCACACAGCTAAAGCCATCAATGGGTAACCG
GACCCACGAGTACCCGATGGACCCAAACTGATTTTAACTAGAATGGGTCTAATTTTTTAGACCCATCA
CGAAACCAGGTCGGGTTTTGAGTCTAGCCTTTTAGGATAATTAAATTTGGGTCTAGCCAGGTTTGGG
TCGGGTCCATCAACTTGGACCCAATTAAAATCAGGGTCGGGTTGGGTCAAATCGAGTCTGGTTAGGT
CCGATAGTTTTAGCGGTGATATGATGTATATTGTTGTTCAATCTATCCCCAATATCATCTGTATCTCCA
GCTCAGCGATACCGATAACACTACTAGTATCAGAAATTTCAGGTATCGGCAATGTGTCACTAAATATC
ACCAATATTTTCAATTGTGTAAATTCCAGGTGTTGCTTGTATTTACAATGTATTGATATCACCAATGTA
TTGCCAATATGTTTATTTTAAAAAAAATCCATTTCAAAACTTTTTTTAGAACATGGTTGTATGTAGTGT
TCAATTTTTCAACGATAATATCGATAATATTGTGATATCATCGTTATCGCAACATGGGCGATATCAAA
ATCCACAATCTTCCATTTCCTTTCCCGTTGTGGATGATTTCTCAGTGAAATATCACGTGTTGTCGATAT
TCTAAAATATCGACAAATGTGTGGATGGAAGATTGGATGGTTGAATAGTTAGATGGGATGGTTGGA
TGGATGGATGGGATTGTTCGACTGATTTCTTATAACAGCACATGCTTTTGAGGCCCCCCATTAAATGG
AAACTTACTATGTATGCATTTTTTTTGTAATTTTTTTATTCCCAAAATATGCAAATATGTGTATTTTAGC
ATCTTCTTAAGTTTCATAGAAAAATTACACCATTTTCTCCATGTTTCTCCAATGTTTCCCCGCGTTTTCA
GTTATTGGCGATATCGATATTTTTTCCATATCCCTAGCCAACAAAACTTGTAGCGATACCAATAATTAA
CCCAACAAGGTTCGCTAAGCCATACACGTGCGAGAGCCCATATACATACCATGCATGTGTAAAAGTG
GCATGTATACGCTCAATCTAATCCATCCATCGGGATTTTGAAGACCTGACCCAATGGGTGCCCAACAA
ATACTAGAACTCGATCGAATCTAGGTCCAGGTCGAGGTCTTCAAGAAAACGACCCAGACCTAAAAGG
ACCCTGACCATACATGACCTAAACCCGGTTAGCCTGGGTACAGCTACTATAGGACCTAACTCGAACCC

AACCCGTTGACAGCCTACAAACAAGCCTTAAATGTCTAGAGCACCCACCTTTTCAAGGAAGCATTTTA
GTAGGTATCAAAGAAGATGCTTTTGCATTCAATTGACTATGCTCAATCAAGCGTGAAAATATAGAGG
AAAAATAACATATATATAAATTTTCAAAAGTAGAAAAATTGCCCATAATAACAAGACCTTGGAGCAG
AGCTATTGTTCGTTTTGCACAATTTAAAAAGCAGTGGTGACTCATCCTCCCCACATGTGGCCTACCCA
GACCATCCAAATTGTGGGTCATGTTGTGGGTGGAGCATATCTCCAAAATCTCACAAATCAAGCAATC
CATAACATCCGATCAATTGCTATTGAATACATGGTTAAAAAAACTTAGTGATTAGATCAATTTCGCAA
GGAAACATCATATGGTTTTCAATGGGTGTTTATAGCTATGTATGGGCCATGTGTTTATAGTTTGAGAG
ACCAGTTATGAGCGGAGCTAGATAGTGTAAGGCAGAGGTGGGAGGTGTCGTGGTGCGTTGGTGGG
GATTTTAACTGTGGTTTGGTTTTCCTATGAAAAATATAAGGGTGGGCGTATAACCAGAAGTATAAAG
CTTTTCTCGAACTAGGTGGCTTACAACGAGATGGATGAACTTCCTTTGGTGGGTGCCAGGTTTACGT
GGTCCAACAGGCAGGATAATGCAATGTTATCGCATTTGGACAGATTCTTAATGTCTGCAGATTGGGT
TGAGAAATTTCCTTTGGTTAGTCCACGCAAATTGACAAGAATTGCTTCCGATCATAGTCCAATTATTCT
GGAGGTGGTTGAAGACAATTGGGGCCCTAGACCCTTCAGGTTCAAATTGGCTTGGTTGGAAATTGAT
GACTTCGATCAGCTCGTAGCAAATTGGTGGACTTCTATCTCGATAGAGGGTTGCGCTAGCTTTAGATC
GAGTAGAAAGCTTAAGACATTGAAAGCTAAAATTAATTTGTGGAAGAGAGAGGTGCTTGGTGCTAT
ACCCTTCAGGTTCTTAATGTCTGCAGATTGGGTTGAGAAATTTCCTTTGGTTAGTCAGCGCGAATTGA
CAAGAATTGCTTCCGATCATTGTCCAATTATTCTGGAGGTGGTTGAAGACATTTGGGGCCCTAGACCC
TTCAAGTTCAAATTGGATTGGTTCGAAATTGAAAGCTTCAATCAGCTTGTAGCGGATTGGTGTACTTC
TATCCCAGTAGAAGGTTGCGCTAGCTTTAGATTGAGTAGAAAGCTCAAGATGTTGAAAGTTAAAATT
AATTTATGGAAGAGAGAGGTGCTTGGTGCTAGGGAGGCTAAATTCGATAAGATGGTGGAAAAGATT
CATGAGTTAGACTAATTGGAAGGTGGTGATCTCTTAGAAAAAGGTAGAAAGAGGAGGGCTAAGTTA
TCTTTATCTTTCGCCAGCCATTCGAAGGAAGAGGAGATAAAATGGCAGCAGAGATCTAGAGTGTTGT
GGCTCAAGGAAGGGTATAAAAATACGTGCTTCTTCCATAGTATCATTAGTGCCCGAGCTCGTGAGAA
AAAAAAAAAATCTCTAGTTCGGTTGTGGATGGGATAATGATGCAGGACAACTAACCACTTGCTCTAA
AAGCTCGAACTGTTAGAATGTGGCGAATCAATCCCTTTATCTCATAGCCCAGGTTGAGAGGGCCCCG
AATCCACGGGTTCACCTCAGGGCCCCGAATCCACAGGTCACCCCAGGCCCTCGGTCGAATCCTCCTA
CGTGGGCCCCGAATCCACGAGTGGGTCCGAATCCACGGTCCCCCTGAGTCCACAGGCACTGAGTCCA
CAGCGGACCCCACCCCGAATGTGCTCCTGAGTCCACAGGCCGCCCCACTCGAGCCCGATGTGAAAAT
GCCCCTGCATTAGATAAGAAGCTCGAACAAAAGCAAGATTTGCGACTCTATAGTTCAGTTTTACATAG
AGTTTATGTCTACTGATCAGTGACCGAGACTGCGGCTTGATAACTTAGAATTTCTAAGGCTAGGTGG
TGAGTACGCTGAGATGTTGGAAACGCCAAATGTGGAAGAAGAGGTTTTAGCTGTGTTAAGCTCTAT
GTGCCGGGACAAGGCCTTAGGCCCCGATGGTTTCCCTATGATGTTTTATCAGAAATCTTCACTTCTAG
TTAAAAAGAATGTGATGGATTATATTGCGGAATTCTTTGAGTGTGGCCATCTTTCTAGTGAGCTTACA
ACTTCTTTCATAGATCTTATTGCTAAGGTTGAAGGAGTATATCATCTTAAAGACTTCATAGCTCTTATT
CCCAAGGTTGAAGGAGCATATCATCTTAAAGACTTTAGGTCGATTAGTCTTATTGGTGGGCCCTATAA
GTGGTTGGCCAAGATTCTTGCTTGTAGGTTCATGGGGGCTTTATCGAAGGTCATATCGGTAAATAAG
GGGGTTTTTGTGGCAGGGAAGCAAATTTTGGAAAGTGCCCTTATTGCACATAAATACATTGATTCCA
GATACAAAGAGAGGAAGAGTATTGTTTGCAAGTTGGAAAGTGAAAAGGCGTATGATCACGTAGACT
GGGATTTTTTTTAAATTATATGCTTGATCGTTTGGGTTGTGGTCGTAAGTGGAGAGGATGGATTCAG
GCTTACGTCAAATCAGCTTCCTTCTCCATTTCGGTTAATGGGTCACCGAAAGTGTATTTTAAGGCATC
ACAGGGTCTTAGATAGGGCAATCCATTATCTCCCTATCTGTTTGTGGTGATAGGGGAGGCTCGTAGT
ATGATGCTTCACAATATGGAGGAGAACAGGTTATTAAGTGGTTTTAAGGCATCAAATGGCAGATGGC
AAGTTTCTCATCTTCGATATGCTGAAGCAACTTTGGTAGATAACCTTCAAAAAATTATCATCTGTTTTG
CATTTGTCTCCGGATTGAAGGTTAATGTGGCCAAGTCTAAAATGTTGGGAGAGTTCGTGTTAGAGAT
GATCTTCAATGTCTTGCTGGTTTATTCCGATGCAAGTCCGGGTCCTTTCCTACAAAGTATTTAGAGTTG
CCTTTTTACATAGGAAAGCTAGCTAAACACTTATGGGACAAGGTCTTCGAAAGAATTTTGAGGAAAT
TAGCTGCTTGGAAAGGTCGTTCTCTCTCCTTGGGAAGTCAGCTCACACTCATCAAGGAGGCATTCTCT
AATATGCCTGTGTACTTCATGTCACTTTTTAAATGCCTCAAGAAGGTATTGGATAGGCTTAAGAAGCT

TAGAAGAGATTTCTTTTGGAAAGGTGCCTCCGATAGCAGGAAATTCCATCTCCTTAAAGTGGTAAGA
GGTGTGCAAGCCAATTCGAGATGATGGGGCTGGTCTGAAAAGGTTGGGTGATATGAATCTTGCCCT
TCTTGGGAAGTGGATATGGAGGTTCGGTTTTAAAGAGGGAAGACCTTGGCAGGAACTTATAGCTAG
TAAATGTGGGGTGCAGGAAAGTAGGTGGTTTGTGAAAAGTTCTTCGCTTTATAGAGCTTCTTAATTA
TGGTAGGCGATTATGGCTATTGAGCATTTGGTTCAAAGGGGAATTTCTTTTACTTTGGGCAATGGGT
ATCTTATTTGATTTTGGGTGGACATTTAGTGTGGCAATCATGCTTTCCAGGATCTATTCGCTAGCTTAG
CCCGGCTTGCTCCTAACCGGTTAATATCTTCCGCAGCTTCTTTCCTACTGCGGAGGTGGTTTGGTGTC
CTCCTTGCTGTAGGAATCTATTGAATGGGGAGATTGTTGAGTTCACCAATCTATTGGATCATCTCAAG
TGTGTTCTTCCCTCCCCTTCAGAAGTGGATCGGATGGTTTGGCGAGGTCAAAAATCGGGAAAGTTCT
CGGTTCATTTGCTTTACTCTATAATCTCTAAACCGTCTACTCTTTCTTGTTCGTTGCATCAGTTTCAGCA
TTGCTTCTATGGAGTTCCTCGAGTCGTGGTGTTTGTTTAGCTTGTGGCACATAAGAGGGTGCTTGCTG
TTAATAATCTTCAAAGGAGATCTTTAGTGCTTCGCAATGTCTGCTTGATGTGTACAGAGGACGCAGA
ATGAATTGATCATCTTTTTCTTCATTGTCCTTTTGTATATAAAGTCTGGTTCCATTTTTTTAAGTTTGCTT
CGTACTGATTGGTGATGCCGAAGTCAGTGGGTGATCCTTTGTGGGCTTGGCATGGGAGAGGAATTG
GGAAAGTTAAAAAGGCTAAATGGAGATTGACCCTCATGGGTATCCTTTGGGTTATATGGGGGGCTA
GGAATGGCCATTGCTTTCGGAACGAAAGAATGGAGATGGATGAGGTTAGTCTTGTGTTGGGTTGGG
CTGGACATTTTGAGGCCAATTTGGCTGCAAATCTTTTTGTTTCTTCTTAGAGTTGCGTGGGTGTTGTAT
TCTTTTCCTGTATTTGCAAGCGTCTTAATAAATTTGTTGTTATCTCTTAAAAAAAAAATCAAATGGTTAA
CATTATCGTCTCAGTGTAATTTTTCGTTTCCGCTCCAACCACAGTTCAGTCAGCGATTTGGACAGTAGA
TTCCCATACAACCACAGTTCGGTCTTCAATTTGAAGGCTCTCCACCATTTTCTAATTTGTGCTACTAAC
ATAGGAGTCTAGCCCCAAGATATTTCATACCGACAAAGATAAAATTTAAAGATGTCCATTCTATAGAA
AATAGTGGCTCTCTGCAGAGACGGAGTAATAATAGAAGAGTAATGCATCATAATTATGAGCCATGAA
ACAATGATAATGAAGACATCTTGCAGATTTTTTCAGTCATAAAAACATTAAAAGAATGCAAGGGCCA
AGTAATTTATGAAACTATTGATCAGAGAATCTGTCAGGGACAGTGAGAAAATTCAAATTTAAAAATT
TTCCCACTAGATTTTCCCTCATGTAGCGTCTATAAGACCAGCCTTGTACCATATCATTTCCCTTTAATTG
GAAACCGACTCTAACACCAACAGAAATGGCACAATTCTTTAACAGGCCCAGATCATACTAACCGTGG
CAGGATTGGCGACAAAAATCCTAGAGATCAGGTGCTGGCATTCAGGCGATATGTGAACATAGTCCG
GGATAGAGTACTGCACACTCAAAATTCGCTGCAAGAAAGAAGAACATGATTGATTGATTGATTAAAT
AAATAAAACCTGGTATATATGCTGCAGTAATGATGAGCCAAGTACTAACCTGTATTGTCTTCCTAAAG
TTCTTGGGCTCCTCAGGGTCTTCAAAAGGGTATGCACCCACAAGCATGACATAGAGGGTTACTCCAC
ATGACCACACATCCGCAATCTGAATTGCAGTAGTAAAAACAGCTAATAAGCAGAGTAAATTTAAAAT
GATGACCATAAAAATAAGAGATGAAAATATTTTTCCTTCAAGGTTTACAAGTGCTCATCTATCAAAAT
CATACCAATAGGAAAATAGCTGGTGCGTTATAACAATGAAAATTCATGGATTTTGTGTAATGACAAG
AGTCAATACCCGTTACAATTTCGTAAATCATGGGAAGGAAAAGGAATTTAATATTTGCATCCACTTTT
TTATACAACTTTTTCTACATCAGCAATAAAATATTTACAATAGATAATAGTTGAACTAATGTACAACCA
AATGCAGATACAAAAGGTGCGTGTAACAAAAAGCAAGGGTAAGATGGTGGCTCTCGCTGAAAATCT
GCACACACGAGATTTAAATCGATGAATTGAGTGTTCAATGCAGTGGTTTTACCTAATGCCGTAGACT
GCCAGAGATCAAGGACACTATGGAGATGCCATGATGATAAGTAAATGCCCATACATTGACACCGAG
AAAACTGCCCCTTAATGATTTGTTTGTAATCCTTCCAAACCTCTATGATAAAAGTGCAAGACTGAATA
CTTGATAACATTCCTGTATTTGTGTTTTTTGCCATATGGGAATAACTTCTTGAATTTGAAAGACCTCTA
GCATGTATGATGCCTTCTCTGAGTGAATGTAATTTAATCTCTTTTGCCTTAACTCTCTCCAATGAAAAG
ATAAACAATTTCCATGCGTTTGGATCAGACAAGATACAAAGGATTTAAGCATGTAACAGTGACACTT
CAATTGGCACAAACTTCAGTCCTCCAGCAAAGAAAAAGATGCTTCTCCATCATATAGAAAAGAGAAA
ACAAGAGTATGACTTCAACACACACAGGAAGCACCCCGTCACTTTGCACATAATCTCCTACCGTTAAA
GACAATTGGCAGGAATCTAGGATGCATTTCAAAACAATTAATTCTGAGCTCACATTTTGAAATGCATG
TTAAAATTCCTGCACACCACTACTGGATGGCGGCAGATATGTCAACATCGACTCTCCATGCACATGAA
ATCTTTTCCAGAAGAAGAATGCCTTATGCATGATGCTTCTAATACATGTAAATATATGGGAAATGCTT
ATGTCCCCACCTACATGAGGCATTAGCAACATCCCAAAGCTTTTAATTGATATATGTGACAGCCAACC

ACCAATCAGGACCGTCTGAATGTTGTTCATTTTTTTACAACCACCCGTCCTTTACAAACAATAGATTAG
ATGGTTACCTGATTGGTTGTCCAACATGTCATCTAATAGCTTAAGGGATGTTGTGGGAACATTAGCAT
TCCCTAATATATAATCCTCTAGTTGCTTGACTAGTTTCCAATTCCCAACTCCACACCAATCTTAACACTG
ACTCATGACACACAAGTTTCCTGGACCTAACAACCTTCTAAAACCAAAACTCGTTAAATTTAAGAACA
AACTCCTAGTGGGGATGTTGTTGGAGATTTTTGCACTATGGTCTGCCCCTGGTGGAATGAATAAATG
GACAGTCTGTATTGATAATTAACCATCTGACGTATCATTCAATAGATTCGGGGATGTTGTGGGGACA
TTAGTATTCCCCTATATATGTATTCCTCAAGTTTCTTGACTAGTTTCTAACTCCTAATTTTCCGGCTCTA
CCCCACTACATCCAGTGGATCAAAGTTCACACACGAGTTTCCTGGACTAAATGACCTTTTAAGACCAA
AACTTTTAAACTCTAAAAACCCCTAACCAAAAGTTTTGAAGCCTATAAAACTCTTACCAAAACAGAAT
TTAATCCTACTGAAGTTGGACTTCAAATCTTGGTTGGAAAAGGCTTGATGGGAGACCAGAGCAGATT
TGGGAGGTGGAGAAGTAAGAGTCTGCTTCAAAAGACTAAAATATATAAAAGCTAGCAAGTGGGAGC
AGAAGCGCAAGTCAGAAGCACGAGTTTGATGTAGAAGCAGCAACTTGATAGAAGCATCTCAAACAA
ACTCCTGTGTCTTCCCTAAAAGGGCACTAAGCTGCCTGTTCAACTTCCATTCTGAATAAGATCTCCCAC
AATCAATGACAGCATGACATCATGCTAACATAAGCTGTACGGCCATCAAATGTGTTTTGGTTTTTTTTT
TTTTTTATCATTGTTCTCTTCCTCTTCCTTTTATGGTGCACTTCTGCCTGTTTTGGGGCCACTTCTTTTGC
TGGTGGGCACTGTTTCCCTGCAGCTGATTCGGAACTGCATCAATATTCCAACCCAATTATAATAATAA
ACCCGATAAAATATTCCCAATAGTCATGTGGACATTTTGCCACTTCCCATAAGGTTGCAGATACACGG
TGTAAGATGGAATCACAGGTCCCTAACTTCCCATAAATTTGTTTTGGTTTTTTTTTATTTATTATTCTCT
TCCGTTTCCTTTTATGGTGCACTTCTGCCTGTTTTGGGGCCACTTCTTTCAGCTGGTGGGCACTGTTTC
CCTGCGGCTGATTTGAAACTGCATCAAAATTCCAACCCAATTATAATAATAAACCCAATAAAATATTC
CCATTAGTCATGTGGACATTTTGCCACTTCCCATTAGGTTGCAGATACACAGTGTAAGATGCAATCAC
AGGTCCCTAACTTTCACTAGCCAATGGTCTTCAAGAGGCACCATCTTCTTTCCAAACAGATCACAAAA
AATACAAAAGGAAAAAAGAAAGAGAGCATGACAAAATAAAAGTGCCAATTGTCTGTATTGGAAGCA
TTATTGTACTTTAATCTATGAAAGGAACCCAGTTCTACAAAATCCGAGCTTTACGAAAGACATTAATT
GAAGAAATGTGGCTGCTCTTGATATTTTCTGACTCCATCAGACCACAATTGATAATCACTTAAATGGC
TATAAAAACACTCACTAAAATCTACTCCTAAGCTCTTACATTTGGACAATAGTGCAGAATGAACACTG
ATACAGCTTCATAACCCACAATACATGAATCTTCTCCATAACCAATATGCTGAGGCCAAACAATTGGA
GTTTTATTCCACGGTGGCCTCAACCTAGATCAATGTTGGCAATTTTCTCCTCTAAAATATCAACAAAAC
TTTGGAGTGGAGTTTTATGTTCGACTCCACAAGAACATTCTTCACAATCAACTTAGATATAACTCAAA
CAAACTGTTCTCCAATGATAATCTAATGTAGGGGTATGTGATGACCTAACTCTAAATGTACATGTGAT
CAATAAGTAGACATTCAAGCAATGAATTCATTTAACTATCCACTATCAACTAAGAGATTATGAGAGTT
CAACAATAAGATGATGATAACCCGTCAGGATCATACTCCATAGCATAAACGCACATAAATGAGTAAA
AGGGGGACCTAGGGATATGAGGACATCCCTAAACCTAACATATGCCAACCCACGATCAAATCTGAAT
CAAGTTAAAAGATGGGTCTTACCTTCCCACACACCTTGAAGATTTAAGAAAGAGAAAAGGTGGTACT
CCAGGATGGAATCCTCAACACCCAAAGGCCAATCCTGAAACCCTAATCTACGCGAAGAAGAACTTCA
AGAGAAAAAATAATAATAATAATAAAATTTATTAAAAATATCAGTTAGGGCTCCCCCCCCCCCCCCC
CCCCCCCCCCCCAATTTCTCCGCAAAGGAAGAAAAGTCTTACATTTCCACGCACCTTGAAGATTTAAG
AAAGAGACAACGTAGTACTCCTGGATGGAATTCTCAACACCTAAGGGCAAATCCTGAAACCCTAATC
TACGCGAAGAAGAACTTCGAGAGAATTATCTTTTTTTATAAAAATATCAGTTAGGGTAAAAAAAAAA
AGCGTGGTACTCCTGGGTGGAATTCTCAACACCCAAGGGCCAATCCTGAAACCCTAATTTACACGAA
GAAGAACTTCAAGAGAATTATCTTTTTTTATAAAAATATCAGTTAGGGCTCCCCCCTGCCCCTCTTGAA
TTTATAAAAGAAACTTAAGGAGGAAAATTACATATGCGTCCCTAATGTCCAAACTTCAAAATAAACTA
AACCCTAATCACAAAATAACATCTGAACCGTCCATAGAATCCAAAACTATAACTAAAATAAATAAAAC
TAAGAAAACTGGGAACGGGCCACCAAAGTGGGCCCCGGGTCGATCCAACGGTTGGATGGCCCGATC
GGAAGAATCCAACAGTCCTGATTGCCATCCAATCTTGGATCCATGGTCGATAAGTTTCAAATCATCAA
TCCACACACATTTCCAATGAAATGAGCCTCCATATGCGTATGTACGTGTGCGGGGTCTTTGTAGTCCA
TCAATGAAACGGCCCGATGGGCGCAGTGTCCATCCACATCAGCTCATCTGTTGTAACGCCCCAAATTT
TCAAACTTTATTGGAACTTTTCAGAATTTTATTTTGATTTTATTGTAATTAAACATCAAGCATTCACTTT

AATTTTAAAACTACGGATCATCCGTGAGAGTTTTGATAGGCGAGCCTTTACAAATATCTCTTAGAATG
ATGCCATGAACGACGTAGAACGGTTGGATTTCGCAAAACTCGCATTTTTTCGATATTTACGAAAATGC
CATTAAGTTAAACTTAAGTTAGACCTGCATTTTAAGTACGCTCGTTTCCCACGATCAGTTTGTAGCAA
AACTTTAAATATAAGCTATCGAATTTTAACCTGTACACGTCAAATTTCGGCCCTCCAATCATTATGGAA
GTGCCCCAATCAGAAATCGGGTTGTAGGTGGCCCACTTGAACTCGGATTTGCCTCATTTTTGGAGCG
ACACCCTAAAATTACCTCGCCTACCTAATGAACAGGCTGGATTTCTCGCAATCATCATAGTGGGACCC
ATTGTGCAGTCGCACGCATGTTTAGTGCATGGTCAAAGCACGTTTGATCGTGCAATTTTCTACCAAAT
TTCCGTTTTTTCCTCCAACGCATGATCGCTGGCCTCACATTTGGGCACCGTGTGGACTCCATCTTAAAA
ATCTAAACCGTCCATTCTCTTCCTAGCCATCTTAAACTCCCTCCCATGAAGTATTTTCACCAACCATTCA
CCACATGATCCGATCTTATGGCCCGATCAAACCATAGTTTTCTTGGACGTTTCATCTGGGTTGTCACG
AGAAGCTAGATTCCATATCTAGGCTCTAGTGGACCCCACCTAGCTAACCAACGTGACTTTTCACATCA
AAAACCGAGATTTCTCTCTTGAAATCCACCAACCAAATCTTTCTCTCGCGTAAACCACCGACTAAACCT
CCGTAGAGCTTGGATTTTTGGACCCCACAAACTTCCTTGGACCCATCTAGACCATCCAGAGGTGTCCG
ATCATCAGATAAACCCCCCGTGTGCAATTCACCATTTTCGCACCACTTTCTTCCTCGCTTTTCACCAAG
ACCGTTTATCCAATCATGGGCCCCACTCCCTGATCTATCACGACCATTTTGTAGGCCATCCTCACTATA
CCTAACGTCCGAAATATCTCAGCCATCCATCCTGTGTGCACTTCCCAAAACCGGCCCGAAACTCACTT
GAAAAAACTCTCACGTGCGTCTGCAGCCTCTCCCACTGACTAAAGGACGACCCCCCCCCCCCCCCCG
GTGGAACCCTCACCATTCACGTTTTGAGAAAGAGAGAGAGAGAGAGAGAGCACTATTAATGCTGCC
CATCTCATCTCAGCAAGAGAAGAAGAAAAGAAAGAAGAAGGAAGAGGAGAGGGAAGAGTCACGCC
TTGGCGGATATTCCGATTTTCCAACAATCCCTATGCCCTCTAAACACATCAACTACTAGAGGTGAGGA
CTTCTTTTAAGTTTAGGATGATAGTTACATGTCTTGGTTGCGTATGTTGTGTCATGTATGCTATCATTC
TTGAATATTATTTTTTTATTCTCGCATGATTATATAAATGCTTTAAAACCCCATATTCCGATCACTTGTT
GATGCGGACGTGTATTGGACTTGAAGAACGGACAAATTATTGGATTGTTGTTACAAATTTACACATA
TGTGGTTGTGTAATCATCTTTGAAGAAACCACTATCCCTTCCGCATAATGTTGAAAGTTGATTAGTAA
AGCTTAATGAAGATCATTTAATTATGTTATTGTAGTAGCTTAGATGAACCCTGATCTGTGGTGTTGAA
TATCAGGACTGCCCAGGTTAAGAGGTTGCCCTAATCTGAGACGGTGCTTTTAGACTGTCTGATTAAG
TGGTCGCCCTGACCTGTGGCAGTGAAATAAGACAACCCGATTAAGTGGTTGATAAATCTTTTTTTATT
ATTATTATTATTCCGTTAATTGTTTATAAATCTTCGTGAAGCTATAAGTGTACATGTGTTGTCATGAAC
CTTAGTGGTACAGTCCGTCATCCTTGGTGGTGATGCGTTGGGTACGGCTACTTTGATAAATGATGAA
CCATAGAGATGGTTATGGTGGTACATAAGGTTGGTCACACGTAATTGGTTGTGTGCAAATTAGTAAT
AATAAAGGATTGGTTAATCTCATGTATTCACTTGGCTCCATGTGAATGTACGGAATTAGGAATGGGC
TAAAGTGTCATACTTCTCCCATACGCTGTCTCGTGAGATTTTGGCTATAAATGTCCCACTTCTCACTTA
GTCCGGCAAAACTATGGGCTTCGGTGTCCCAAAAACCCTGGCGGCGTGTCTAAGGAATCCTACGCCA
GATCATGTATTCTATGCATTGCATTAAGTAAGAACTATGATTGTTTTGTTATGTTTTCATATGATTATT
GTGCTAACCTGGAAGTAGTTCTTCACTATACCGCTTTTGAAGGTTTTTGTTGAAAAACAATATAGAAT
ATGCAGGTGACGATCCACAGGAACCCGAGCCATAAGGAGATGACTACGAGAGCTTGTGAGAGCAG
ATGGTAGAAGACAAGGCCGGTGTGGCCTAATATTGATTTAAGTTTTCAATAGTTAAAGATTTGATTTT
GTAATAAGTCTCAGTAGATGGACATAGATGAGAACAATACGATATTCGACTTCTTTAAACTAAAGCG
ATATTATTTGATAACTTAGTTTAGATAATGAAATGATATGCGTGTTTCTGTCATTCTGCTCGTAAATGA
CATTTATGGTGAAATATGAATGATTGTGAGTGATTAGGTAAGGGGTATACCCTTTACACATTCTAACC
TTTGGGAATTTAGCCATTGTGTTATACACGGGAGCCGATATCTCAGGGTGCTACATCTGCTCCAACAC
TCGATCGGTGACGTCTCCTCCTCTGGTACACTCTAAAGGGTGCACCACAGATGTCCTCATCATAATCA
CAACCTCATTTCTCAAAAAAGTGACAATTACACCCAGTTCAAACATGCCCCTAAGAAATATCCATGTG
ATTCAAACACCTCCTTAAGGACATGTTTTGCAAGTTGCAACAACCAAGAATGCGTGTGTAGGTCTGAT
GACAGTCATGTTTAAGTGGCAATAAAAGACGAATGACAGCTGGTTGCGTTTCAAGATGAAAAGAAA
TTTCTAGATGAATGCCTACAGGCATATATCGAGCTCTCTCTTGGTATAGAAATTGCAGTACTCTGCAA
CTCAGATTTGGTGAGCATCGCATCATAGAGGCATGTTTAAGTAGTTTATCTTTCAAATAGGTGCTACT
AGAAGACTAGTAATGCTCCAGCAACTATATTTGAATCCAAAAATGTGCCACATCAACAACATTCAGAT

ACAAGTAGCTCAGTTTATAGGTCGCATCCTTGACTGTGGCATAGGTACAATGAATTCATCACAAGTCA
TGACAAAAAGATAGCTTTTAGTCATGCAGCTTGATCTTCAGGAAAATATGCATGCTTATGCATGCCCA
TATGGATGGAAATGTCTGCCTGTGTGCACACACAGGTACAAAAACATGCACAAAATTGGAACTTTGT
TAGCCCCACACACATTTATGCAGATATACATATTGATCTAGCACGTGCCTGGGACATCTGAGCAGTGA
ACAAGGTGAACCTTGCCCTGCAGATGATCTAGCACTAAAATCAGGCCAGTCCAGCACTGGCTGAAGC
AGTGGTTGACTGCAATTTGAGTCCGGCAATTATAGAAGGAGACTCCAAGAATGCCATTTTGTGGGTC
ACAGGTGGTAGTTACCCCTGGCAATTTGCAATATGATTGAGGAGCTCTTGACTTGAGAACCGAGCAT
TTGGCATTCACTCATGTGTCTAGGAGTGAGAATGCTGAGGTGGAGATTCTTGCTAAAAACATGGTGC
TGGCGCTAGGAGATAGCTGCTGAAGATACAAAGGCTGCTGTTCGTTGAGAGTAAGGCTGTGTGTTG
GGTTGTTCTTGTTAGTGTGTCACTGGTGATTGTGATTAGTTTCTTTTATCTTTTTTCTTCTTCTTCATTTC
TTCTTTTTCTCTTTTTTCTCTTTTTTCTGGTGCCCCTGGAAAAGCCCTGTTGTGTTGCATTTTTTTTATTT
AATAAAGTGGGTTATTCATAAATAAATAAAGAAAGAAAGAAAGAAAATCAGGCTAGTCCAGTCATC
AGACAGAGCACCCTATAGAGAAATCAAAATATCGCCGATGCCACTGGTGATACAATGATCATAGCAT
TTGTGTGCATATATGACACATGTAAATCTGCATGTGGGACAAGCAGACATGCACACAAGAAACAATC
ATATGCATGTCTGTGGATAGAGAAATTTCCTTCAAAAATTCTGTTGAAACTTAAGCTTGGTGCACTCA
GCTCTCCAAAGTAGGTAGCAACTCATTAAAGTTAACTGAAATTAAGTAAAGAAAAAACGAAGAAGA
AGAAGAAAAAGAAAAACGGTTATTATAATTTTATTCTTTCAAACTTTGATTAGGTAACTCCCTTGACA
AGACTTTTTTGTGATCAGGATATCTAGTAACTTAGGCTGAAATCAGGTTTCAGATGATGTGCTAAGGT
TACAATGACCAAAAGAAAAAAAAATCAGTCTATTTTCTTGTACCAAGTAAAATATATGGTAACACTAG
CTAGACAATTCAAAAGCCACATTAAAATCTTCTGAATTCACATATTAGTCCTGTAGGTAGAATACTGG
TACTGACAACATGCAAATATAACCATGTGTCAATAATAAATTATCTTACCTTTCCATCATATTCCTTCTT
GAGTAACACTTCAGGTGCAATGTATGCAGGAGTCCCAACAGTTGACTTTGGTTGCGAGTGCAGTACA
GAAGACTGGGGAAAGCAACAGTAAGTTACAAGTGCACAGAAATGAATGTCATAGCTATAATCTAGG
CATTCTAAATAAAAAGACATAAATGTTGCTAATCAAAGAAGAATGGACTATTTCTTAAAAATTGATCC
TTTCCATAAACGGAAAAGAAAATCGGTTTTAAGTCCCACTGAAACCAAATGGGGCTTAAAATCCAAA
TGTCCAAATGTTTGTGCATCTATCTGTTTTTGCTTGCATGTTGGTCGGTTCACTAGACTAAGAGCCAAT
AGTAATTCAAATGCATATAGGTGAATGGCTGGATGCTTTACAAGCATATGCGAACATACATATACTA
ACTATGCAGGAAGAATGATAACATCAAACGCAACTACCTTGGAATACCCAAAATCACATATCTTCAG
ACGAGGAGCAGGACTACCATCCAACAGAGTGTTTTCCAGCTTCAGATCGCGATGACAAACTTGCTTC
ATGATAGAATAGAGAACGTAGAGGTCAGTTTAACAACCCGGGTGGCATATCAAATATAGACAACAA
AAGCCATCTCAAAACAGACATTCATCTTACATACCATCGAATGACAGTAGCAGACTCCTGATATAAGT
TGCTGGAAGAAGAAACGTGCCTGCAGAGGAAAGCAGGATGCTATAAAAGAGCACAATGTGAAGAG
GCAACTTTTTCTTTGAACAATATTAAGGATCAACTTAATCAATGGATTGGATTAACAACCTCATCCTCG
CTGAAGCGTCCAGCATTACATATACGCTCAAAAAGCTCTCCACCAGATGCATATTCCATCACAATGGC
CAGATGGGTCGGTGTTAATATAACCTAGAGAAGGATTTCAAAAACAGACAGATGTTAAATTAATCCA
TCATTAAATTCAATAAATAGTAATAAGCAAACCCTTTAAAACTAACAAACCTCTTTGAACCTAACAAT
GTTCGGGTGCCGCAGAGACCTGTGGTTGATAATTTCTCTTTGTACATTTTCATCTATCTGTTACACAAT
AACAGAATGAGAAGAAGTAAACCAAAAAGGTACTCTACATCTAGTCCAATAAAAACCTAGTTCACAC
CCAGGTCCATAGCTCAAGTGGCAGACTAAGTGAAGATAGCCTCATTTCAACACTGAGGTCATGGTAT
TGATTCCCATAGTGGGGGTGGCTAACATTGTGATGTGACTAACAGGGGTGGGTGGGTGTACTAACA
ATGGAGTGTGTGAACTAACCCATAAAAAATAAAAAAAATAAAAAATAAAAACCTAGTTCAATCCCTA
TAATTGATATACCAAGCGCACTCACTAGTTCCAAACAAATCCTCCATTACTGGTTTGAATCCATGTTAC
CTCATGTGCATGGGAATCAAGTATGGTGGAATGTCAAAACCCAAATTCTTAGATAAAAGTAAGCAAG
GGTTGGTTATAGCTCATAGGTATACTTTTGAGCTACATTATTTAACCACAATCAAAAGTTGAACATGT
TAGCCCTACCGCACCCTAAATTCTAAAACTAACCAGTTAGCTCCAGATTTGAAATCATAATTAGATGT
GGAGTTCAAGTTATAGTTGCATGTAGTGTGAAGCGAAGCAGAAACAGACTCAGAGCGGAGCATGTA
GTGTGAAGCGAAGCAGAAACAGACTCAGAGTGGAGAAGTATCGATTTCAAAATGTTAGCAATAAAT
GACTAGGAAGCAGGAGATGAGCGGGAGTTCGGAGATAACCTGGTGAGAGAGCAAACACCTAGGTA

GAAGGTTCATGTAACTAACTTCAGGTCCTGGGAAGTAGGGTCCATCATCCTCATATTATGAGTTCCTG
AGTCAGGTACTTAAGCTTTTTGAGACCCAAGGGTCACATGGACAGCATCACACCTAAGTATCAAATC
ATCACGTGCACTCCATGGGTACACATGGCACATGTGTACAAATTCTGGAATGCTCATCTGATGAGTC
ACACTGGCTGATCATCAGGGTATAGAGAGGGGAGGAAGCTAGTTGACCAATGATTTAGATTCAATG
GACAAAAGTCCCCAACGTAAGCAATTACAATCATCTGAGAGATGTGATCTTTACGATTTGTCCCATCC
ATAGTGGGCCCAGGTGACGAGCTGCCAAACATTGTAAAAGCATGCAATGAGTCCAACAGGTAAAAA
TTAGTTCCAGTGTACCATATTGATCACCACTGAAACCGAGACACATCGGCCAAAGCAGCCGATACAG
GCCAACACATAATGGGACTTCAGTTCCAACAAGTGGGGCCCATGGGCAATGGGACTTCAGTTCCAAC
AAGTGGGGCCCATGGCTGGGTGTTAAATCGTAAAGGCTACATTGTGGAAGCTCTTAACTTGTTTATT
TCATTAAGAATAACATGAATGTATAGAAGACAAGAGAAATTTGTTAGAGATCTATGGAGATCTTAAC
AATAATAGAAATATCCTAACATAGAAGATTAACCCTAATCTATTCCAACATCCCCCTTCAGACTCAAG
GTGCTCGGAAATGAGAAACTTGAGTTTAAAAGGTATGGTGAACATTTATTCAAGCATAAGAAAAACA
AAATTCAATGATAAAATGAGTTGACAAAACGACAAGTGGCGATGTAAAAACAAGGGTGGATGCACA
TTTCCAGTTTGTGGGAGTTCAATCATTGCTCTTCCCGTGGTGCTAGATGCTCCGATAGCAAAAACAAG
TCCAGTCCAAGTGCCCCACACAAAAAGGGGTCCAGCACAATCCATACGTGCGGCATAAAAACACGTC
TTGTCAAAAGGCCTGATAAGGAGAAGATAAATCGTCAAAGGTGGTATATGCAGGAGATGAATCGTT
GAAGGTGGGGAGCCACAAGAAAGATAGATCGAGGCCCACGAGGTTGTGGAATGCAGGTGATGAAG
GAATTGTACAGTGGATCGGAAATGGTAGGGATGGCAGCACGGCAACATGATGCATTGAAAAAGCA
GCAATGAAAGATCTCAACTGTCCATCAACCTCACCTCTCATCCGATGGCACCAAGATCTACAAATCCC
AGTCTGACAGGCTCAAATGAATCCTCCTCTGAAAGCCCAACCGCACAAGATCCCTCCACCATCGAAG
GCTATGGGATTTGAAGATGGGAGGACAGACAGATTCGAGGTTGACGTAAAGATTGAGGATGAGAA
TGGGTTCCGGATATGGGAAGGGTTTTGCAGATGGGCGGATTGTAGATGGAAACAGCAATGGTAGTT
CGATTTTGCGATCGGTTCGGATCAAACAACGATGCAGTGGTGCCTTGCCAGAGCAGGACATCTAGCT
AAACCAAATCGCTGGATCTAATGGTGGGTTCTGGCTTCAGACAGATGAAGACAACCTGAAATGGAG
ATGCTGCAGGAGCCAGAGAGAGGAGACTGGCTGCAGGAGGAGTTAATAGGTAACGACAGGTCTGG
GCAGCCACCAATAGATTCCAATGTCAAACTTGAGATGAAGAAGAAGGGTGATACTTGAGATGGATG
GCCGGATGCAATGGTGAGGGATTCCGGCAATGATGGGAGTGGGCAACCAGCAATGAGTTCGAACG
CTGGACCTAAGATAAAGAAGAAGGATGTGGTAGTGGGTGGCAGCGAGACGGGCAATGCAAATGAA
GGAATGGATCAAGATTAACATCAGATGGAGGGTTGACAATGGCTGGTTGTGTTGCAATTACAGCTTC
CAGCAGTGAGATCCAAAAAGGTCTATGGTTACAGCGTTCGGCAGACAAATATGACAAGGTTGCGAT
GATGTTGCAGGATCTAGTCGGAAAACTTGGGTTCATAAGGCAACGGGGATAGATGTTGATGGGTTT
CCGGTTCTAGGGCTACCAGAGAAGAAGATGTAGGGGATGATGAGTCCAGATAAAGGGCACAAACA
AGGTTGCTACAAACGGGCTGCCGGAATGTTCTATTACAGCAACATGCGCCAATCTCCAATCGATGAT
CTGAAGAGGTTGCTTTGACGTGTGATCTCGTGATGCACAATCTCGAGATTGGATGGTGGTCATCACT
GCCACTATTTGGGGACGGTGGCGGCAGTGAAGCTACCAACAATGAGAAGGAATGGAGAGTTAGAT
GGGAGCTAGGATTTTACCCTATGATACCATGTTAAACCATGAGGGCTACCTTATGAAAACTCTAACTT
GTTTATTTCACTGAGAATAACATGAATATATAGAAGATGAGATAAACCTGTGAGAGATTTATATAGA
TCTTAACAGATATCCATCTTAACAATAAATAGAAAAATACTAAAATAGAAAATTAACCCTAACCTATT
CTAACACTGGGTGATCCAGAACATTGATCAGATGTGTTCCACCACGGATTAACTATTCCCCCAAAAAA
GATACATCTATACACCATCCTAACCTTTTGATTTATGGCCAACAGATTGATAGTTCAGATAAGAAAGA
CAACAAGCTATCGATATTTAACTGAGTTGTCGGAATCCACCGTATCATCCGACTTGGGACGATTCTAT
GATAAAATCAATTCAAAGTACATAAAACGATCATGATTGTTTTCCAACTTACAACCCGAATATGTTCG
AACAGACATTTAAAAAACAGCATTTCCAAACTCCCAAGATCACCAACAGTTGAACTTCAAGAAATGG
GGCTCTTTTTGGTTGTATTGGGAAAAAGAGCTCTTTCATGTCCACATGAATATGGTACTGTGAATTTT
GGAAAGTATATACTCGAGAGTAGAAAACCCAAAAATCCCAGTCTTGTCTCGTGGTTTCCTGCTTATTT
AAAGAAAAAATCACCCACAATTCATCAGTTTATCCACTGACACTCCACCCATTTCCATAATCCAATATC
AAATACCTCAACTTTAAAAAAACAAAAAAAACAAAAAAAAAAACAAAAAAACAAGAAGAAGAAGAA
GAAGAAGGAAGATGACACTTGCAGCCCCAGCTTTTTTTTCATACACACCCCTCTCCATTGTAAAGGGT

GAGTAATAAAGTCAAAAGTAGGTGCCGCAAATGAAAAGAAAAGGTGGTGTGTGTATAAAAATTAAG
GGGTGTGAATATCAGAACAACTGAAATTTCCCAAAACAAAACCCAAACCCATCTACAGAATCCAGCC
ATCCAAACAGTCCACACCTCTTTCCTATGAAAACACTCAAATTACCAGAAAACAACCAAGTTCCTCGA
AATTACCTTCTCGCCCCTCTCGATGTACTTGACGGCGACGAGCTCTCGGGTCTGCTTGTCCCTCATCA
GCCTTGCGACGCCGAAATTACCCGACCCGATGTCTCGGACGAGCTCGTACCGATCGCTGTCGTGCAT
TATCGGCATGTCCATGGCGGGGCCCACCGTCAGCGCCGCTCTGTCCAT

>Lchi01348

ATGGAGGAGAGATATGAGCCGTTGAAAGAACTGGGGTCGGGGAACTTTGGGGTGGCGAGGCTGGT
GAGGGATAAGAAGACAAAGGAGCTGGTTGCTGTCAAATACATTGAGAGGGGGAAGAAGGTAATAT
TTCATCAAACCACATTGATCGATGGATAGTTCATGTTCTTTCATTTCTTTCTTTTTTTTGGCGATTGAGA
TTGGTATTGTTGTCGGTTTTCTTTTAACTCCTATGAATTTCGATTTTGGATGAGATCTAAACTTCAATTT
TGCAATTCAAATGGTGAATGGACGATGGTCTAAAAATCACAACGTTTGGACGACTCTAGCCCTTTGC
ATGGTGGAATACAAATTATCTCTATTTGCGGTAGAAAAAAAATCCGCAACGGCTCTCATTAAATGAA
GAACGGCCCATTTATTAGATGGTTAAGATAGTCCAATCAAAGTAAATTTTGGTTAGGGGCCAATCAA
TGGCTGAGCCGGCAAGATGCCTAGTTCTGATCATCATAGACATGTGCCAAGTATTTGGCACACAATC
AGTCAGCTGCTTTTTTTTCTTTTTTTTTACTTGTGAAGTTTGGCTTTTTTAACGTGTTTTATCCCCATGAG
AGGGTTTGGATTTGGTTTTTATTAGGAATGGAATATGGATTTCAGTTGTTTCCTTTGTTTTTAAATTAT
GCTTTTGTTTGAATTAGTCTGTCCTTATTGTTGCTGTATGGTCTTATCTATGCTATCAATGGTGGGATT
CTCCATTCTAATTACAATTGATTTTGTGATGTTTATGAATTTGGTTCTCTCTCACTGGTTTGAAATTTGT
GATTGCTTATTATTTTTTTAATTTCCTGTTTGGATTGGGAGTAAACTAGAGGAAGGTATGGGAAAGA
GAAATTGTGACATAAGAAGATGGTAAATTGACATCAATACCAGGGGAAGGAAACTGTTTGCATCTGT
TTGTTTTTCTAAAGAAATTGTGAAGGAAAGTGCTTTCCCTCTGCATTTTCCACTGATGGTCTGCAATTT
TTAAAGGGAAACCTCTCCCCTCCAATTTTGTCATTTAAAACATCACATTAAAGGGAAAACACTTTTACT
ACCATTTGACTTTTTCAAAAGAAAAAAAGAAAGAAGTTTTACTCTCTTTCTTAGGAAAGCAAATGGAT
TTCCTTTCTTTTTATGTAATAAAAAAAAGTCAAATCAAAGTAAAATTATTTGCTTTCCCTAGTTTACCCT
CAATCCAAACAGGTCATTAGGCTTTTTATTTTTTTTATTTTTTTATTTTTTTAAATTGTCTGAAATTTGGA
TATTGGGTTCAGATTGATGAGAACGTGCAGAGGGAAATCATCAATCATAGATCATTAAGGCATCCAA
ACATTGTCCGGTTTAAAGAGGTATAGTACTCCTTTCATTACATTCGGTACTTTCAGTTTTCATTTCATTG
TAATTGTCTGTTGATGATTAGCATGATAACGATTGATATCATGGCTAATCTATAAAAATCTTTGGCTTC
CTTTTTGTGTGTTCTCTTCCCCAAGAGAACCTTTGTATGGAAGGGGAAAAATAAAAAAACAAAAAGT
GTATTGCTTCTGATTGCTTCAAGTATTGTTTCAGTTGAAATTAGACTACGTTTCTTTATTTGATTAACAT
GAGAGGGAAGGTGTGGCGATATTTCACTAGTTCTCGATGGGAGAATCTGATTTCCACTAACCACCCA
ATCTTTTTTCATTTAGGAGCAGTATTCAATAACCAATGGTTAAAAGGTGGATCTAATGCCAGGCAAAC
ATTTGGCTTTCAAGATTTTTTATTTTATTTTATTTTATTTTATTTCTCCGTGTGTGTGTGTGTGTGTGTGT
GTGTGTGTGTGATTAGAATGCAAGACTATTGGAACTGATCAGTTGTGGAGATTTTTGTTTGACGAAA
TTAATTAGTCTTGAGGGTTGAATTTCAATCGAGGAAGGTCTTATATTGTTCATAATTTACTTTTTTGGT
CTCAAGTTATGAAAGAATGTTAAGTTTTTTTTTTTTTTTTTTTTAAACATCACTCAGAAATTTCCAGTTT
TATTGTATCTTTATTTGATGGTCCAAAGGTGTTGAGAGTTGACATTCTTTGCCAGATCTGTTCAGTTGT
GATTTTGAGGCCACTAACCCACCAAATAAAGCAACAAATCATGTAGGTGGAATTACCTAATCCAACT
ACAGTAATGTTGGTGACATCCAATCTCCATGATTTGAATGTAAATTGGTGCTATGAATCTCATATACA
AGCATAACAAACATCCATTGCAGCATTAACAGATTGTGTTACTTCTATTCACATGCCTGTTGGGTTAT
GTTCTCCACGTAGAGACAAAGTGGGCCCCATGAAGATTACAAAAATCATTGATTAGATTATATCATTC
GTATATAATAGTCTTTTGATGTAGAATATTTCATATTTTGGCACATTTTGGCTGGAGATGTTGTGTCTA
CATGCATAAAACACATGTTGGGCATTGCAAATAATACACAAGAGTCCCAGTTTTCTCATATCCACTGT
TACCTTGACATGGTATCAAAGCTGTTGATTTGAGGGCCATTAATCATTGAATTTCATTATCCCCAACTC
CTTCTGGCTACTAGCATGACCATCCAGCTCACCTTGTTTACCTCTACACGCCCACGTATGACAATTGTG
TGCATGGACATTCATGTACTTGAAGACAGAACCCTGGACACAGTATGATTACACCCACAAACAGTCA

TCCCCAAGAATTGTGAAGAGAGCAAAGTGATTAATATGGGAAGATCGTTGGATCTTTCGGGTTCGCA
TGGAAAATTTCTTGTGTGGATCCTAAAACATGGAGAACAACAATATATACTGTGGTTATTATTATTCT
TCAATTTAGTGCTTTTCATGCAGGTTGTTTTAACGCCCACACATCTCGCGATCATTATGGAATATGCTG
CTGGAGGTGAGCTCTTTGAGAAGATTTCCAGTGCTGGACGATTTAGTGAGGATGAGGTCTTAGAACT
GTAACTTGTTTATTTTTAATTATTGCTTTCATTTCATTGCACATCATCATTCGTTGGGCATGCTTCTTTT
GCATATATCAAAACTCTATCTATCTCTAATAAAGTCATTAGCTGTCTGTCCGGTGATGTCTGCTTTTGC
AACAGGCAAGATTTTTCTTTCAGCAGCTAATATCTGGAGTCAGCTATTGCCATTCTATGGTAAGTCCT
GCATTGTTTTGGATTGGATGTCTATGCGATTTAATTAATTTTAAGAAAGAATGAGGAACAACCACGTC
ACAATTTCCTTTCTTTGGATTCATACCTAGGTTCCAAATTGCAATTATGTTACTTTCAAGTTAGACTCG
AGGGCTTGTTTGGAGAATGTGAAAAGAAAAGTGCGCCTATTATCCAATGATGGATTCCCATGGTTTC
TGTGAACACTATTGAAACACAGATTCCATTTTCTGGGGTCTTGTTTGTATAGCAAGTAGAAATCTCAT
TTTCTTCTCACAAAAAAACACTTCGTATCTTGTGCATGAGAAACCGTCTATGGTGTACTATGATGTTTG
GATAATGATTGCTTTGTGAAATCCATTATTTCTTTTCCTGTTCAAACAGTGAAATGTCCCAAACAAGCA
AAAGTGCTTATAATTATCATTTTATTTCATTTCTCTTCCCACTATCCAAACGTGAAATTTGAGGGCTCG
TTGATCAGTCTGAATTCTGAGTTTTTTTTATTTTAAATACGATGAAACCATTTCTGTCTACTGAACTAAT
TATTGTGACTCAATTCAAGAGTGCATACAAATGCAACCTTACTTATCGAACATCAATAAGCAGGAAAT
TTGCCACAGGGACCTTAAACTGGAAAATACACTCTTAGATGGGAGCCCAACGCCGCGCCTTAAAATA
TGTGATTTCGGTTACTCCAAGGTAGTTGCTGTTGGGTATATGGTATTTGTACTTTATGTCAATTTAGA
AGCTTGCATGTTCAGTGGCTTCTTTGTGTTGGATTGTAGTCTGCATTGTTGCATTCGCAACCCAAATCA
ACAGTAGGGACGCCAGCATACATTGCCCCAGAGGTTCTATCACGAAAAGAGTATGATGGAAAGGTA
ACTTGTTCTTTTTAAGTGAAATGTAGTGTACTGAACTATGCGATGCACTAATCATTTTCTATAGTTTAA
GCCTCTGGTTAGACATTTAAAATCAGCCAAGTTCATGTCCATTTCTATGCAAGTGGTTGACTTGGTTC
GTGATTGAATTGATGGCAAGTTGGTTTTCTCTCTTGCATGATTATCTTTGACTTGTGGCCTTCTGCAGA
TTGCGGACGTTTGGTCGTGTGGGGTGACACTGTATGTGATGTTGGTGGGGGCATATCCATTCGAGG
ATCCTGAGGATCCTAGAAATTTTCGGAAGACAATTGGGGTAAGTATTCTTCACAAAGCTTATGAGAA
GCACCATTCATGTAAGTTGGGGTGTGTTTGGATGTTCAATTTGGCTGAATTACGACAAATTCAGGTCC
TTATAGTGAAAATGGCCAATGAAGGGGCGGCACCATTTTAATTCACGATGGAAACCAAACCCTTAAG
TTCAATTCCATGGCATTCGAGTTAGACTTAAACCAGTAGTAATTGCAATTTGAGGGCCAGCCTTTCGT
TGAATCTTTGTTGAATCGAGTAAAATTGCAATTCAATTCAATTGAACATCCAAACATAGCCTCGACTTC
ACCTGTGTGCTCACTTCTCTAACTTCTGTTTAAATTCAATTCCATGCCATTCAAGTTGGACTTGAAATG
GTAATAATTTCAAACTGGGGGCCATCCTCTCATCAACACTTTATTGAATTGAGTACAATTGCAATTCA
ATTCAATTGAACATCCAAACACAACCTTGACTTCACCGACAAGCTCACACCTATGACTCCTGCTTAAGT
TCAATTCCATGCCATTCTAATTGCCTTGAAACAATAGTAATTGCAATTTGGGGGCCAGCCTCTCATTG
ACCCTTTAAGGAATTGGGTGAAATTGTAATTCAATTCGATCAAACGTCCAAAAACAACCTCGTCTTCA
CTGACAATTCACTCCACTGACTTTTGTTTCAAGTTCAATTCCATGCCATTCGAGTTGGACTCAAAACAG
TAGTAATTGCAATTTGAGGGCCTCATTGAATCTTTATTGAATTGAGTGAAATTGCAATTCAATTCAATT
GGATATCCAAACACAGCCTTGACTTGCACTAACAATTTCACTCCCCTGACCCCTAACAGAGGATAATG
ACTGTACAATACTCCATACCAGACTATATACGCACATCAGCAGAATGCAGGCACCTTCTTTCTCGAAT
TTTTGTCGCTGACCCATCAAAGGTATCCTGGACGTTGATTCTCCACTTATTTTTTTTTCTCTAGTTTCA
AACAGAATTATTAAACATTTCTATGTTTCTCATTGAAGAGGATCGCAATCCCAGAGATAAAAAACCAT
CCTTGGTTCCTGAAGAACTTGCCTAGAGAGCTGATCGATTACGAGAAAACAAACTATGAGAATGTAG
GCAGTGACAGCCTGTCTCAGAGCGTTGAAGAGATAATGCGGATCATACAAGAAGCAGGGACACCCG
GTGAAGGTCTGAAGGTTGATGGTCAGTCTGTCGCAGGGTTGGTGGACCCTGATGACACCGACACCG
ATATCGACACTGAGGAAGTCGATGAAAGTGGCGATTTTGTGGCACGGGTGTGA
