# Supplementary material for: Genome-Wide Identification and Expression Analysis of SnRK2 Gene Family in Dormant Vegetative Buds of Liriodendron chinense in Response to Abscisic Acid, Chilling, and Photoperiod
Source: Genes (Basel). 2022 Jul 22;13(8):1305. doi: 10.3390/genes13081305 (PMC9331246; doi:10.3390/genes13081305)
Supplement: Supplementary file 1 [file genes-13-01305-s001.zip › Table S7.pdf]

Table S7. Promoter sequence of SnRK2 family in *Liriodendron chinense*.

>Lchi13910

AGGTCACTAGCCAAACTGCATAGGTAGGATCTAGTTAAAGACGGACTGTAAGGGGTTCTGTCACGCC
CTCGTATTGTAGCAAGACGCAGTTTGGCTCGTGATCCAGACACTCACCGGCTAGCGGTGTCACAGCT
CTTTGAGCACTACCATGATTTATATGTTTTATCCATGCTGTCCATCCATTTTATAAGGTGATTCTCAGA
CATGAGCTTAAATTCGCGGCAGATCTAAATCTCAGATATGCCAAACCACAAAAAAAATAGTAAGAACT
GAAGCACTTGTCATTGAAAGCTTCTCGGGGTCTTCCAGAGTTTTGGATAAGCTAATATTTGGAGCTTT
GGACACCAGCTAGCTGGCTGACGTCCGGGTAACCAGCCAAACCGCGTCCCGCCAAGGTGCAAGTGG
AGTCGTTTGCGTCCTTCCTCTGCGTACATACCGAGCTGTGGAGTAGCTGTTTATCCACTTTGTAAATTC
CATTTATAAGATATGTCATGATACTAAGAATAAGCAGGTCCACCGGTCAAGTCGACCACACCAAATA
ATGAGCTCGTGTTGCATTAATGCAAGAGACCTGCTTCATTCTTAGTCTTATTCTTAGTCTCATGTGGTC
TACCTGAACTTAGAAATAGAATGATAGGATGGAGTGGATAAACAGATACATCACGCGGGCCCTCCA
CTGCTCGGTATGGACGCCTACTGAGGAAGGACGCAATCCAACTCCGGTGCAAGTGGAGGTAGTGCC
CAATCCACGTCCCAGGAGTGCACCCAACATTGAAAATTTTGATTGGAAAAATGACCAACGGTCCTGA
TTCGATATATTCATCTGGCCCATACGACATTCAAAATGGTATTTCTTTTAAAGAAAAGTACGTTTATCA
AACCATTTCTATCTGATGAACGCCCCAGATCTCTCACATGCATGCCACATTAGCACGAATAGAGAGAG
GAGTATTCAGATGTCACTAATACCACTCCCCCATTCCCACTCGGGAGCGGATTGCCTGTGACCCCGG
GCACAGAGATATCTCTGTGCCCGTAGCTGTGTGGGGCCCATAGAGATGTCAGTGACAAATCCACTCC
GTCCATCTGTTTTTTAAGACCACGTTAAGATATCATTATAAAAATAATGCAGATCCAAAACTCATGTG
GGCCATAACACACGAAAAAGTGGGAATTGAACACTTAACGGTGCAAAAAGCTTTACATCAGGCTAAT
ATTTTTTATTACAGTTTATCTGATTGTTAATAATTCTTTGAACGGTCAGTGACAAATCCACTCCGTCCAT
ATGTTTTGTAAGACAAAAATAGGACAGTGAAGAAAAAGTAAGATAGATTAAAAACTCAGGTGGGCC
ACACCAAAAAAACAGTGGGAACACTAACGTCCACCATTGAAACCTTCTTGGAGCTGACCATGATGTT
TATATGCCATCCGAACCGTTCAAAGAATTATTAACAATCAGATAAACTGTAATAAAAAATATTAGCCT
GATGTAAAGCTTTTTGCACCGTTAAGTGTTCAATTCTCACTTTTTCGTGTGTTATGGCCCACAGTGAGT
TTTGGATCTGCATTATTTTTATAATGATATCTTAGCGTGGTATTAAAAAACAGATGGACGGAGTGGAT
TTGTCACTGACATCTATATGGGCCCCACCCGGGGTCACAGGCAATCCGCTCCCCACTCACTCAACCGC
GTGGCATTTCCGTCGTTGAGGTAGGACAAAAGCGTCTATCTAAAACAAAAATACTTTGATACTCTGG
AAGACTGCAAAGGTTCGTACACATGCACTCAAACGATTGCACCGGCATCACATGATAGATCAAAATA
AGCCGTTCAAATCGTGGATGACACTCTAAAAAATCTATAAGCCTAGAATTAAACTAAATTAATTTTCA
AAATCGGAGATTAATATATGACTATTACACAGTTATAATGGAAAGTATTCAACGGTCCAAATTAAAA
ATTGAATCCATTAATAAGATATCCAAAACGAATTATTTTATTAAATTAAAAAACCATCTACAATATATC
CCATGATTTGAATTATTTTATTAAATTTAAAAACCATCTACAATATATGATTTCTACGGTTTAGATTGA
TAAAAGTTATCACAAGTATACAAATCTGTGCGCACGCATATGAACCACCGTACTCTGCCAGAGTATAA
AAGCAACAAAAGGATCAGCCTGTCATTATCCTCTAAAACCCAGGGGCATTTCCGTAAATTCAGAATCT
CTCTCCCCCTTCGGATATTAATGCGATCATCTCCATCAGAAGCTCTCTCTCACGGGCTTCTCTCCTCCAT
CCGTATCTATCTTCCAACCAACGCCACCTCCGCCATCCGATCAGACGCATCCAACGGCTCATCTCTTGC
CACGTACCCTCTTCCTTTCTTTCCTTTCCAGTCCCTTCCCCGATCGCACCTTTTCTATCTCGATAATGTG
GGCAACTGCATCCCACCATCGGCGCCATTAGATAAAAACCGTCGGATCATTTACAGCG

>Lchi00543

ACAACTTTGCTAGGACTACGGTCCTCTACACCACTCTTGTACGTAGTGGTCAAAATACCCAGGTCTTG
TGGGGCTACCGTGTCCATCATGAGAGATCCTCTCCATTCATCAGGTCTTTCCTACAGCTTTGCTGGGA
CTGCAGCCCTCTACACCACTCTTATACGCAGTGGTCAAAACACTTGGGTCCCATGGGGCCATCGTTTC
CATCATGAGAGATCCTTTCCATTCATCAGGTGAGCTTTCACAATTTTACCGTAAATCCTAAAAATCAGC

TTGATAGAAAATGCTGAGGCCTTAGAGAAATGAAACTCACACCTAAAACCTTTAAACTCACAAGACA
TGGGCCACCTAAGATTTTACACCCCCCCCCCCCCCCCCCCCCCCCCCCCCACCAAAAAAAAAAAAAAAAAAA
AAAACATTGCATGGGTCATAGGTGACTTTGGAAGTTTTTGGGTGGGTCTATGGTTTGTCCCACCCAA
CAATTGTAAAAACTGATTGGCATCCTATATTATGTGGGACTCACTTGATGATGGGTTGAATAGCATAT
ACACAACACGATGGACCCTACCATGCAGGTGAATACACATTTACATGTATATGAACAATAGACGCAT
ATTGGGTATACTCGGTGGGGCCCACTGTGATGTTTGTATCTTATCCACACATCCATATGTTTTTCCAGC
TCATTTTAGGGCATGGGCCCCAAATTGAAGCATTTTCAAAGCTAAAGTGGACCACATCAAAGGAAAT
AGTGAGATAACGACATTCACAGTTGTAACCTTCCTAGGGCCCATAGTGATATTTATTTATTAACCAAC
GTGCTCATAAGGTCACACAGATAAAAGGAGAATTACAAATATCAGCCTGGTCCAAAACTTTCACATC
TCTAAAGAAATCAATTTCCACTGTTTCTATAGTGTGGTCTACTTGAGCTATGAATATGCTTCAATTTTG
TGCTCATGCCCTAATGGCTGGTTGTTTTACCATTTGCCATGGTAAGTAGTGATAAATGGGTTACTATT
AATTACCACGGTAATTTCATACAAGCCCTCCTTATGTGTTGATATCTTCATTAGAATTGAGGAAGAGA
ACTACGTATGTACATTCTGTGGGCCCTACCATGGTATATGCATCCAATCCATACTGTCCACTCGTTTTA
TCAACTCATTTTAGGCCATGAGCCCCAAAAATGAGGGAAATCCAAAGTACATGTGGACCACACCATA
GGCTAAAGTGGGAATTGAATGCCTTCCATTGAAAACTTTGGATCAAGCTGATATTTATGTTTTTTGTT
CATTTGCGTCATTATAACCTGCTGAATAGGTTGGATGATAGATGCACATCATTGTAGGCCTTATACAA
AATTTCACTTTTAACTGTGGACATCAGTATCTTCTATTTGTCATGGTGTGGTCCACTTGAGGTTAAAGA
GTCCGGATCCTCCATTGGTTGCAAAATAGGAAATCCCTTGTTTGCAACCTTTTCATTGGTCTACGTCAC
ACTGCATGACGTCAGTGTGATCAAGTTGCATTGAGAGTGTGATTAGGTTGCAAACATATATGACTCT
AGTAAATATACACAAATTACTAACATTTATTATGGTAAATGGTAAAACAAGCAGGCCTCTAGATTCCA
GTGCATTTCAACCAAACACATTTCAGGATTTTTTTTAGAATTCCAACAAGGGGTGGTTAATGAGCCAA
ACTATTCGTGAACGGTTTCAGTTCGGTTCAATTAAAATTTGTTTGAGTTCGTTTGATTACTAAACAAAT
CAAACTCTAACCTTATTTTTAGGTTTGATTAGTAATCAAACCAAACTCAAACAGGCATGAGTTCGGTT
CGGTTTGGCTTGATAGGGTTTGTTTATAGCTAAAAAAAGCATTGTTTTTTTATAATATAGTCAACTAGA
GATTTGGATCTGCTTCATTTTTTTACTCCTGTCATGAAATGATTTGATGAAATGTATGAACGGAGTGG
ATATGGAATATATGCATCAAGGTGGGCCCTATGTACGAGCCTCACCGTCTTGACTTTTTTGGATATGG
AATATATGTTGCATGATGGCCCCACAACAAGCATATCCAAACCTCAAGTGGACCGTGCTACGCCGTC
CAACCCACAATGAATTCTAAAGGTGCACCATCTCATTTACACAAGCTTTAAAATGTTGCATATAGGGG
TGGTTAATGAACTAAATTATTTATCAACCGTTTGAGTTCGATTAAAGCTTGTTTGAGTTCGTTTGATTA
GTAAACAGACTAAACCCAAACTCAAACTTGAGTTTTGGTTATCAAACTCAAATCAAACAAACAATGTG
GTGTTCGGTTTGGTTCCGTTAATTTACACCCCGGAAATGAGACGGGTCCTCTAGTACGCTGCCAAAA
AGACCAAAATAACCTCCATTCGTCTCCGTATTTCCAAAACACGTGCCACGCGAACCCGCCTAAAGGAC
TTCCCTTTCCAGAAGATCAAACGCCAAAACCCTAGCGCAGAGAGCAGAGAGAG

>Lchi25623

CGTCCTCTCCCCTGCTTCTCCTCTACAAAGATCTGAACAGTCGAATCTCACAAGTGTTTGGGATGATTT
TTCACATTCTCCAATCTGGGTGTGGCCTGAAATAAGAGATCTCGATGTCTCATCACCTTTCCAGATCTT
GGGAGCTTTTCGAATGTTCTTTGAGAGTGTGAAAACGAGGAGGATTTTCTAGAGAGAGAGTGATTG
GAATTTCTTTAATGGAGATCACGTGAGAGGGAGGGATTCCATGCTTTGCGTAGATCAGATGATTTTT
CTTTTCTTTTTTCTTTTTGGGTCTCTCTCTGCACTTAGATCTTGAAAATCGCCGGTTTGTGAGGAGAGT
GAAAGATGGGTTTTTGACTTTCTTTCTTTTTTTATACAGGAATGGCGTTGAGAGCGGTTTTGGAGAGG
AATTTGGGCGGTTTTGGGATACTTTGGAGGTGGTTTTTCAAAGGAAGAAAGAAAGAAAAAGAAAGC
AGTAGTCGGATGGAAGAATCTATCTTTAATATGGAGTTCTTTCATATTTTATTTTTTTCTGGGAGTTTT
TTAATTTCTTTAGTTCGAGATTGGTTCGTCGCGAAGTACGAATATATACAGTTCATCGCGATGGATGA
ATGTCCACGTGGTCGCGTCAGTGGAACTGCAGAGGTTGTGCAATTCGTGGGGCGATGAAAGAGTCG
TGGTTTATTGCTCCCGGAGTAGTCTACAGTAGATTCGGCTGCATCGTGTGGTTTATCTCTCCCGTACA
AAACGAGTGGTCGGTAATCTGCAATTCCCCAAGACAAATGGTTTTGATCGAATGATTTTAGCCGTTG
GTTAAAAAATTTGATATGCGCGGATGGACCTGTGGAAGCATGTGGCAGCAGGCGCGAATTAGGTGT

TACCCCGGGAACAGCTTAGTGGGTGTTACCCCTACCGTATGGCGCACATGGATGATATTTTGCATATC
AATTCCGTCCATCCGTTTCTAAAGCCCATTTTAGTAAGATGTTCCAAAAATTATATAGATCCAAATCTC
AAGTGGACCATACCACTAGAAAGAGTGTGATTGACTGCCCCACCGTTAAAATTTCCAAAGAGCTCAT
CGTGTGGCTTTGGTAGTGTTTATTTGAAATCCAAGCTCTTTATAATGTGAATGAGGTCTTGATGAAGG
TAAAATATAAAGATCAGATTCATCCAAAACTTTTGTAGCCTACAAAACGTTTTTAATTGTTATTCATCA
GTGTTTCTAGTGGTATGGTCCACCTGAGATTCGGATCTTCTTATGGCTTTGAATCACATCCTAAACTG
ATATGAAAAAACACATAGACGGATTCGATATATAGAAAATACATCACTATGGGCCCCACACGGTTAG
GGTAACACCCACTAAGCTGTTCCCGGGGTAACACCTAATTCGCGCCCTGTGGCAGCTGGACGGTGTT
GTAGCACGTGCGTTCGAAGTATCCAGAGGGATGCACGTTTACCTTATTCGGCGGAAACTCTGGCCGA
ACATTACATAGGTGGGCCACACATGTCCAGAAACAAGGACGCGGAATTGGAAGTTCCTGCGCTGGG
AATATAGGTGGGGCCCACCGTGATGTTTGTGAGAAATCCACGCCATCCATCCGTTTTGTGATATCATT
TTAGATTGGGCCGACCGAAAATGAGCCGGATCCAAGACTAAAGTGGGACCCGTGAATAGGGAAATT
CCTGCTGTTGAAATCTCCTCGGGGACTACGGTGATGTTTATATGCCACCCATACCGTTAATAATTTCA
GTTTCCACTGGGATGAACTGAAAACACAGATATTAGCCTGATTCAAAACTTTTGTGGCACCACAAATA
TTTCAACTGTGAACTTTCAATCCTCACATTTTTGCCATTTGAGTCTTGGATCATCTCATTTTCGGTTGCA
CATCCTAGAATGATCTCACATATGGACTGCGTGGATTTCTCACAAACATCACGGTGGGCCCCACCTAG
ATTTCCAAGTAGAGGAACTTCTTTAGCAGGAAATCTACGTCCGGAAACAATGGACACGGATTGCCTC
TCCGCAGGCATCTCCTGCACTTGGGATCTATGTGGGGCCACCATGATGTTTGTGAGAAATCTATGCC
AGCCAACCTATTTGTGAGATCATTTTACGTTTTGTGAGATCATTTTAGGCATTGCAACAAAAAATGAT
CGGGATCTAAGACTCAAGTGGGCTGCACTAAAGCAAAAGGTGGGTAGGGAAATTCCTACCGTTGAA
ACCTTTATGGGGTCCACAGTGATGTTTATGTGCCATCCATACCGTTCATTATGTCATTCCTCCTGAGAT
GAACTTGAAACACAAATATTATCCTGATTAGAGCTGTACACAAGTCGAGTTGAATCGAGCATCATCTT
GATCGGTGCTCGATTTGAACTCACATACCCATGGAGCTGGGCACAAACCAAGCCAACTCGAAAGCTC
GGCTTGGATTGGTTTGAAAAAGCCCACCTTGGATTGGTTCAAATGGAAGTTCAAGCCAAACCAAGC

>Lchi12999

CGAGGAAACGAATCCTACGGCTCTCCTTCTCTGACGATGCCGGGAGAGGGAGTCTCTGCTTACATGA
TCGAGCAGCAGAGAGCTCGGATCGAGATGGGAAGCAAAAGGAAGGGGAAGATGACGTGGCTGGA
TAAAGACCGTTGGATCAGATGGATCGATCGAGAAGGTGGGGTTGGTCGAAGAAAGGAGAAGCCCG
TGAGAGGTTGAATTGCAGAGAGAGAGAGAGAGAGAGAGGAGATATTTTTCTGGATCGGCGATCGG
AAACTTGATTAATTAAATATCCCAGGCCCACGCGCGTGTAGAGATCTTCCTCCACGCGCAGTCACACG
TAGAAATATGAAACACGTGTGAGATATCTGATCTTTCCATCAGGTTGGAGACACTGTTTAGATCTTCT
GGAGTTTAATTCAGGATGGTTGTATCTTGACGTGGGAGTAAGTGTACAAAATGAAAGAACGGCTGA
AAAACACTTCCCTAGCAATTTGGTTGTCCTGTCGGAGTGGGGCCCACTTAAGCAGTAAGAAGGCCCG
ATTTTAGATTGGGTAATCAAAAAAGGTGTGGCCCACTTGAAGGAAAACTTCGATCTCGAACACGTTT
TACATATTTTGAAGACGTGCTTGCGTGTGGATGGGCAGAAGTTCACACGCGTGTTGGGTTAGCAAAT
TTCTGGATTAATATCTTGCAAGTGGGGGTTGAAATGACGGAAATGTCCTTGGAGTATGGACGAATGA
CTAGATTGACAAGTGGGTCCCATGGTTCGGTAATCTGGACCATTAGTATGCTGAACCCACCGTGGAT
GGACCATTTCCCAATAAAAAATCCTCTATAGGACAATTTCAACGTTCCAAATATCAGATGCAACTAGA
CGGTTGAGAAGAAGAATGTAGCAACTTTCAACCGTCAACTGAAACCATCACAATAAATTGATGGATA
GGATCTTTTTATAGAACATTTACAGTGGAGAGAATCAAACCAATGGTCTGGATTACTGACCATAGGC
CCCACCAATCACAACTGAAACTAGAGTGTATGCTTGCAAAGACGCGTCTGCGTTCCGACCATCTGATT
TTTTTAAGTGGGGGTCCTCACCATTGCTTAGAATCGGTGAGCTTTCAACTGGAGTTGACCGCCTGTGT
GACCAATCAAAGAGCGAATCCCCATTTTTCTTACTCGCGCAGATCAGAAAGAGGCATCAAATAAGAA
ACTAGCTACGCGCCTGGCGCCCCCGGTCCCCCACGCGGCATTGTATGTGAGATCTGGACCGCTTATT
AGACAAGTCCCACTCGGCCACTGGCCCAGAAATCAGAATGGTCCACACATCAACTGAGGCCCCACAT
GCATGGAAAAACTGTATGGCTATAAAAAATTAGCCAACGGTCCACCTACACTGTTTAGATTGTGGCC
CACTCGAAGAAAGGAACGGCCTAAGTTTTGGCCAGACCGCCGGAGTACGGGCACCGATTGGGTACT

ACCCCCAATCAGGACCTAGCTAGAGACGGACGGTCCTGTCAGGGGCCTCTGTGGTCCCAACGTAATG
TATGTGTTTTATCCACGCTGTCTATTCAGTTTGACAGATCATTTTAAGGGATGATCCCAAAAAGAAGC
AGATCCAAGGCTAAACTAAACCACACGGTCCACACCACAGGACTCAACGGGGATTAAAAGTAAACC
GTTGAGAACTTTCCTGAGGAGCACAGAAGTTTTGGGTCAATATTATATTTATGTTTTCCCTCTCATACA
TGTCTATGTGGCCTTATGAACACGTTATATGGCAAATAAACATAACTGGTAGGCCCATGAAGTTTTTA
AGTGTAGGAATTCAATCACCACTGCTTCTTGTGGCATGGTTCACTTGAGTCTTCAATCTACCTCCCTTT
TTTCTAGCTCATGCCCTGTCAAAAATAAATGTACTACTAGGTACGTGTGGGGCAGGTCGAAACGCCC
AAGGTCCTATTCATTTTATTTTTTTCCAAGCTGAGTAGTAGGTAGCTGATTTTCATGAATGGGAACCG
TTCATCCAGTGCGCTCACTTTGTGCGGGGAGCAGATTGCTTGTAACCCAGGGCACGGGGCTCTGTGT
GGCGGCCCACATAGATTTCCATGACAAATTCACTCTGTCCATCTATTTCTAAAGACAACAATAAGACA
GGAGCCTAAAAAATAAGACAGATTAAAATTGGTGTGGCCAATGGCGCAAAAAATAGTTGAGTTTGA
GCACATGAGGGTGACAAAAATTTTGTATTGGGATGATTTTTTTGTCTTCAGTTTATCTGAGAGGTAAC
AATACTGTGAACGGTTTTGGATGGCATATAAATATCATGGTCAGCTACTGGAAGGTTTCAACTTTGA
AGTCTCCATTTCCACTGTTTCATTTGGTGTGGCCCAATTAAGTTTTGGATCTGCCTGATTTTTAGATTT
ATGTTTTATTTGGTCTTTCGAAACAGATGTACGGAGTAGATTTATAATGGACATCTGTGTGGACCCCA
CACAGCCCCAGGCAC

>Lchi01348

CTTAGCGATAATTAAGTAACTGGTAATTGTAGTTGCATTCAAAGTCTACGCTCACACGCTGAATTTAT
TTTAATAAAGTGTTTTACTCGAAAATGTGATATGCAACCTGTTACTTACAGCTGATTATAAGTGTTTCA
TTTTTTTATTTTATTTATATTGAAAAGTCATTTCATACGAAAGACAAAAGTGTAATTTATCATTAGATA
AAGAACCTAACCCTCTAAATATTACCTTAATATTGGAAGATAATTGTGTAAGTGGTTGAATAGGCGAT
CGATCTATACAGATCCTGGTCATATACTGCAAATCCTTACGTATTTACTTAAAATTATTGTTGTTCGGT
TTGATTGGAGCCACATGGTCGATTCTAGCAAGCTTGCACATACCTCACACGCATGACCAAAATTGCAA
GCAACATTGAATATGCTTCAATTTTGGTCTAAACACTCTAAAACGGGACGCAAATTGCTTGCGAAAGC
CTTTCATAGGCATCATCAAGCTCGGAATCTAGGTAGGGCCCACCGTGATGTTTGTGAAAAATCTACG
CCGTCCAACCATTTTTTGAGCTCATTTTACACTGTGTGACAAAAAATGAACTGCATCTAAGAATCAAG
TGGGTGGAAAACATGATAATTGAACTTCCACAATTGAAATATTCGTGGGGCCACAAAAGTTTTGAAT
CAGGATAATATTTGTGTTTTCAGTTCATCTCAGTAGGAATGATATTATGAACGGTATAAATGACATAT
AAACATCACTGTGGACCCATAGAGGTTTCAACGGTAAAAATTTCCCTACCCACCTTTCACTTTAGCAA
GGCCCACTTGAGTTGCTTTGAAGGCATCTCCTGCCCTCTGAACCTAGGTAGGGCCTACCGTGATGTTT
GTGAGAAATTTACTTCGTCCAACCGTTTTGGGAGATAGTTTAGGCCGGGTGACCAAAATTGATCCAA
GACTCAAAGTGGGCTCCAATAAAGCAAAAGGTGGGTAGGGAAATTTCTACTGTTGAGACGTCTATG
GGTCCACAGTAATGTTTATATGCCATCCATACCGTACATAATGTCATTCCTACTGAGATGAATTGAAA
ACACAAATATTATCCTCATTCGAAACTTAAGTGGCCCCACGAATATTTCAACTGTGGAAGTTCAATCG
TCACATTTTCAGCCCACTTGAGTCTTGTATCCTGTTCAATTTTTGTCGCAACGCCTAAAATGATCTCAC
AAAACTATTAGACGGCGTAGATTTTTCACAAACATCACCGTGGGCCCCACCTAGATTCCCAGGCAGG
AGATGCCCGCGTCGGGGGGGGTTGCGCACAACAGTTTACCCGGTACGGTAAAAGCGTACTGAGTTA
GTCAGTAGGCAATCCGATTTCATAGATTTCCACGCGAGAAGGTCTCAGGTCCAACCGGTTGGACCAC
AAATTACGGTTCACTAAGAAGTTAACTTATAACCTACTAAGTGTGTGCTGCATACAACCCTTCCAATA
AGTTGGATCCAGCATGTAGATTTTCTTCTGAAGAAAATCGGCCCCATATACTCATTAGGTGGTCCCAA
TATAGAAAACAATTTCCATCCAATGATAAATAGAAAAATACACTATTGTTCACTCAATGAGTGGATGT
CTCTGATTTTTCCATAAAGAGATTCTTAAGAAAGGACCAACCGATTAGATGGATTGGATCTCATGATC
GCATGAAAAGATGGAAGTTATCTGTTGGCTGTGGACTGTAAATGGTGGTCCAACCGGTTGGACTATA
GATCTTTTCTTTCGTAAGTATTTTTTTGGTGAAGTGGTTAGTTATTTTAGGGGAGTTATTTTATACTGT
GGCCGCCTATGGCGCTTGGTACTCAGGCACTTGGACATGGTACACGTGGAAAATATTAAATAAAATC

AACCGAATAAAATGATTGAAATTACTACTGTTTAAGTTACAGTAAGAAAAATTGTATTGATTGAAGG
ATCCTGACCTCTGAGTCTTGGACACTTGTTTGTTGGAATGAGATCATTGGATTTTTCATTTTTAACCGT
ATAATAGAATATCAACCTATCTGGTATTAAGATAATCAAATAAGCTTAATTTTTAGTTTTATACTTCCA
CAAATGATGTGCGTAATTTGGACGGCTTTATTTGAGTGATTGTATTCCACGAATGCAAGCCTCAGCAA
TTTTAAATGCCTGCGTATCATCCATCACCCTCTGGCAGAGTATCGAAGTTTTTATCTTATTCGAGTTGA
ATCTCTGTTGGCTCTCTCCCTCTCTTTGGCCTGTTTGGGTGCTTTGAGCTGGTTTCTCTCTCTCAGTTCT
TTGAGCTGGGTTCTTTCTCTCTCTATTGATTAGCTTGGGTTCTTTGAGCTGGGTTCTCTTTTTCTCTCTT
GGTTCTTTGAGCTGGGTAACTGGGCTCTCTCTCTGTATCTGGTTACTTTTGAGTTCTGGAGTTGGGTT
ATCACTTTTGCTCTCTTTGCATATCTTATCTTTTTTGGCCTATAGGTGGAGT
