# Supplementary figures and images for: Genome-Wide Identification and Expression Analysis of SnRK2 Gene Family in Dormant Vegetative Buds of Liriodendron chinense in Response to Abscisic Acid, Chilling, and Photoperiod
Source: Genes (Basel). 2022 Jul 22;13(8):1305. doi: 10.3390/genes13081305 (PMC9331246; doi:10.3390/genes13081305)

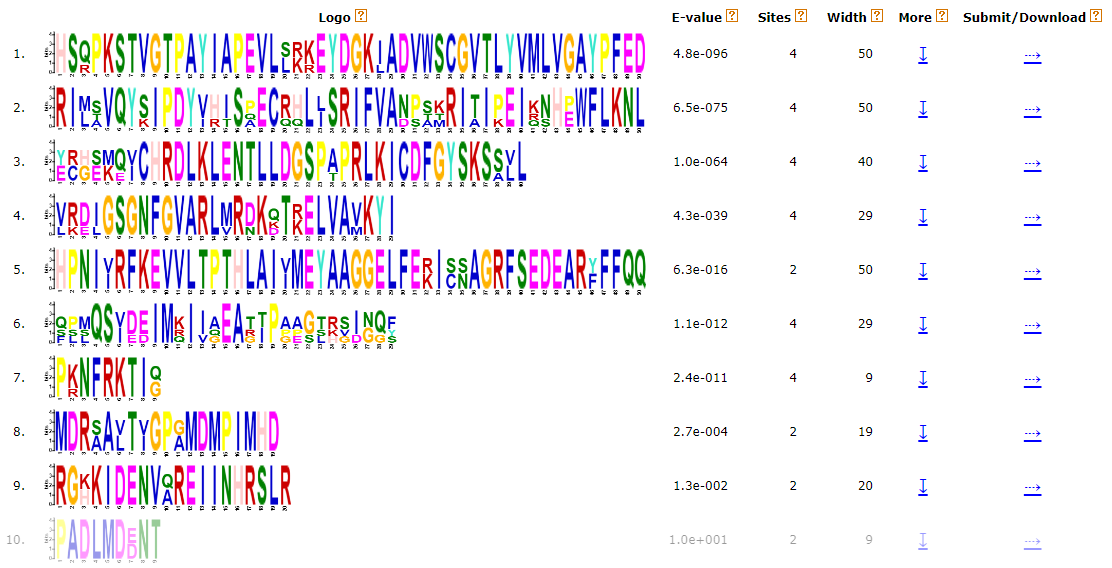

Supplement: Supplementary file 1 [file genes-13-01305-s001.zip › Figure S1.png]
